# Identifying care gaps along the HIV treatment failure cascade: A multistate analysis of viral load monitoring, re-suppression, and regimen switches in Zambia

**DOI:** 10.1371/journal.pmed.1004720

**Published:** 2025-09-03

**Authors:** Kombatende Sikombe, Noelle Le Tourneau, Brian Rice, Jake M. Pry, Sandra Simbeza, Laura K. Beres, Anjali Sharma, Njekwa Mukamba, Alison Wringe, James R. Hargreaves, Jacob Mutale, Carolyn Bolton Moore, Izukanji Sikazwe, Elvin Geng, Aaloke Mody

**Affiliations:** 1 Implementation Science Unit, Centre for Infectious Disease Research in Zambia, Lusaka, Zambia; 2 Department of Public Health Environments and Society, Faculty of Public Health and Policy, London School of Hygiene and Tropical Medicine, London, United Kingdom; 3 Division of Infectious Diseases, Washington University School of Medicine, St Louis, Missouri, United States of America; 4 Sheffield Centre for Health and Related Research (SCHARR), School of Medicine and Population Health, University of Sheffield, Sheffield, United Kingdom; 5 Department of Public Health Sciences, School of Medicine, University of California, Davis, California, United States of America; 6 Department of International Health, Johns Hopkins University Bloomberg School of Public Health, Baltimore, Maryland, United States of America; 7 Division of Infectious Diseases, University of Alabama, Birmingham, Alabama, United States of America; University of Southampton, UNITED KINGDOM OF GREAT BRITAIN AND NORTHERN IRELAND

## Abstract

**Background:**

Timely response to treatment failure is critical for improved outcomes and viral re-suppression among people living with HIV, but care gaps along the treatment failure cascade can occur due to delays by both clients (e.g., retention and adherence) and health systems (e.g., fidelity to viral load [VL] monitoring guidelines). We used multistate analysis to identify drivers of implementation gaps in the treatment failure cascade, including time to HIV VL monitoring, re-suppression, and regimen switches, in Zambia.

**Methods and findings:**

We used national electronic HIV health records to identify adults on antiretroviral therapy (ART) for more than 6 months who experienced treatment failure (VL ≥ 1,000 copies/ml) at 24 clinics in Lusaka, Zambia, between August 2019 and November 2021. Using multistate analyses, we examined how care evolved after treatment failure, accounting for transitions across the treatment failure cascade over time, such as return visits, repeat VL testing, treatment interruptions (>60 days late for visit), and viral re-suppression. Analyses were stratified by ART regimen at cohort entry: tenofovir disoproxil fumarate/lamivudine or emtricitabine/dolutegravir TDF/XTC/DTG (TLD) and tenofovir disoproxil fumarate/lamivudine or emtricitabine/efavirenz TDF/XTC/EFV (TLE). We repeated analyses to assess switch to second-line therapy among those with consecutively unsuppressed VL test results who were due for regimen switch. Among 179,855 individuals on ART (143,857 with documented VL), 7,916 (4.4%) had a documented elevated VL and drug regimen at the time of treatment failure (52.3% female, median age was 36.7 years (IQR 29.9–43.6), median time on ART 3.3 years (IQR 1.7–6.6), 54.6% on TLD and 45.4% on TLE). Among those with treatment failure, 72.2% (CI 71.3, 73.0%) had returned to clinic 6 months after initial elevated VL was drawn. After one year, 70.1% (CI 69.3, 70.9%) had a repeat VL, 16.6% (CI 15.9, 17.2%) experienced treatment interruption, and 11.4% (CI 10.3, 12.4%) returned to care without repeat VL testing. Among those with a repeat VL, 85.0% (CI 83.9, 86.1%) on TLD and 58.2% (CI 56.8, 59.8%) on TLE had resuppressed. Among those due for second-line switch, 27.9% (CI 24.1, 31.5%) on TLD and 66.6% (CI 64.5, 68.9%) on TLE had changed regimens after one year while 52.4% on TLD had a third VL repeated prior to switch (CI 47.2, 57.4%) (68.0% CI 61.6, 75.2% suppressed of those with repeated VL) compared to 32.1% (CI 29.9, 34.1%) (40.7% CI 36.1, 45.4% suppressed) on TLE. This study was limited by the inability to capture all aspects of care delivery related to treatment failure, such as outreach, enhanced adherence counseling confirmation, and provider rationale for delayed VL rechecking.

**Conclusion:**

After treatment failure, we identified substantial delays in returning for adherence counseling, treatment interruptions, and missed opportunities in rechecking VL status or switching to second-line therapy in routine care in Zambia. Among those who did have VL tests rechecked, re-suppression rates were significantly higher among individuals on TLD compared to TLE. To optimize response and outcomes after treatment failure, strategies must prioritize and target both client and health systems behaviors to meet the care needs in the modern era of TLD.

## Author summary

**Why was this study done?:**

- Prompt, guideline-concordant responses to treatment failure are essential for improving viral suppression and treatment outcomes among people living with HIV with viremia.
- However, in routine care settings in low- and middle-income countries, including Zambia, significant gaps persist along the treatment failure cascade, such as delays in initiating enhanced adherence counseling (EAC), repeat VL testing, and timely regimen switching. These gaps may stem from both client-related challenges, like treatment interruptions, and health system factors, including provider adherence to guidelines.
- This study aimed to characterize these implementation gaps, particularly in the context of Zambia’s transition to dolutegravir-based first-line therapy.

**What did the researchers do and find?:**

- We leveraged electronic health records between 2019 and 2021 from routine care from 24 public HIV clinics that serve 179,855 clients, including 7,916 individuals with documented treatment failure who were followed in this study. We applied a multistate analytic approach to comprehensively assess individuals’ care journey with respect to VL monitoring, treatment retention, regimen switching, and, ultimately, viral suppression.
- We found that only 36.4% of patients had a VL rechecked within 6 months of failure, and only 50.1% had documented viral re-suppression at one year, driven in large part by delays in returning to clinic, treatment interruptions, and missed opportunities to recheck VLs after return.
- Among those that did have a VL rechecked, rates of re-suppression were significantly higher for those on dolutegravir-based regimens (TLD) compared to those on efavirenz-based regimens (TLE), despite otherwise having similar care patterns.
- Fewer than 25% of those who ultimately switched regimens did so within 6 months of initial treatment failure, the upper limit of guideline recommendations. Providers were much less likely to switch individuals on TLD to second-line regimens compared to those initially on TLE.

**What do these findings mean?:**

- These findings reveal major implementation gaps in the treatment failure cascade under routine programmatic settings that preclude viral suppression and include (1) delays in proactive outreach and prompt return to initiate EAC, (2) ongoing treatment interruptions, and (3) missed opportunities by the health system to recheck VL test status or switch to second-line therapy.
- Given the global shift toward more potent dolutegravir-based regimens with high barriers to resistance, these findings suggest modern approaches to treatment failure should prioritize strengthening health system processes for timely outreach and integrating person-centered strategies to improve engagement, rather than focus on clinical decision-making (e.g., concerns for resistance and switches to second line) alone.
- This study was limited by the inability to capture all relevant aspects of care delivery related to treatment failure, such as outreach, confirmation of the delivery of EAC, and provider rationale for delayed rechecking of VL test.

## Abstract 3

Kombatende Sikombe and colleagues investigate the hurdles to continuous HIV viral load suppression as a function of care gaps in a retrospective, multi-state analysis of persons with HIV treatment failure in Zambia.

## Introduction

As HIV programmes have scaled up and access to antiretroviral therapy (ART) improved, sustaining viral suppression and improving outcomes among individuals with viremia has become a critical priority [1]. Existing evidence suggests that across Africa, 50%–70% of individuals with treatment failure (i.e., viral load [VL] ≥1,000 copies/ml) have documented re-suppression after receiving enhanced adherence counseling (EAC), a structured counseling intervention to identify and address barriers to medication adherence for 3–6 months [2], though re-suppression rates are likely higher among those on integrase-based regimens [3–7]. Furthermore, with ongoing shifts from tenofovir disoproxil fumarate (TDF)/lamivudine or emtricitabine (XTC)/efavirenz (EFV) (TLE) to TDF/XTC/dolutegravir (DTG) (TLD) as first-line ART regimens, it remains important to understand how these recent transitions impact fidelity to treatment failure algorithms, rates of second-line switch, and overall re-suppression rates [8,9].

Identifying care gaps and their contribution to suboptimal clinical outcomes is essential for developing effective strategies to optimize care delivery after treatment failure [10,11]. Rates of documented re-suppression reflect a complex interplay of client-level challenges, health system constraints, implementation issues, and biomedical factors (i.e., drug resistance). On the client side, inadequate adherence and drug resistance remain concerns, while delays in returning to care for EAC and poor retention that also delay VL testing may also be important drivers of treatment failure [12]. On the health system side, challenges include inadequate outreach to reengage individuals after identifying treatment failure, poor quality EAC that could exacerbate engagement challenges, delays in completing EAC, failure to order repeat VL monitoring, and missed opportunities to switch to second-line therapy when appropriate [12]. By examining transitions through key steps in the treatment failure cascade, including repeat VL monitoring, initiating EAC, and switching treatment regimens, we can gain insights into health system and client factors that drive outcomes. This approach also helps assess whether the transition from TLE to TLD has influenced care patterns, provider behaviors, and outcomes.

In this manuscript, we employ multistate analytic approaches to comprehensively examine care journeys of people living with HIV who experience treatment failure during routine care in Zambia. Multistate analytic approaches account for client transitions in between different care cascade states over time and can help to better characterize care patterns and identify the magnitude of care gaps at different stages [13–15]. Using data from 24 public HIV health facilities in Lusaka, Zambia, we examine the longitudinal dynamics of clients’ care engagement after treatment failure with respect to return clinic visits (as a proxy for EAC initiation) and retention in care, repeat VL monitoring, switch to second-line therapy, rates of re-suppression, and how these differ on TLE versus TLD [13,16]. We hypothesize that delays in key steps of the treatment failure cascade may significantly contribute to suboptimal VL suppression rates among individuals with treatment failure in Zambia.

## Methods

### Ethics statement

Approval to conduct this research was granted by the Zambian Ministry of Health (MOH), National Health Research Authority, and the University of Zambia Biomedical Research Ethics Committee (008-03-19), the University of Alabama at Birmingham (300003282), and the London School of Hygiene and Tropical Medicine (21384). We obtained a waiver of consent to use Zambia’s electronic health records for measuring clinical care and outcomes. The research in this paper was not prespecified and consists of a secondary analysis of preexisting data. This study is reported as per the Strengthening the Reporting of Observational Studies in Epidemiology (STROBE) guidelines (S1 STROBE Checklist).

### Study population and setting

We assessed guideline concordance of VL monitoring and switch to second-line ART regimens among adults aged 18 years or older who had their first documented elevated VL test (≥1,000 copies/ml) between 12 August 2019 and 12 November 2021 after being on ART for at least 180 days and had a documented first-line drug regimen at the time of treatment failure. This analysis was done as part of the clients who were receiving HIV care from 24 clinics in Lusaka operated by the Zambian MOH and supported by the Centre for Infectious Disease Research in Zambia, a Zambian nongovernmental organization. Lusaka province in Zambia has an HIV prevalence of 14.4% with estimates suggesting 89.7% diagnosed, 87.9% on treatment, and 85.3% suppressed within the province [17].

Zambian HIV treatment guidelines during this period indicated VL monitoring at 6 and 12 months after initiating first-line ART, followed by annual monitoring thereafter. Individuals with an elevated VL test undergo three sessions of EAC, typically at 2- to 4-week intervals, and, after completion, should have their VL test repeated within 3–6 months after the initial elevated VL test [2]. Individuals with a second consecutive elevated VL test are recommended for switch to second-line ART regimens; those who resuppress return to routine monitoring schedules. Prior to 2018, non-nucleoside reverse transcriptase inhibitor (NNRTI)-based regimens (i.e., TLE) were recommended as first-line regimens [18], but integrase inhibitor-based regimens were scaled up after 2018 (i.e., TLD) [1,2]. Throughout this period, guideline-recommended second-line regimens included protease inhibitor (PI)-based (i.e., ritonavir-boosted lopinavir [LPVr] or atazanavir) and switching TDF to zidovudine (AZT) (e.g., AZT/XTC/LPVr as a second-line regimen), but integrase-based regimens (i.e., DTG) were informally used at the time (and formally incorporated in guidelines for TLE failure only in 2022 after the study period) [1,2].

### Measurements

Sociodemographic (e.g., age, sex, clinic site), clinical (e.g., VLs, ART regimen, enrollment CD4 count, World Health Organization [WHO] Stage), facility-level (e.g., size), and visit history (e.g., HIV clinic enrollment date, ART initiation date, follow-up visits) measurements were obtained from the national electronic health record and laboratory systems used in routine HIV care in Zambia. At the time of the study, the electronic health record was populated by providers completing standardized electronic clinical forms: laboratory results were printed at the main laboratory, sent to the clinic, and inputted in the electronic health record. Trained MOH data clerks entered all information into the electronic database on an ongoing basis, and performed routine data quality audits and updates at least quarterly to ensure relatively high-quality data. Although routine visits to the clinic were documented, whether an individual received EAC was not documented in the electronic health record. In the absence of consistently documented EAC dates in the dataset, we used the timing of return visits after an initial elevated VL test as a proxy for EAC initiation.

### Statistical analyses

#### VL monitoring outcomes after initial treatment failure (i.e., VL ≥ 1,000 copies/mL).

**Fig 1:**
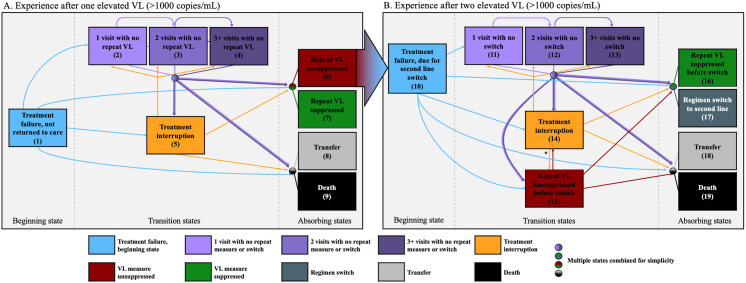
State transition flowchart for multistate analysis. This figure depicts all possible transitions between care states over time. **A.** At each time point after cohort entry, clients were categorized into 1 of 9 mutually exclusive and exhaustive states. For the first stage analysis, those with one elevated VL ≥1,000 copies/ml could be in states: (1) treatment failure, with no return visit and not returned to care, (2) 1 attended visit for EAC without a repeat VL, (3) 2 attended visits for EAC without a repeat VL, (4) 3+ attended visits for EAC without a repeat VL, (5) treatment interruption (i.e., >60 days late for visit after an elevated VL), (6) repeat VL not suppressed (≥1,000 copies/ml), (7) repeat VL suppressed (<1,000 copies/ml), (8) transfer, and (9) death. **B.** For our second stage analysis, those with a second elevated VL ≥1,000 copies/ml (6) were then recategorized as (10) now due for second-line switch and were categorized into another 1 of 10 mutually exclusive and exhaustive states at each time point after the second elevated VL: (10) treatment failure, due for second-line switch and with no switch or return visit, (11) 1 attended visit for EAC and no switch made, (12) 2 attended visits for EAC and no switch made, (13) 3+ attended visits for EAC and no switch made, (14) treatment interruption (i.e., >60 days late for visit after an elevated VL), (15) repeat VL not suppressed, (16) repeat VL suppressed (<1,000 copies/ml), (17) ART regimen switch, (18) transfer, and (19) death. This figure depicts all the possible transitions clients could make from each state. Note: Clients attended visits presumably for EAC, though this is not confirmed. Abbreviations: EAC, enhanced adherence counseling; VL, viral load. *If a client had a prior unsuppressed repeat VL and had a visit after treatment interruption, they were classified as unsuppressed at that visit.

We used multistate analytic methods to describe the longitudinal care and VL monitoring of clients after treatment failure (i.e., their first elevated VL test ≥1,000 copies/ml). We tracked transitions between the various care states that occur over time up until a subsequent VL measure was repeated per guideline recommendations (e.g., return visits without repeat VL test, treatment interruption [>60 days late for scheduled visit], repeat VL test suppressed versus unsuppressed) [13]. Time zero (i.e., cohort entry) was the date of first elevated VL test, and clients were followed until censoring occurred at the time of a repeat VL measure, transfer, death, or administratively at the end of the observation period of 10 January 2022. In the first stage analysis, individuals were categorized into 1 of 9 mutually exclusive and exhaustive states based on their clinical status at each time point after cohort entry (i.e., first elevated VL test) (Fig 1A): (1) in care but no return visit since initial elevated VL (treatment failure), (2) 1 return visit with no repeat VL measure, (3) 2 return visits with no repeat VL measure, (4) 3+ return visits with no repeat VL measure, (5) treatment interruption, (6) repeat VL not suppressed (≥1,000 copies/ml), (7) repeat VL suppressed (<1,000 copies/ml), (8) documented transfer, and (9) death. We then applied nonparametric multistate analysis based on the Aalen–Johansen method that accounted for client movements between care states over time [19]. Individuals could transition between nonabsorbable states an unlimited number of times based on the Markov assumption (i.e., after entering a state, prior states do not impact subsequent outcomes) until they transitioned into an absorbable state (i.e., repeat VL measure, transfer, death) or were [24] censored (Fig 1) [19]. We estimated the probability of a client being in any particular state over time, and generated estimates for composite states such as any history of return visit, VL test rechecked, ever >60 days late. We also calculated median times with interquartile range (IQR) and instantaneous hazards of transition between states. We stratified analyses based on whether individuals were on TLE or TLD at the time of initial treatment failure to examine differences in care delivery and outcomes across these groups. We also conducted sensitivity analyses where we stratified by secular time periods relative to the COVID-19 pandemic. We used bootstrapping (1,000 iterations) to obtain 95% confidence intervals (CI).

#### Guideline concordant switch to second-line ART regimens (i.e., after two elevated VL ≥1,000 copies/mL).

Among individuals with two consecutive elevated VL tests, we repeated multistate analyses to assess the longitudinal experience of switching to second-line therapy (State 6 from first multistate analysis) in a second-stage analysis. To ensure regimen changes were due to treatment failure rather than programmatic roll out of TLD, this analysis was restricted to periods after individuals had two consecutive elevated VL tests. For the second stage analysis, time zero was the date of second consecutive elevated VL test, and clients were censored at the time of a suppressed repeat VL test (after already having at least two consecutive elevated VL tests), regimen switch to second-line, transfer, death, or administratively censored. Individuals were again categorized in mutually exclusive and exhaustive states over time (Fig 1B): (10) treatment failure, due for second-line switch with no return visit, (11) 1 attended visit with no switch made, (12) 2 attended visits with no switch made, or (13) 3+ attended visits with no switch made, (14) treatment interruption, (15) repeated VL test unsuppressed (≥1,000 copies/mL), (16) repeated VL test suppressed (<1,000 copies/mL), (17) switched to second-line regimen, (18) documented transfer, and (19) death. For this analysis, absorbable states included a suppressed repeat VL test (after already having at least two consecutive elevated VL tests), regimen switch to second-line, transfer, and death. A repeat unsuppressed VL test was considered nonabsorbent as individuals were still indicated for a regimen switch (Fig 1B). Analyses were stratified based on whether individuals were on TLE or TLD at the time of initial treatment failure and time periods. We used bootstrapping (1,000 iterations) to obtain 95% confidence intervals.

Among individuals who switched to second-line regimens after two consecutive elevated VL tests, an alluvial diagram was used to depict history of ART regimen changes and assess concordance of these changes with the national guidelines at the time of initial treatment failure, subsequent VL test, and at switch to second-line. ART regimens were categorized based on their main drug class (i.e., EFV, DTG, or LPVr) and NRTIn (nucleoside/nucleotide reverse transcriptase inhibitors) backbone (TDF/XTC compared to AZT/XTC). This analysis was restricted to clients who had an ART regimen recorded in the electronic health record.

### Predictors of VL monitoring and switch

We also used Cox proportional hazards models to examine associations between baseline sociodemographic and clinical characteristics and outcomes of interest: time to repeat VL test and re-suppression after initial treatment failure, as well as time to second-line switch or time to re-suppression prior to switch after two consecutive elevated VL tests (i.e., due for regimen switch). We accounted for within-facility correlation by including facility as a fixed effect. We used multiple imputation with chained equations (*n* = 20 imputations) to account for missingness in sex, age, WHO stage, marital, and education status. We use directed acyclic graphs based on a priori hypotheses of causal relationships to interpret results based on whether covariates should be considered confounders or mediators.

All analyses were conducted with R 4.3.1 software (R Foundation for Statistical Computing, Vienna, Austria) using the mstate package [14,15] and Stata MP 17.0 (Stata Corp LLC, College Station, Texas) [20].

## Results

### Client characteristics

**Fig 2:**
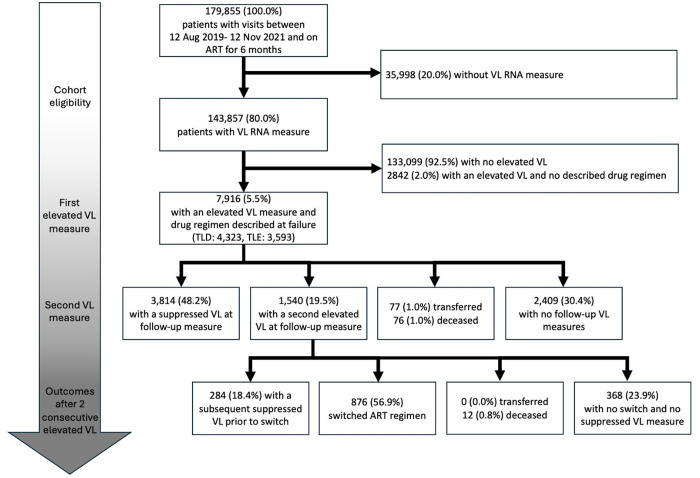
Flowchart of client inclusion and outcomes. The flowchart shows the selection of patients with an elevated VL from an initial cohort of 179,855 patients on ART for ≥6 months with visits between 12 Aug 2019 and 12 Nov 2021, outcomes following. We excluded 35,998 (20.0%) as they had no VL test, and 133,099 (92.5%) had no elevated VL in the electronic health record. We excluded 2,842 (2.0%) who had an elevated VL but no described drug regimen. We included a total of 7,916 (5.5%) in our analysis cohort with an initial elevated VL test and drug regimen described at failure. Of these, 48.2% achieved suppression, 19.5% had a second elevated VL test, 30.4% had no follow-up VL test, and 2% had either transferred or died. For those with two consecutive elevated VLs (*n* = 1,540), outcomes included subsequent suppression without switch (18.4%), regimen switch (56.9%), transfer or death (0.8%), or no further action (23.9%). Abbreviations: ART, antiretroviral therapy; VL, viral load.

Between 1 August 2019 and 31 November 2021, 179,885 clients accessed care at one of the 24 study facilities after being on ART for at least 180 days, and 143,857 (79.9%) had a VL test on record. Among those with a VL test, 10,758 (7.5%) individuals had a VL test ≥1,000 copies/ml and of those, 7,916 (73.6%) individuals had their first-line drug regimen documented at the time of treatment failure and were included in this analysis (Fig 2). Of these, 4,138 (52.3%) were female, median age was 36.7 years (IQR 29.9–43.6), and median time on ART at treatment failure was 3.3 years (IQR 1.7–6.6) (Table 1). Of those who had their first-line drug regimen documented, 4,323 (54.6%) were on TLD and 3,593 (45.4%) on TLE, at the time of the first elevated VL test (Table 1, S1 Fig). Those on TLD were less likely to be female and more likely to have been on ART for less than 2 years, but other baseline characteristics were similar compared to those on TLE.

**Table 1:** Baseline characteristics of clients with an elevated viral load.

		Total (*N* = 7,916)	TLD (*n* = 4,323)	TLE (*n* = 3,593)
Gender	Female	4,138 (52.3%)	1,943 (44.9%)	2,195 (61.1%)
	Male	3,155 (39.9%)	2,105 (48.7%)	1,050 (29.2%)
	Missing	623 (7.9%)	275 (6.4%)	348 (9.7%)
Age category	18–24	844 (10.7%)	421 (9.7%)	423 (11.8%)
	25–34	2,287 (28.9%)	1,220 (28.2%)	1,067 (29.7%)
	35–44	2,655 (33.5%)	1,440 (33.3%)	1,215 (33.8%)
	45–54	1,131 (14.3%)	708 (16.4%)	423 (11.8%)
	55+	374 (4.7%)	258 (6.0%)	116 (3.2%)
	Missing	625 (7.9%)	276 (6.4%)	349 (9.7%)
Age, median (IQR)		36.7 (29.9–43.6)	37.3 (30.5–44.6)	35.9 (29.3–42.2)
Marital status	Single	1,108 (14.0%)	604 (14.0%)	504 (14.0%)
	Married	3,718 (47.0%)	2,078 (48.1%)	1,640 (45.6%)
	Divorced	883 (11.2%)	507 (11.7%)	376 (10.5%)
	Widowed	471 (5.9%)	270 (6.2%)	201 (5.6%)
	Missing	1,736 (21.9%)	864 (20.0%)	872 (24.3%)
Education	None	380 (4.8%)	172 (4.0%)	208 (5.8%)
	Primary	1,852 (23.4%)	1,002 (23.2%)	850 (23.7%)
	Secondary	3,695 (46.7%)	2,095 (48.5%)	1,600 (44.5%)
	University	390 (4.9%)	260 (6.0%)	130 (3.6%)
	Missing	1,599 (20.2%)	794 (18.4%)	805 (22.4%)
WHO stage	1	3,528 (44.6%)	1,990 (46.0%)	1,538 (42.8%)
	2	1,028 (13.0%)	576 (13.3%)	452 (12.6%)
	3	1,355 (17.1%)	712 (16.5%)	643 (17.9%)
	4	82 (1.0%)	48 (1.1%)	34 (0.9%)
	Missing	1,923 (24.3%)	997 (23.1%)	926 (25.8%)
ART regimen at first elevated VL test	TLD	4,323 (54.6%)	4,323 (100.0%)	0 (0.0%)
	TLE	3,593 (45.4%)	0 (0.0%)	3,593 (100.0%)
Year of ART initiation	2004–2010	915 (11.6%)	464 (10.7%)	451 (12.6%)
	2011–2016	2,788 (35.2%)	1,320 (30.5%)	1,468 (40.9%)
	2017–2018	2,505 (31.6%)	1,159 (26.8%)	1,346 (37.5%)
	2019–2021	1,708 (21.6%)	1,380 (31.9%)	328 (9.1%)
Time on ART at first elevated VL test	<1 year	1,015 (12.8%)	732 (16.9%)	283 (7.9%)
	1–2 years	1,446 (18.3%)	757 (17.5%)	689 (19.2%)
	2–5 years	2,603 (32.9%)	1,344 (31.1%)	1,259 (35.0%)
	5–10 years	2,036 (25.7%)	1,034 (23.9%)	1,002 (27.9%)
	10+ years	816 (10.3%)	456 (10.5%)	360 (10.0%)
Appointment interval at time of first elevated VL test	30 days	1,021 (12.9%)	334 (7.7%)	687 (19.1%)
	60 days	128 (1.6%)	56 (1.3%)	72 (2.0%)
	90 days	3,409 (43.1%)	1,695 (39.2%)	1,714 (47.7%)
	120 days	1,050 (13.3%)	519 (12.0%)	531 (14.8%)
	150 days	303 (3.8%)	211 (4.9%)	92 (2.6%)
	180 days	2,005 (25.3%)	1,508 (34.9%)	497 (13.8%)
Time between first and second VL test	30–180 days	2,675 (33.8%)	1,315 (30.4%)	1,360 (37.9%)
	180–365 days	2,209 (27.9%)	1,069 (24.7%)	1,140 (31.7%)
	1–2 years	466 (5.9%)	175 (4.0%)	291 (8.1%)
	>2 years	4 (0.1%)	1 (0.0%)	3 (0.1%)
	Missing	2,562 (32.4%)	1,763 (40.8%)	799 (22.2%)
Facility size	Small Health Center	247 (3.1%)	154 (3.6%)	93 (2.6%)
	Medium Health Center	1,782 (22.5%)	926 (21.4%)	856 (23.8%)
	Large Health Center	2,057 (26.0%)	1,149 (26.6%)	908 (25.3%)
	Hospital	3,830 (48.4%)	2,094 (48.4%)	1,736 (48.3%)

Abbreviations: ART, antiretroviral therapy; IQR, interquartile range; TLD, tenofovir disoproxil fumarate/lamivudine or emtricitabine/dolutegravir TDF/XTC/DTG; TLE, tenofovir disoproxil fumarate/lamivudine or emtricitabine/efavirenz TDF/XTC/EFV; VL, viral load.

Small Health Center: <2,500 clients; Medium Health Center: 2,500−7,500 clients; Large Health Center: 7,500−20,000 clients.

### Outcomes after first elevated viral load among clients due for EAC and repeat viral load in 3–6 months

**Fig 3:**
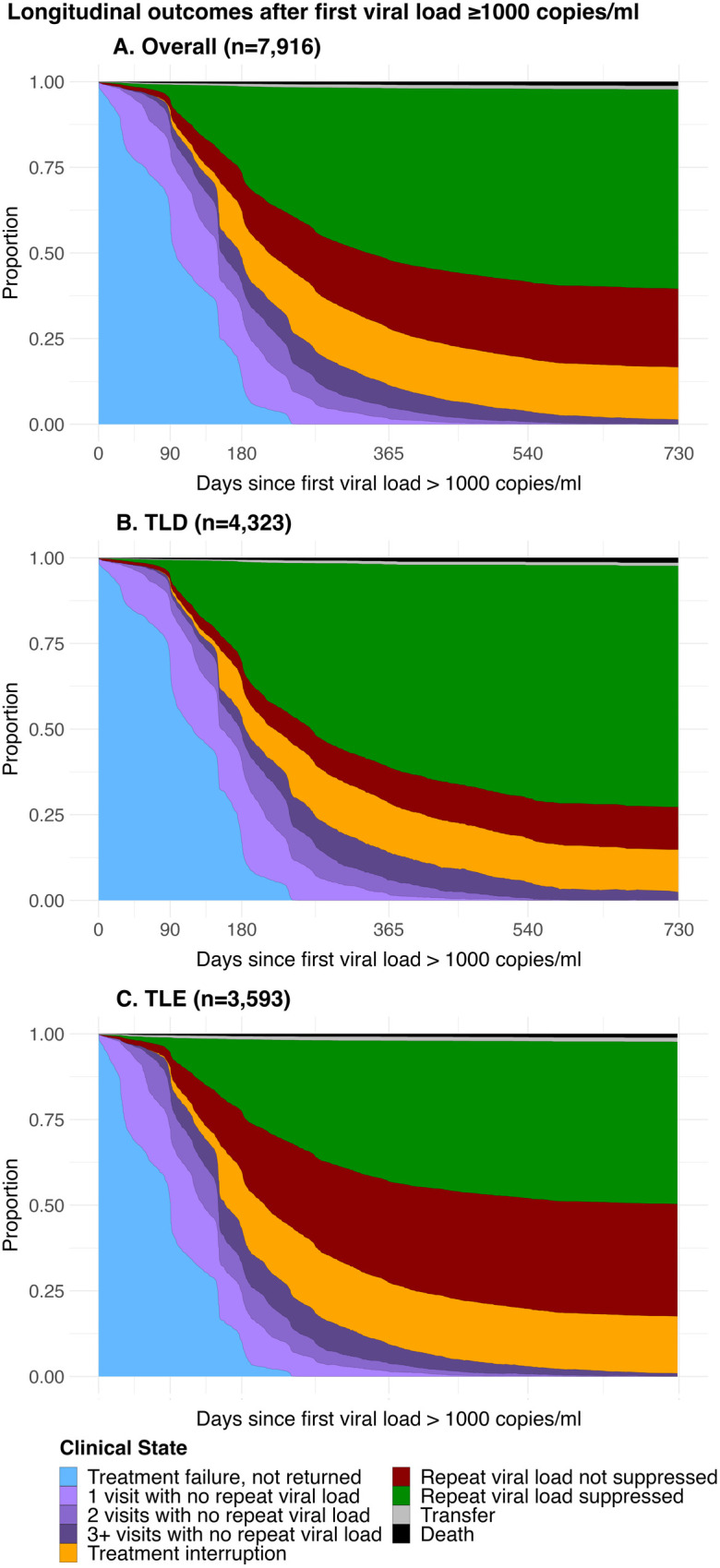
Longitudinal outcomes after first VL ≥1,000 copies/ml. This figure shows the proportion of clients estimated to be in each state at any given time after an individual’s first elevated VL, accounting for the transitions made between different states over time. **(A)** Outcomes following a first VL ≥1,000 copies/ml for all regimens (*n* = 7,916); **(B)** Outcomes following a first VL ≥1,000 copies/ml, regimen at cohort entry TDF/XTC/DTG (TLD) (*n* = 4,323); **(C)** Outcomes following a first VL ≥1,000 copies/ml, regimen at cohort entry TDF/XTC/EFV (TLE) (*n* = 3,593). Abbreviations: TLD, tenofovir disoproxil fumarate/lamivudine or emtricitabine/dolutegravir TDF/XTC/DTG; TLE, tenofovir disoproxil fumarate/lamivudine or emtricitabine/efavirenz TDF/XTC/EFV; VL, viral load.

Thirty-seven percent (37.0%, CI 36.2, 37.8%) and 72.2% (CI 71.3, 73.0%) had returned to clinic for at least 1 visit (including visits with and without a VL test) by 3- and 6-months (Figs 3A and S2 and S1 and S2 Tables), respectively, after the initial elevated VL test (median time to return 92 days IQR 42–168 among returners). At 6- and 12-months, 36.4% (CI 35.5, 37.2%) and 70.1% (CI 69.3, 70.9%) in the overall cohort had a repeat VL test (Figs 3A and S2 and S2 Table). Among those who had returned for at least 1 visit, 49.9% (CI 48.9, 51.0%) and 78.4% (CI 77.6, 79.3%) had a VL test repeated at 6- and 12-months, respectively. Of those with a repeat VL test, median overall time from initial VL test to repeat VL test was 180 days (IQR 117–254), and median time from returning to clinic to having VL test repeated was 63 days (IQR 0–132). At 12 months, 16.6% (CI 15.9, 17.2%) of individuals were currently experiencing a treatment interruption, 32.5% (CI 31.8, 33.3%) had ever experienced a treatment interruption since treatment failure, and 11.4% (CI 10.3, 12.4%) had return visits but no repeat VL test. Additionally, 59.3% (CI 57.5, 61.0%) of those who did not have a VL rechecked by 1 year were in a treatment interruption (Figs 3A and S2 and S2 Table). Results were similar when stratified by COVID-19 period (S3 Table).

In the overall cohort, 50.1% (CI 48.9, 51.3%) of individuals had documented re-suppression at 12 months. At 6 months, 29.2% (CI 28.1, 30.3%) of individuals on TLD and 21.0% (CI 20.0, 22.0%) on TLE had documented re-suppression, increasing to 59.2% (CI 57.9, 60.4%) on TLD and 41.1% (CI 40.0, 42.2%) on TLE at 12 months. Among those who had a VL test repeated by 12 months, 85.0% (CI 83.9, 86.1%) on TLD resuppressed compared to 58.2% (CI 56.8, 59.8%) on TLE (risk difference 26.8% CI 25.0, 28.6%) (Figs 3B, 3C, and S2 and S2 Table).

### Outcomes after two consecutive elevated viral loads among clients due for switch

**Fig 4:**
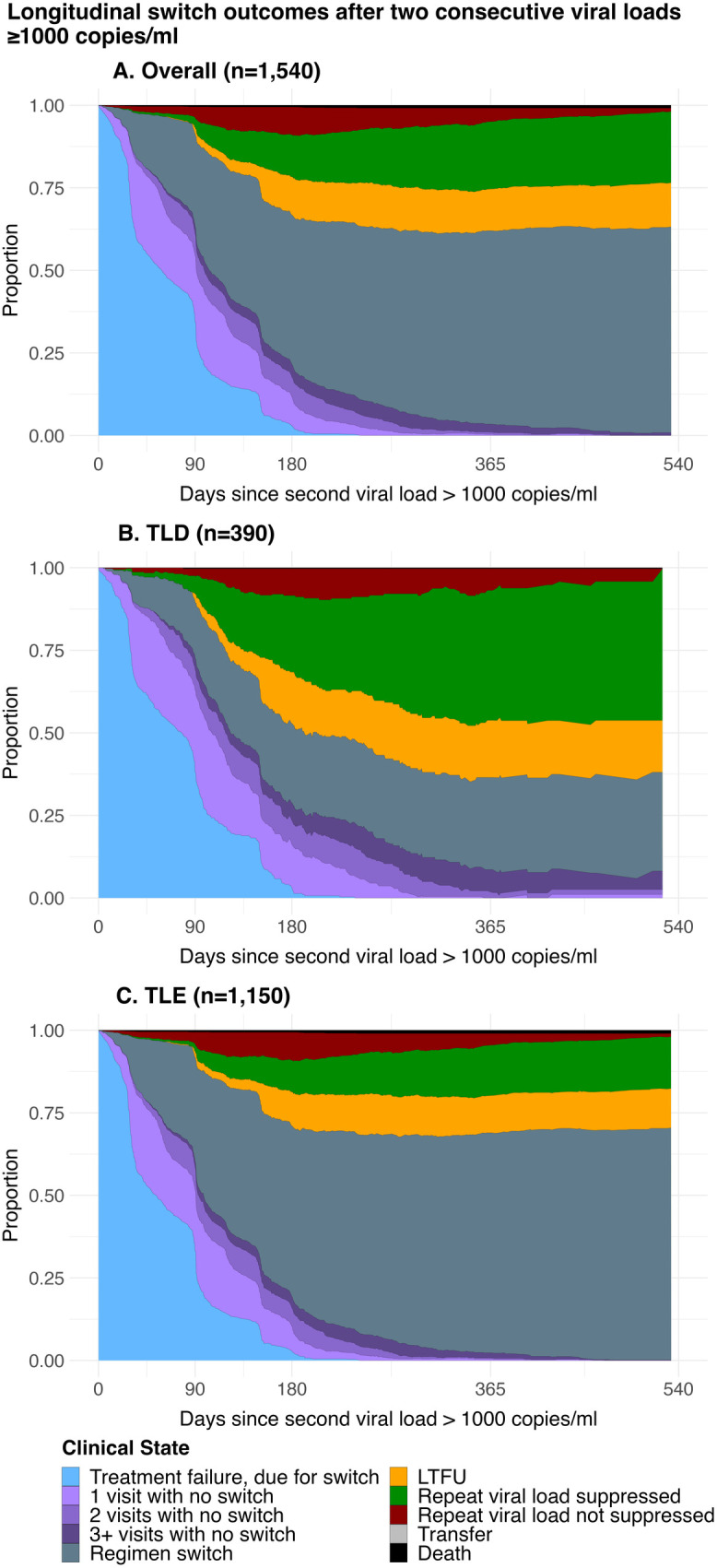
Longitudinal ART regimen switch outcomes after two consecutive elevated VL ≥1,000 copies/ml, TLD vs. TLE. This figure shows the proportion of clients estimated to be in each state at any given time after an individual is due for a regimen switch (i.e., two consecutive elevated VLs), accounting for the transitions made between different states over time. **(A)** Outcomes following two VL ≥1,000 copies/ml (*n* = 1,540). **(B)** Outcomes following two VL ≥1,000 copies/ml, regimen at cohort entry TDF/XTC/DTG (TLD) (*n* = 390); **(C)** Outcomes following two VL ≥1,000 copies/ml, regimen at cohort entry TDF/XTC/EFV (TLE) (*n* = 1,150). Abbreviations: ART, antiretroviral therapy; TLD, tenofovir disoproxil fumarate/lamivudine or emtricitabine/dolutegravir TDF/XTC/DTG; TLE, tenofovir disoproxil fumarate/lamivudine or emtricitabine/efavirenz TDF/XTC/EFV; VL, viral load.

A total of 1,540 individuals had a subsequent elevated VL test and were due for a switch to second-line regimen per national ART guidelines (889 [57.7%] female; median age 35.5 [IQR 28.6–42.1]), (390 [25.3%] on TLD and 1,150 [74.7%] on TLE) (S4 Table). After a second elevated VL test and being due for switch, 63.3% (CI 61.5, 65.2%) and 89.2% (CI 87.9, 90.6%) had returned to clinic for at least 1 visit by 3- and 6-months, respectively (median time to return 49 days IQR 29–92 among returners) (Figs 4A and S3 and S5 Table). Among those on TLD, at 6 months, 24.0% (CI 20.5, 27.5%) were switched and 33.2% (CI 29.4, 37.3%) had another VL test rechecked for a third time prior to switch (68.0% CI 61.6, 75.2% suppressed among those with repeated VL), going up to 27.9% (CI 24.1, 31.5%) switched and 52.4% (CI 47.2, 57.4%) had another VL test rechecked (70.0% CI 64.1, 76.6% suppressed) at 12-months (Fig 4B and 4C and S5 Table). In contrast, among those on TLE, 51.7% (CI 49.5,54.2%) were switched and 22.6% (CI 20.9, 24.6%) had a VL test rechecked prior to switch (40.7% CI 36.1, 45.4 suppressed) at 6-months, and that increased to 66.6% (CI 64.5, 68.9%) switched and 32.1% (CI 29.9, 34.1%) had a VL test rechecked (42.9% CI 39.1, 47.0% suppressed) by 12-months. Overall, at 12 months, 38.7% (CI 34.5, 43.6%) fewer individuals on TLD had been switched compared to those on TLE, but 24.1% (CI 19.1, 29.4%) more had resuppressed prior to switch (Figs 4A and S3 and S5 Table). Lastly, 13.2% (CI 11.7, 14.5%) of individuals were currently experiencing treatment interruption and 23.4% (CI 21.6, 24.8%) had ever had treatment interruptions prior to switch or re-suppression at 12 months in the overall cohort.

### Trajectories of ART regimens

**Fig 5:**
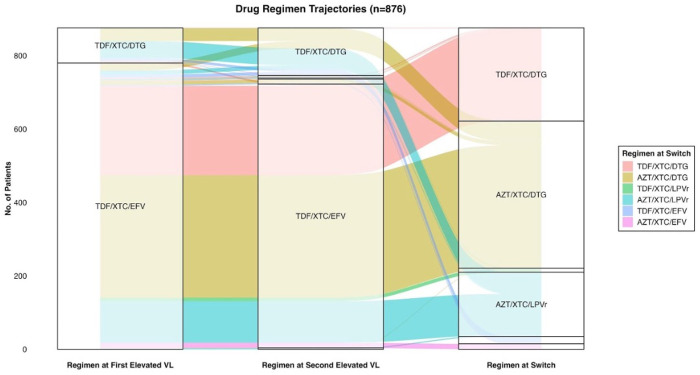
Drug at regimen switch trajectories. This figure illustrates the common regimen transitions among 876 patients from the time of the first elevated VL to the second elevated VL and eventual switch. Those on TDF/XTC/DTG most frequently switched to TDF/XTC/DTG and AZT/XTC/DTG. Those on TDF/XTC/EFV had diverse switches with most being switched to AZT/XTC/DTG and TDF/XTC/DTG, with a smaller proportion being switched to AZT/XTC/LPVr. Abbreviations: AZT, Azidothymidine; DTG, dolutegravir; EFV, efavirenz; LPVr, ritonavir-boosted lopinavir; TDF, tenofovir disoproxil fumarate; VL, viral load; XTC, lamivudine or emtricitabine.

876 individuals were switched to second-line regimen after at least two consecutive elevated VL tests (581 [66.3%] female; median age 35.9 [IQR 29.2–42.1]), (96 [11.0%] on TLD and 780 [89.0%] on TLE) (Fig 5). Median time from initial treatment failure and from second elevated VL test to switch was 273 days (IQR 189–379) and 91 days (IQR 39–167), respectively. Among those on TLD at time of treatment failure, 46 (47.9%) switched to AZT/XTC/LPVr, 35 (36.5%) only had TDF changed to AZT (i.e., switched to AZT/XTC/DTG), and 8 (8.3%) were transitioned to an NNRTI-based regimen. Among those on TLE, 366 (46.9%) switched to AZT/XTC/DTG, 248 (31.8%) to TDF/XTC/DTG, and 139 (17.8%) to AZT/XTC/LPVr. Only 14 (1.8%) had a TDF switch to AZT. At 6-months after switch, 54.3% (CI 51.5, 57.2%) had a VL test rechecked overall and this increased to 80.7% (CI 78.3, 83.3%) by 1-year; 83.0% (CI 80.7, 85.3%) of those who had VL test rechecked by 1 year were suppressed (75.4% [CI 66.6, 84.8%] on TLD first-line versus 83.7% [CI 81.4, 86.0%] on TLE first-line).

### Predictors of viral load monitoring and switch

In multivariable Cox proportional hazards analyses, being scheduled for a longer appointment interval at the time initial elevated VL test was drawn was highly associated with longer time to return (aHR 0.45 [CI: 0.42, 0.49, *p* < 0.001] for 3 m and aHR 0.25 [CI: 0.23, 0.28, *p* < 0.001] for 6 m compared to 1 m interval), but association was less strong for having a VL test checked (aHR 0.85 [CI: 0.76, 0.94, *p* < 0.001] for 6 m) and time to documented re-suppression (aHR 0.89 [CI: 0.79, 1.01, *p* < 0.001] for 6 m). Longer appointment intervals at time of second unsuppressed VL test were also associated with longer times to switch to second-line regimens (aHR 0.36 [CI: 0.21, 0.61, *p* < 0.001] for 6 m versus 1 m interval) (S6 Table).

Being on TLD was not associated with time to VL test check or return, but was highly associated with time to documented re-suppression (aHR 1.42 [1.31, 1.54, *p* < 0.001]) after initial treatment failure. After two elevated VLs, individuals on TLD were much less likely to be switched (aHR 0.37 [CI: 0.29, 0.47, *p* < 0.001]) and were more likely to have another VL test checked and suppressed (S6 Table).

Additionally, being female, older age, and a longer time on ART were associated with faster time to return, time to rechecking VL test, and time to a documented suppressed VL test. Later time periods and being at medium or larger-sized facilities were also associated with time to VL test check and suppression (S6 Table).

## Discussion

Among individuals with treatment failure, we identified substantial delays and missed opportunities in rechecking VL test status and switching to second-line therapy in routine care in Zambia. Only 36.4% of individuals had a VL test rechecked by 6 months after initial treatment failure, driven by delays in returning to clinic, treatment interruptions, and missed opportunities to do a VL test after return. At 1-year, only 50.1% had documented re-suppression. However, rates of re-suppression were high among those who had a VL test rechecked (70.1% overall), and even higher for those on TLD (85.0%). Similar patterns occurred after confirmation of failure based on two consecutive elevated VL tests, with fewer than 25% switching within 6 months of initial treatment failure (upper limit of guideline recommendations). Notably, individuals on TLD were far more likely to resuppress (85.0% versus 58.2% on TLE), despite similar care patterns and timing of VL test rechecks. Providers were also more reluctant to switch those on TLD to second-line regimens. These findings reflect real-world outcomes under routine programmatic settings and underscore the critical care gaps driven by both client and health system barriers that impede timely delivery of recommended care.

First, many clients did not return promptly, which likely delayed both the initiation of EAC and timely assessment of response. By 3 months, only 37% had returned to the clinic, and only 7% had a VL test rechecked. The length of the scheduled appointment interval at the time of elevated VL was highly associated with longer time to return, suggesting the health system did not adjust care delivery to account for the urgency of treatment failure. While shifting towards longer ART dispensations has helped reduce clinic visit burden on clients [21–24], it complicates timely response to viremia when clients do not return until their next refill is due [25]. Results are not immediately available and providers are not able to act in real time, and this ultimately requires responsive systems that can adapt once VL results are available. This includes formalized procedures for laboratory notification of providers, proactive outreach to clients, timely EAC initiation in manner that accommodates individual needs, followed by close follow-up and reassessing response to ART. Innovations like telehealth could help initiate prompt adherence counseling prior to individuals physically returning for their next clinical visit. Point-of-care VL testing has promise for enabling immediate action [26], but such approaches must still be complemented by systems that ensure continued follow-up of clients [27–29].

Second, treatment interruptions are common and represent a major barrier to rechecking VL tests and subsequent outcomes. In our study, approximately 32.5% of individuals were ≥60 days late for an appointment at least once after treatment failure, and 59.3% of those who did not have a VL rechecked by 1 year were in a treatment interruption. Similar gaps occurred after individuals were due for switch. As programmes mature, treatment interruptions remain one of the largest drivers of viremia in the community, but this major challenge often gets obscured behind metrics that only consider those in whom a VL test is documented [30]. While re-suppression was high among those who were retested, it was substantially lower when also accounting for those with treatment interruptions. A complex interplay of structural, clinic-based, and psychosocial factors undermines retention and access to medications [31]. Judgmental and antagonistic interactions with providers may be especially heightened for individuals experiencing adherence challenges [32–35]. Person-centered approaches that support providers in delivering nonjudgmental, tailored counseling should be considered central to strategies aimed at improving outcomes after treatment failure [36,37].

Third, when clients did return to care, implementation gaps persisted at the health system level. Providers often missed opportunities to recheck a VL test or switch to second-line therapy despite clients being due at these visits. For example, 40.8% of those who had not had a VL test rechecked by 1 year were still in care. Delays may be driven by rigid insistence on completing all three EAC sessions, provider perception that clients were not yet adherent, or simple lack of familiarity with treatment failure guidelines [32,38]. Addressing these issues requires system-level interventions to ensure that each health interaction is fully taken advantage of to support optimal care. This requires health system strategies that strengthen organizational culture, promote team-level accountability, ensure provider coherence [39], and clear communication on managing treatment failure. Flexible interpretation of guidelines, such as not delaying rechecking VL tests while waiting for all EAC sessions may help improve responsiveness [40].

Our findings also provide additional insights as HIV programmes shift from TLE to TLD. In this study, a high number of individuals resuppressed while maintaining TLD. Despite similar patterns of care, the prevalence of re-suppression was 25% higher in individuals on TLD compared to TLE among those who had a VL checked [3,5,41,42]. Assuming comparable adherence, this difference likely represents the gap in acquired resistance that frequently occurs on TLE but rarely on TLD. Providers were also much less likely to switch clients on TLD, often rechecking VLs multiple times instead even when due. Prior studies have demonstrated very low levels of DTG resistance after treatment failure on TLD [4,43,44] to date, compared to fairly high levels after treatment failure on TLE [45]. The effectiveness and forgiving nature of DTG-based regimens has shifted focus away from resistance concerns and switching to second-line regimens [4,8,41,42] and towards adherence counseling as a primary focus. Nonetheless, there are emerging concerns that DTG resistance will continue to grow, and an evidence-based approach to treatment failure on TLD is needed [46,47]. In our study, 45% of those who had switched regimens on TLD were likely switched to an inappropriate regimen (i.e., 37% only had TDF changed to AZT, 8% switched to NNRTI-based regimen), although it is also worth noting that a small proportion of individuals on TLD required regimen switches and that formal guidance on use of integrase-based second-line regimens only after TLE failure were not issued until after the study period in 2022. Results from NADIA and D2EFT suggest that maintaining TLD or switching to DTG plus PI may be appropriate strategies, although these studies were in individuals failing TLE and not TLD [48–50]. REVAMP demonstrated little benefit of genotypic assays to identify resistance at treatment failure on TLE despite 68% with detectable resistance, and any clinical benefit is likely even further diminished with low rates of resistance on TLD (although it may still be cost-effective given cost of PIs) [51,52]. Evidence on adapting approaches to treatment failure, including monitoring for suppression, when to consider switching regimens, and what to switch to, are needed with widespread roll out of TLD as first-line regimens.

There are several limitations to this study. First, Electronic Health Record (EHR) data did not include whether outreach activities were performed, whether EAC was delivered, or provider rationale (if any) to delay VL. Nevertheless, this analysis highlights the major delays in the timeliness and success of current approaches to treatment failure and identifies gaps that can be further characterized in future studies. Second, there may be incomplete EHR data capture, although prior analyses have demonstrated high-quality data [53]. Third, we could not link visits to individual providers, limiting assessment of provider-specific practices. Fourth, our analysis only included individuals with documented treatment failure, likely missing individuals with viremia that goes undocumented due to not being in care or missing VL measures [30]. Fifth, TLD was rolled out during the study period, and some individuals may have switched to TLD due to this programmatic scale-up rather than to manage treatment failure on TLE. Although this would likely improve re-suppression rates after TLE failure, we still noted marked differences in re-suppression on TLD versus TLE, and we would better capture current fidelity to guideline-based care algorithms by categorizing regimen switches only after two consecutive elevated VLs. Sixth, potential selection bias may exist, as individuals on TLD are less likely to experience treatment failure to begin with. Lastly, we could not verify whether clients with treatment interruptions had transferred to other sites, although prior studies show long delays in ART reinitiation among many of those transferring without official documentation [54].

In summary, there are substantial gaps along the treatment failure cascade precluding viral re-suppression, driven by delays in outreach, return for adherence counseling, and repeat VL monitoring to assess response; treatment interruptions; and missed opportunities in rechecking VL test status or switching to second-line therapy in routine care in Zambia. These gaps stem from both client-related and health system inefficiencies and increasing shifts to six-month ART distribution, underscoring the need for systemic health system approaches to reengage individuals with treatment failure promptly. Furthermore, re-suppression rates were significantly higher among individuals who failed TLD compared to TLE, and the high re-suppression rates on TLD suggest that an increasingly lower number of treatment failures are driven by resistance, and switching may not always be necessary. Although these findings are from Zambia, they remain broadly relevant for public sector HIV programs with similar implementation challenges. In the modern era of ART with TLD used as first-line regimens, it is critical to reimagine approaches to treatment failure and create formal and explicit guidance to prioritize person-centered strategies for timely engagement and support for client retention and adherence and likely less emphasis of resistance concerns and switches to second-line. Future efforts should prioritize understanding how to best advance approaches to treatment failure that align with the evolving needs of the TLD era and integrate person-centered approaches to target the particular care gaps that exist along the treatment failure cascade.

## Supporting information

S1 Table Absolute frequency of specific transitions. (DOCX)

S2 Table Proportion in individual and composite states after initial elevated VL. (DOCX)

S3 Table Multistate results stratified by COVID-19 time-period and regimen. (DOCX)

S4 Table Characteristics of those with a second elevated viral load. (DOCX)

S5 Table Proportion in individual and composite states after two elevated VLs and due for switch. (DOCX)

S6 Table Cox proportional hazards model for predictors of return, VL check and suppress after first VL elevated. (DOCX)

S1 Fig Distribution of individuals with elevated viral on TLE versus TLD over time. (DOCX)

S2 Fig Transition hazards between states over time after initial elevated VL. (DOCX)

S3 Fig Transition hazards between states over time after due for switch. (DOCX)

S1 STROBE Checklist This checklist is licensed under the Creative Commons Attribution 4.0 International License (CC BY 4.0; https://creativecommons.org/licenses/by/4.0/).
(DOCX)

## Abbreviations

ART: antiretroviral therapy
AZT: zidovudine
D2EFT,: Dolutegravir and Darunavir evaluation in adults failing therapy;
DTG: dolutegravir
EAC: enhanced adherence counseling
EFV: efavirenz
EHR,: electronic health record;
IQR: interquartile range
LPVr: lopinavir
MOH: Ministry of Health
NADIA,: nucleosides and Darunavir/Dolutegravir in Africa;
NNRTI: non-nucleoside reverse transcriptase inhibitor
NRTI: nucleoside/nucleotide reverse transcriptase inhibitors
PI: protease inhibitor
REVAMP: resistance, evaluation, and management in HIV prevention trials
STROBE: Strengthening the Reporting of Observational Studies in Epidemiology
TDF: tenofovir disoproxil fumarate
TDF/XTC/DTG (TLD): tenofovir disoproxil fumarate/lamivudine or emtricitabine/dolutegravir
TDF/XTC/EFV (TLE): tenofovir disoproxil fumarate/lamivudine or emtricitabine/efavirenz
VL: viral load
WHO: World Health Organization
XTC: lamivudine or emtricitabine
