# Supplementary material for: Identifying care gaps along the HIV treatment failure cascade: A multistate analysis of viral load monitoring, re-suppression, and regimen switches in Zambia
Source: PLoS Med. 2025 Sep 3;22(9):e1004720. doi: 10.1371/journal.pmed.1004720 (PMC12422583; doi:10.1371/journal.pmed.1004720)
Supplement: S1 Table — (DOCX) [file pmed.1004720.s001.docx]

**S1 Table. Absolute Frequency of Specific Transitions**

| **Possible transitions after one elevated VL (**≥**1000 copies/mL) - Overall** | | | | | | | | | | |
| --- | --- | --- | --- | --- | --- | --- | --- | --- | --- | --- |
| **State** | | **State transitioned to** | | | | | | | | |
|  |  | **1 visit with no repeat VL**  **(2)** | **2 visits with no repeat VL**  **(3)** | **3+ visits with no repeat VL**  **(4)** | **Treatment interruption**  **(5)** | **Repeat VL not suppressed**  **(6)** | **Repeat VL suppressed**  **(7)** |  | **Transfer**  **(8)** | **Death**  **(9)** |
| **State transitioned from** | **Treatment failure, not returned to care (1)** | 4,506 | - | - | 1,427 | 423 | 1,030 |  | 15 | 24 |
|  | **1 visit with no repeat VL** **(2)** | - | 2,753 | - | 611 | 356 | 948 |  | 34 | 16 |
|  | **2 visits with no repeat VL** **(3)** | - | - | 1,511 | 307 | 271 | 639 |  | 16 | 5 |
|  | **3+ visits with no repeat VL** **(4)** | - | - | - | 250 | 242 | 822 |  | 12 | 15 |
|  | **Treatment interruption** **(5)** | 498 | 200 | 179 | - | 248 | 375 |  | 0 | 16 |
| **Possible transitions after one elevated VL (**≥**1000 copies/mL) - TLD** | | | | | | | | | | |
| **State** | | **State transitioned to** | | | | | | | | |
|  |  | **1 visit with no repeat VL**  **(2)** | **2 visits with no repeat VL**  **(3)** | **3+ visits with no repeat VL**  **(4)** | **Treatment interruption**  **(5)** | **Repeat VL not suppressed**  **(6)** | **Repeat VL suppressed**  **(7)** |  | **Transfer**  **(8)** | **Death**  **(9)** |
| **State transitioned from** | **Treatment failure, not returned to care (1)** | 2,297 | - | - | 662 | 165 | 703 |  | 7 | 13 |
|  | **1 visit with no repeat VL** **(2)** | - | 1,296 | - | 270 | 77 | 593 |  | 15 | 9 |
|  | **2 visits with no repeat VL** **(3)** | - | - | 662 | 133 | 57 | 344 |  | 7 | 3 |
|  | **3+ visits with no repeat VL** **(4)** | - | - | - | 75 | 32 | 336 |  | 12 | 7 |
|  | **Treatment interruption** **(5)** | 235 | 88 | 71 | - | 59 | 194 |  | 0 | 8 |
| **Possible transitions after one elevated VL (**≥**1000 copies/mL) - TLE** | | | | | | | | | | |
| **State** | | **State transitioned to** | | | | | | | | |
|  |  | **1 visit with no repeat VL**  **(2)** | **2 visits with no repeat VL**  **(3)** | **3+ visits with no repeat VL**  **(4)** | **Treatment interruption**  **(5)** | **Repeat VL not suppressed**  **(6)** | **Repeat VL suppressed**  **(7)** |  | **Transfer**  **(8)** | **Death**  **(9)** |
| **State transitioned from** | **Treatment failure, not returned to care (1)** | 2,209 | - | - | 765 | 258 | 327 |  | 8 | 11 |
|  | **1 visit with no repeat VL** **(2)** | - | 1,457 | - | 341 | 279 | 355 |  | 19 | 7 |
|  | **2 visits with no repeat VL** **(3)** | - | - | 849 | 174 | 214 | 295 |  | 9 | 2 |
|  | **3+ visits with no repeat VL** **(4)** | - | - | - | 175 | 210 | 486 |  | 8 | 8 |
|  | **Treatment interruption** **(5)** | 263 | 112 | 108 | - | 189 | 181 |  | 0 | 8 |
|  | | | | | | | | | | |
| **Possible transitions after two elevated VLs (**≥**1000 copies/mL) - Overall** | | | | | | | | | | |
| **State** | | **State transitioned to** | | | | | | | | |
|  |  | **1 visit with no switch or repeat**  **(11)** | **2 visits with no switch or repeat**  **(12)** | **3+ visits with no switch or repeat**  **(13)** | **Treatment interruption**  **(14)** | **Repeat VL not suppressed before switch**  **(15)** | **Repeat VL suppressed before switch**  **(16)** | **Regimen switch to second line**  **(17)** | **Transfer**  **(18)** | **Death**  **(19)** |
| **State transitioned from** | **Treatment failure, due for second line switch (10)** | 875 | - | - | 190 | 0 | 0 | 418 | 0 | 6 |
|  | **1 visit with no switch (11)** | - | 444 | - | 99 | 112 | 101 | 165 | 0 | 2 |
|  | **2 visits with no switch (12)** | - | - | 230 | 30 | 68 | 66 | 49 | 0 | 0 |
|  | **3+ visits with no switch (13)** | - | - | - | 14 | 70 | 92 | 33 | 0 | 1 |
|  | **Treatment interruption (14)** | 78 | 30 | 18 | - | 10 | 0 | 58 | 0 | 2 |
|  | **Repeat VL not suppressed before switch (15)** | - | - | - | 43 | - | 25 | 153 | 0 | 1 |
| **Possible transitions after two elevated VLs (**≥**1000 copies/mL) - TLD** | | | | | | | | | | |
| **State** | | **State transitioned to** | | | | | | | | |
|  |  | **1 visit with no switch or repeat**  **(11)** | **2 visits with no switch or repeat**  **(12)** | **3+ visits with no switch or repeat**  **(13)** | **Treatment interruption**  **(14)** | **Repeat VL not suppressed before switch**  **(15)** | **Repeat VL suppressed before switch**  **(16)** | **Regimen switch to second line**  **(17)** | **Transfer**  **(18)** | **Death**  **(19)** |
| **State transitioned from** | **Treatment failure, due for second line switch (10)** | 243 | - | - | 52 | 0 | 0 | 58 | 0 | 1 |
|  | **1 visit with no switch (11)** | - | 144 | - | 27 | 14 | 40 | 17 | 0 | 0 |
|  | **2 visits with no switch (12)** | - | - | 74 | 5 | 18 | 30 | 2 | 0 | 0 |
|  | **3+ visits with no switch (13)** | - | - | - | 4 | 13 | 35 | 4 | 0 | 0 |
|  | **Treatment interruption (14)** | 23 | 9 | 5 | - | 2 | 0 | 4 | 0 | 0 |
|  | **Repeat VL not suppressed before switch (15)** | - | - | - | 8 | - | 9 | 11 | 0 | 0 |
| **Possible transitions after two elevated VLs (**≥**1000 copies/mL) - TLE** | | | | | | | | | | |
| **State** | | **State transitioned to** | | | | | | | | |
|  |  | **1 visit with no switch or repeat**  **(11)** | **2 visits with no switch or repeat**  **(12)** | **3+ visits with no switch or repeat**  **(13)** | **Treatment interruption**  **(14)** | **Repeat VL not suppressed before switch**  **(15)** | **Repeat VL suppressed before switch**  **(16)** | **Regimen switch to second line**  **(17)** | **Transfer**  **(18)** | **Death**  **(19)** |
| **State transitioned from** | **Treatment failure, due for second line switch (10)** | 632 | - | - | 138 | 0 | 0 | 360 | 0 | 5 |
|  | **1 visit with no switch (11)** | - | 300 | - | 72 | 98 | 61 | 148 | 0 | 2 |
|  | **2 visits with no switch (12)** | - | - | 156 | 25 | 50 | 36 | 47 | 0 | 0 |
|  | **3+ visits with no switch (13)** | - | - | - | 10 | 57 | 57 | 29 | 0 | 1 |
|  | **Treatment interruption (14)** | 55 | 21 | 13 | - | 8 | 0 | 54 | 0 | 2 |
|  | **Repeat VL not suppressed before switch (15)** | - | - | - | 35 | - | 16 | 142 | 0 | 1 |

“-” represents a transition that is not possible

Abbreviations: VL, Viral Load; TLD, tenofovir disoproxil fumarate/lamivudine or emtricitabine/dolutegravir [TDF/XTC/DTG]; TLE, tenofovir disoproxil fumarate/lamivudine or emtricitabine/efavirenz [TDF/XTC/EFV]
