# Supplementary material for: Identifying care gaps along the HIV treatment failure cascade: A multistate analysis of viral load monitoring, re-suppression, and regimen switches in Zambia
Source: PLoS Med. 2025 Sep 3;22(9):e1004720. doi: 10.1371/journal.pmed.1004720 (PMC12422583; doi:10.1371/journal.pmed.1004720)
Supplement: S2 Table — (DOCX) [file pmed.1004720.s002.docx]

**S2 Table. Proportion in Individual and Composite State after Initial Elevated VL**

| **Time**  **(days)** | **Treatment failure, not returned** | **1 visit with no repeat VL** | **2 visits with no repeat VL** | **3+ visits with no repeat VL** | **Repeat VL Suppressed** | **Repeat VL Unsuppressed** | **Any VL repeated** | **Any Return Visit** | **Repeat VL among those who returned** | **VL Suppressed among those with Repeat VL** | **Current Treatment Interruption** | **Ever Treatment Interruption** | **Treatment Interruption among those without Repeat VL** | **Transfer** | **Death** |
| --- | --- | --- | --- | --- | --- | --- | --- | --- | --- | --- | --- | --- | --- | --- | --- |
| **Overall** | | | | | | | | | | | | | | | |
| **90** | 61  (60.2-61.9) | 19.3  (18.7-19.9) | 7  (6.5-7.4) | 2.9  (2.6-3.2) | 4.5  (4.1-4.8) | 2.9  (2.6-3.1) | 7.4  (6.9-7.8) | 37  (36.2-37.8) | 20  (18.9-21.2) | 60.8  (57.6-63.7) | 1.6  (1.4-1.8) | 1.7  (1.5-1.9) | 1.8 (1.5-2) | 0.4  (0.3-0.5) | 0.4  (0.3-0.5) |
| **180** | 13.7  (13.1-14.4) | 19.4  (18.7-20) | 8.2  (7.8-8.7) | 7.2  (6.8-7.7) | 25.2  (24.4-26) | 11.2  (10.6-11.7) | 36.4  (35.5-37.2) | 72.2  (71.3-73) | 49.9  (48.9-51) | 69.3  (68.1-70.6) | 13.7  (13.1-14.3) | 18.2  (17.5-18.8) | 22 (21.1-22.9) | 0.7  (0.6-0.9) | 0.6  (0.5-0.8) |
| **365** | 0  (0-0) | 1.6  (1.4-1.9) | 3.2  (2.8-3.5) | 6.6  (6.1-7) | 50.1  (49.2-51) | 20  (19.3-20.7) | 70.1  (69.3-70.9) | 87.6  (86.9-88.3) | 78.4  (77.6-79.3) | 71.5  (70.5-72.5) | 16.6  (15.9-17.2) | 32.5  (31.8-33.3) | 59.3 (57.5-61) | 1  (0.8-1.2) | 0.9  (0.7-1.1) |
| **730** | 0  (0-0) | 0  (0-0) | 0.1  (0-0.1) | 1.2  (0.9-1.6) | 58.1  (57.2-59.1) | 22.9  (22.1-23.7) | 81  (80.2-81.8) | 89.3  (88.6-90) | 89.1  (88.4-89.9) | 71.7  (70.8-72.7) | 15.3  (14.6-16.1) | 35.1  (34.3-36) | 92 (90.1-93.8) | 1.1  (0.9-1.3) | 1.2  (1-1.4) |
| **TLD** | | | | | | | | | | | | | | | |
| **90** | 69.6  (68.5-70.6) | 17.5  (16.6-18.3) | 4  (3.6-4.4) | 1.1  (0.9-1.4) | 4.6  (4.1-5.1) | 1.7  (1.4-2) | 6.3  (5.7-6.8) | 29.2  (28.1-30.3) | 21.4  (19.7-23.4) | 73.1  (68.9-77.1) | 1.0  (0.7-1.2) | 1  (0.8-1.2) | 1 (0.8-1.3) | 0.2  (0.1-0.3) | 0.4  (0.3-0.5) |
| **180** | 16.8  (15.9-17.8) | 23.9  (22.8-24.9) | 7.9  (7.2-8.5) | 5  (4.4-5.5) | 29.2  (28.1-30.3) | 5.4  (4.8-5.9) | 34.6  (33.4-35.7) | 70.3  (68.9-71.6) | 48.3  (46.7-49.8) | 84.5  (83-86) | 10.7  (9.9-11.4) | 14.6  (13.8-15.5) | 16.6 (15.5-17.8) | 0.6  (0.4-0.8) | 0.6  (0.4-0.7) |
| **365** | 0  (0-0) | 1.8  (1.4-2.2) | 4.1  (3.5-4.7) | 8  (7.3-8.8) | 59.2  (57.9-60.4) | 10.5  (9.7-11.3) | 69.7  (68.5-70.9) | 89  (87.9-90) | 76.7  (75.5-78) | 85  (83.9-86.1) | 14.4  (13.5-15.4) | 29.9  (28.7-31) | 50.7 (47.9-53.5) | 0.9  (0.7-1.1) | 1.0  (0.8-1.3) |
| **730** | 0  (0-0) | 0  (0-0) | 0  (0-0) | 2.4  (1.5-3.3) | 70.4  (69-71.9) | 12.5  (11.4-13.4) | 82.8  (81.4-84.2) | 90.4  (89.3-91.4) | 90.1  (88.8-91.4) | 85  (83.9-86.1) | 12.4  (11.1-13.7) | 32.9  (31.7-34.1) | 83.6 (77.9-89.4) | 0.9  (0.7-1.1) | 1.4  (1-1.8) |
| **TLE** | | | | | | | | | | | | | | | |
| **90** | 50.9  (49.6-52.1) | 21.5  (20.5-22.6) | 10.6  (9.8-11.3) | 5  (4.5-5.6) | 4.3  (3.8-4.9) | 4.3  (3.8-4.8) | 8.6  (8-9.3) | 46.4  (45.2-47.7) | 18.8  (17.4-20.4) | 50.3  (46.1-54.5) | 2.3  (1.9-2.7) | 2.5  (2.1-2.9) | 2.6 (2.2-3) | 0.6  (0.4-0.8) | 0.4  (0.3-0.6) |
| **180** | 10.1  (9.3-10.9) | 14.6  (13.6-15.4) | 8.7  (8-9.4) | 9.9  (9.2-10.7) | 21  (20-22) | 17.5  (16.6-18.4) | 38.5  (37.3-39.6) | 75.4  (74.3-76.6) | 51.2  (49.8-52.7) | 54.5  (52.7-56.6) | 16.6  (15.6-17.5) | 21.6  (20.6-22.6) | 27.7 (26.3-29.1) | 0.9  (0.7-1.2) | 0.7  (0.4-0.9) |
| **365** | 0  (0-0) | 1.4  (1.1-1.7) | 2.3  (1.9-2.7) | 5.6  (5-6.2) | 41.1  (40-42.2) | 29.5  (28.4-30.7) | 70.6  (69.5-71.8) | 87.7  (86.8-88.6) | 80  (79-81.1) | 58.2  (56.8-59.8) | 18.1  (17.1-19) | 34.6  (33.5-35.7) | 65.8 (63.7-68.2) | 1.2  (0.9-1.4) | 0.8  (0.6-1) |
| **730** | 0  (0-0) | 0  (0-0.1) | 0.1  (0-0.2) | 0.8  (0.5-1.1) | 47.3  (46.1-48.6) | 32.8  (31.5-34) | 80.1  (79-81.1) | 89.4  (88.5-90.2) | 89.3  (88.4-90.2) | 59.1  (57.7-60.5) | 16.6  (15.7-17.6) | 36.7  (35.5-37.8) | 94.7 (93.1-96.3) | 1.3  (1-1.5) | 1.1  (0.8-1.3) |
| **Difference between TLD vs TLE** | | | | | | | | | | | | | | | |
| **90** | 18.7  (17.1-20.2) | -4.1  (-5.4--2.7) | -6.6  (-7.4--5.7) | -3.9  (-4.5--3.3) | 0.2  (-0.5-0.9) | -2.6  (-3.2--2) | -2.4  (-3.3--1.5) | -17.2  (-18.8--15.6) | 2.6  (0.4-5) | 22.8  (16.2-28.8) | -1.4  (-1.8--0.9) | -1.5  (-1.9--1) | -1.6 (-2.1--1.1) | -0.4  (-0.6--0.2) | 0  (-0.2-0.2) |
| **180** | 6.7  (5.5-7.9) | 9.3  (7.9-10.7) | -0.8  (-1.7-0.2) | -5  (-5.9--4.1) | 8.2  (6.7-9.7) | -12.1  (-13.2--11) | -3.9  (-5.7--2.2) | -5.1  (-6.8--3.3) | -2.9  (-5.1--0.8) | 30  (27.4-32.3) | -5.9  (-7.1--4.7) | -7  (-8.2--5.6) | -11 (-12.8--9.3) | -0.4  (-0.7--0.1) | -0.1  (-0.3-0.2) |
| **365** | 0  (0-0) | 0.4  (-0.1-0.9) | 1.8  (1.1-2.5) | 2.4  (1.5-3.4) | 18.1  (16.4-19.7) | -19.1  (-20.5--17.7) | -0.9  (-2.7-0.7) | 1.3  (-0.2-2.6) | -3.4  (-4.9--1.7) | 26.8  (25-28.6) | -3.6  (-5--2.2) | -4.7  (-6.2--3.1) | -15.1 (-19--11.7) | -0.3  (-0.6-0.1) | 0.2  (-0.1-0.5) |
| **730** | 0  (0-0) | 0  (-0.1-0) | -0.1  (-0.2-0) | 1.6  (0.7-2.6) | 23.1  (21.1-24.9) | -20.3  (-21.8--18.7) | 2.8  (1-4.5) | 1.1  (-0.3-2.4) | 0.9  (-0.7-2.4) | 25.9  (24.1-27.6) | -4.3  (-5.8--2.7) | -3.8  (-5.4--2.1) | -11.1 (-17.2--4.9) | -0.4  (-0.7-0) | 0.4  (-0.1-0.8) |
