# Supplementary material for: Identifying care gaps along the HIV treatment failure cascade: A multistate analysis of viral load monitoring, re-suppression, and regimen switches in Zambia
Source: PLoS Med. 2025 Sep 3;22(9):e1004720. doi: 10.1371/journal.pmed.1004720 (PMC12422583; doi:10.1371/journal.pmed.1004720)
Supplement: S3 Table — (DOCX) [file pmed.1004720.s003.docx]

**S3 Table. Multistate results stratified by COVID-19 time-period & regimen**

| **After first Elevated VL** | | | | | | | |
| --- | --- | --- | --- | --- | --- | --- | --- |
| **Period of First Elevated VL** | **Pre-COVID**  (01 Aug 2019− 31 Mar 2020) | **COVID Lockdown**  (01 Apr 2020− 30 Sept 2020) | **Post-COVID Lockdown**  (01 Oct 2020− 30 Nov 2021) | **Period of First Elevated VL** | **Pre-COVID**  (01 Aug 2019− 31 Mar 2020) | **COVID Lockdown**  (01 Apr 2020− 30 Sept 2020) | **Post-COVID Lockdown**  (01 Oct 2020− 30 Nov 2021) |
| **TLD** | | | | **TLE** | | | |
| **Time (days)** | **Any visit** | | | **Time (days)** | **Any visit** | | |
| 90 | 41.1  (38.4-43.8) | 27.3  (24.7-29.8) | 26.5  (25.2-27.7) | 90 | 55.1  (53.4-56.7) | 25.6  (23.5-27.8) | 40.4  (37-43.7) |
| 180 | 74.9  (72.3-77.4) | 64.9  (62.1-68.1) | 71.1  (69.4-72.9) | 180 | 79.4  (78.1-80.7) | 66  (63.5-68.7) | 74.3  (71.1-77.5) |
| 365 | 90.5  (88.6-92.4) | 90.6  (88.3-92.7) | 87.9  (86.3-89.3) | 365 | 88.3  (87.3-89.4) | 87.7  (85.8-89.8) | 87.9  (85.2-90.5) |
| **Time (days)** | **Lost-to-follow-up** | | | **Time (days)** | **Lost-to-follow-up** | | |
| 90 | 2.3  (1.5-3) | 0.5  (0-0.8) | 0.7  (0.5-0.9) | 90 | 3  (2.5-3.5) | 0.9  (0.4-1.4) | 1.6  (0.7-2.5) |
| 180 | 14.4  (12.5-16.4) | 11.4  (9.6-13.2) | 9.1  (8.2-10) | 180 | 18.6  (17.4-19.8) | 12.6  (10.9-14.2) | 13.2  (10.8-15.3) |
| 365 | 14.7  (12.9-16.6) | 12.7  (10.7-14.7) | 14.7  (13.3-16.1) | 365 | 19.9  (18.7-21.1) | 13.7  (11.9-15.5) | 16.8  (14.1-19.7) |
| **Time (days)** | **VL checked** | | | **Time (days)** | **VL checked** | | |
| 90 | 6.9  (5.5-8.1) | 6.1  (4.7-7.5) | 6.2  (5.5-6.8) | 90 | 8.7  (7.8-9.6) | 7.2  (5.8-8.4) | 10.7  (8.6-12.7) |
| 180 | 34.9  (32.3-37.3) | 31  (28.4-33.5) | 35.6  (34.2-37.1) | 180 | 36.8  (35.2-38.2) | 37.7  (35.2-40.3) | 48.2  (45.2-51.6) |
| 365 | 68.7  (66.3-71.2) | 74.7  (72.1-77.2) | 67.4  (65.6-69.2) | 365 | 67.9  (66.5-69.2) | 76.5  (74.2-78.8) | 72.9  (69.7-76.2) |
| **Time (days)** | **Suppressed** | | | **Time (days)** | **Suppressed** | | |
| 90 | 4.4  (3.3-5.4) | 4.6  (3.3-5.8) | 4.7  (4-5.2) | 90 | 4.1  (3.4-4.6) | 3.2  (2.3-4) | 7.5  (5.6-9.2) |
| 180 | 29.2  (26.6-31.4) | 23.8  (21.3-26.2) | 30.8  (29.4-32.2) | 180 | 17.7  (16.5-18.9) | 21.7  (19.6-23.9) | 35.4  (32.5-38.6) |
| 365 | 57.1  (54.3-59.7) | 60.4  (57.3-63.4) | 59  (57.3-60.8) | 365 | 35.3  (33.8-36.7) | 49.5  (46.9-52) | 54.7  (51-58.1) |
| **Time (days)** | **Percent Suppressed** | | | **Time (days)** | **Percent Suppressed** | | |
| 90 | 64.2  (54.5-74.2) | 75  (64-85.7) | 75.5  (70.6-80.7) | 90 | 46.7  (41.6-51.7) | 44.8  (35-54.8) | 69.8  (60-78.4) |
| 180 | 83.7  (80.2-87.2) | 76.7  (72.4-81.3) | 86.5  (84.8-88) | 180 | 48.1  (45.5-50.8) | 57.7  (53.5-62) | 73.4  (69.2-77.6) |
| 365 | 83.1  (80.5-85.7) | 80.9  (77.9-83.5) | 87.6  (86.2-89) | 365 | 52  (50.1-53.8) | 64.7  (62-67.4) | 75  (71.6-78.4) |
| **After second Elevated VL** | | | | | | | |
| **Period of First Elevated VL** | **Pre-COVID**  (01 Aug 2019− 31 Mar 2020) | **COVID Lockdown**  (01 Apr 2020− 30 Sept 2020) | **Post-COVID Lockdown**  (01 Oct 2020− 30 Nov 2021) | **Period of First Elevated VL** | **Pre-COVID**  (01 Aug 2019− 31 Mar 2020) | **COVID Lockdown**  (01 Apr 2020− 30 Sept 2020) | **Post-COVID Lockdown**  (01 Oct 2020− 30 Nov 2021) |
| **TLD** | | | | **TLE** | | | |
| **Time (days)** | **Any visit** | | | **Time (days)** | **Any visit** | | |
| 90 | 80.6  (70-92.9) | 42.6  (32.4-51.9) | 60  (55.6-64.5) | 90 | 82.4  (79.4-85.4) | 50.6  (46.7-54.5) | 63.5  (60.1-67.1) |
| 180 | 92.5  (81.2-100) | 85  (72.4-94.3) | 88.4  (84.1-92.3) | 180 | 91.7  (89.4-93.6) | 88.9  (85.1-91.8) | 89.7  (87.2-92) |
| 365 | 97.7  (92.6-100) | 97.4  (93.4-100) | 97.2  (94.9-98.9) | 365 | 95.6  (93.9-96.9) | 95.4  (92.3-97.6) | 95.9  (93.9-97.6) |
| **Time (days)** | **Lost-to-follow-up** | | | **Time (days)** | **Lost-to-follow-up** | | |
| 90 | 0  (0-0) | 1.9  (0-3.6) | 3  (1.3-4.5) | 90 | 2.9  (1.4-4.2) | 0.8  (0-1.3) | 1.9  (0.8-2.9) |
| 180 | 6.5  (0-12.5) | 18.5  (10-26.7) | 16.1  (12.3-19.5) | 180 | 13  (10.1-15.6) | 9.5  (7.3-11.7) | 8.8  (6.6-11) |
| 365 | 6.5  (0-12.5) | 14.8  (6.7-22.2) | 20.1  (15.1-24.9) | 365 | 13.3  (10.5-16.1) | 11.1  (8.7-13.2) | 9.2  (6.8-11.9) |
| **Time (days)** | **Switched regimens** | | | **Time (days)** | **Switched regimens** | | |
| 90 | 25.8  (13.3-37.5) | 13  (5.9-19.4) | 16.8  (13.2-20) | 90 | 31  (27.1-34.9) | 26.7  (23.6-30.1) | 36.4  (32.9-39.9) |
| 180 | 35.5  (20-50) | 22.2  (13.3-31.2) | 23  (19.3-27.1) | 180 | 49  (44.7-52.9) | 54.8  (51.2-58.7) | 51  (47.3-54.8) |
| 365 | 49.5  (35-63.9) | 27.8  (18.4-37.5) | 23  (19.3-27.1) | 365 | 65.8  (61.9-69.5) | 73.8  (70.7-77) | 59.1  (55-63) |
| **Time (days)** | **VL checked** | | | **Time (days)** | **VL checked** | | |
| 90 | 12.9  (4.5-21.4) | 11.1  (5-17.1) | 7.7  (5.1-10) | 90 | 7.7  (5.4-9.8) | 5.9  (4.1-7.7) | 6.8  (5-8.5) |
| 180 | 38.7  (25-52.9) | 29.6  (21.2-38.9) | 33.2  (28.5-38) | 180 | 20.6  (17.2-23.8) | 22.4  (19.4-25.5) | 24.7  (21.5-27.9) |
| 365 | 50.8  (36.1-65.1) | 50  (39.3-60.6) | 52.2  (45-58.6) | 365 | 31.2  (27.2-34.9) | 31.4  (27.9-34.8) | 33.3  (29.7-37.4) |
| **Time (days)** | **Suppressed** | | | **Time (days)** | **Suppressed** | | |
| 90 | 6.5  (0-12.5) | 9.3  (3-15.6) | 4.6  (2.7-6.3) | 90 | 1.5  (0.5-2.4) | 1.3  (0.4-2.1) | 3.6  (2.2-5) |
| 180 | 25.8  (13.3-38.1) | 22.2  (12.9-30.8) | 22.9  (18.6-26.6) | 180 | 6.5  (4.3-8.3) | 5.9  (4.1-7.6) | 15.7  (12.8-18.4) |
| 365 | 32.3  (18.7-46.2) | 40.7  (30-50) | 40.5  (34.2-47.2) | 365 | 10.6  (8-13) | 11.1  (8.7-13.2) | 24.1  (20.2-27.5) |
| **Time (days)** | **Percent Suppressed** | | | **Time (days)** | **Percent Suppressed** | | |
| 90 | 50  (0-100) | 83.3  (50-100) | 59.9  (43.4-73.8) | 90 | 19.2  (6.2-30.8) | 17.4  (6.2-28.6) | 53.9  (38.8-68.6) |
| 180 | 66.7  (42.9-87.5) | 75  (57.1-90.9) | 66.6  (58.7-74) | 180 | 30  (21.9-38.1) | 25.3  (17.6-32.5) | 61.6  (54.2-68.9) |
| 365 | 63.5  (45-87.5) | 77.8  (66.6-91.7) | 70.9  (63-78.6) | 365 | 31.2  (24.7-37.7) | 31.2  (24.5-37.3) | 62.6  (55.9-69.6) |
