# Supplementary material for: Identifying care gaps along the HIV treatment failure cascade: A multistate analysis of viral load monitoring, re-suppression, and regimen switches in Zambia
Source: PLoS Med. 2025 Sep 3;22(9):e1004720. doi: 10.1371/journal.pmed.1004720 (PMC12422583; doi:10.1371/journal.pmed.1004720)
Supplement: S4 Table — (DOCX) [file pmed.1004720.s004.docx]

**S4 Table. Characteristics of those with a second elevated viral load**

|  |  | Total  (N=1,540) | TLD  (n=390) | TLE  (n=1,150) |
| --- | --- | --- | --- | --- |
| Gender | Female | 889 (57.7%) | 164 (42.1%) | 725 (63.0%) |
|  | Male | 545 (35.4%) | 194 (49.7%) | 351 (30.5%) |
|  | Missing | 106 (6.9%) | 32 (8.2%) | 74 (6.4%) |
| Age Category | 18-24 | 194 (12.6%) | 47 (12.1%) | 147 (12.8%) |
|  | 25-34 | 489 (31.8%) | 121 (31.0%) | 368 (32.0%) |
|  | 35-44 | 526 (34.2%) | 113 (29.0%) | 413 (35.9%) |
|  | 45-54 | 177 (11.5%) | 56 (14.4%) | 121 (10.5%) |
|  | 55+ | 48 (3.1%) | 21 (5.4%) | 27 (2.3%) |
|  | Missing | 106 (6.9%) | 32 (8.2%) | 74 (6.4%) |
| Age, median (IQR) |  | 35.5 (28.6-42.1) | 35.6 (28.6-43.1) | 35.4 (28.6-41.6) |
| Marital Status | Single | 255 (16.6%) | 64 (16.4%) | 191 (16.6%) |
|  | Married | 682 (44.3%) | 166 (42.6%) | 516 (44.9%) |
|  | Divorced | 155 (10.1%) | 39 (10.0%) | 116 (10.1%) |
|  | Widowed | 83 (5.4%) | 23 (5.9%) | 60 (5.2%) |
|  | Missing | 365 (23.7%) | 98 (25.1%) | 267 (23.2%) |
| Education | None | 82 (5.3%) | 13 (3.3%) | 69 (6.0%) |
|  | Primary | 345 (22.4%) | 77 (19.7%) | 268 (23.3%) |
|  | Secondary | 703 (45.6%) | 189 (48.5%) | 514 (44.7%) |
|  | University | 92 (6.0%) | 29 (7.4%) | 63 (5.5%) |
|  | Missing | 318 (20.6%) | 82 (21.0%) | 236 (20.5%) |
| WHO Stage | 1 | 682 (44.3%) | 178 (45.6%) | 504 (43.8%) |
|  | 2 | 188 (12.2%) | 47 (12.1%) | 141 (12.3%) |
|  | 3 | 296 (19.2%) | 68 (17.4%) | 228 (19.8%) |
|  | 4 | 14 (0.9%) | 4 (1.0%) | 10 (0.9%) |
|  | Missing | 360 (23.4%) | 93 (23.8%) | 267 (23.2%) |
| ART regimen at First Elevated VL | TLD | 390 (25.3%) | 390 (100.0%) | 0 (0.0%) |
|  | TLE | 1,150 (74.7%) | 0 (0.0%) | 1,150 (100.0%) |
| Year of ART initiation | 2004-2010 | 219 (14.2%) | 42 (10.8%) | 177 (15.4%) |
|  | 2011-2016 | 575 (37.3%) | 100 (25.6%) | 475 (41.3%) |
|  | 2017-2018 | 507 (32.9%) | 109 (27.9%) | 398 (34.6%) |
|  | 2019-2021 | 239 (15.5%) | 139 (35.6%) | 100 (8.7%) |
| Time on ART at First Elevated VL | <1 year | 185 (12.0%) | 85 (21.8%) | 100 (8.7%) |
|  | 1-2 years | 323 (21.0%) | 91 (23.3%) | 232 (20.2%) |
|  | 2-5 years | 444 (28.8%) | 104 (26.7%) | 340 (29.6%) |
|  | 5-10 years | 414 (26.9%) | 74 (19.0%) | 340 (29.6%) |
|  | 10+ years | 174 (11.3%) | 36 (9.2%) | 138 (12.0%) |
| Time from First Elevated VL to Next Scheduled Appointment | 30 days | 266 (17.3%) | 37 (9.5%) | 229 (19.9%) |
|  | 60 days | 35 (2.3%) | 8 (2.1%) | 27 (2.3%) |
|  | 90 days | 789 (51.2%) | 181 (46.4%) | 608 (52.9%) |
|  | 120 days | 163 (10.6%) | 51 (13.1%) | 112 (9.7%) |
|  | 150 days | 46 (3.0%) | 18 (4.6%) | 28 (2.4%) |
|  | 180 days | 241 (15.6%) | 95 (24.4%) | 146 (12.7%) |
| Time between First and Second VL | 30-180 days | 830 (53.9%) | 209 (53.6%) | 621 (54.0%) |
|  | 180-365 days | 581 (37.7%) | 153 (39.2%) | 428 (37.2%) |
|  | 1-2 years | 129 (8.4%) | 28 (7.2%) | 101 (8.8%) |
|  | >2 years | 0 (0.0%) | 0 (0.0%) | 0 (0.0%) |
|  | Missing | 0 (0.0%) | 0 (0.0%) | 0 (0.0%) |
| Facility size | Small Health Centre | 53 (3.4%) | 17 (4.4%) | 36 (3.1%) |
|  | Medium Health Centre | 348 (22.6%) | 93 (23.8%) | 255 (22.2%) |
|  | Large Health Centre | 427 (27.7%) | 108 (27.7%) | 319 (27.7%) |
|  | Hospital | 712 (46.2%) | 172 (44.1%) | 540 (47.0%) |

Abbreviations: VL, Viral Load; TLD, tenofovir disoproxil fumarate/lamivudine/dolutegravir TDF/FTC/DTG; TLE, tenofovir disoproxil fumarate/lamivudine/efavirenz TDF/FTC/EFV. Small Health Center: <2,500 clients; Medium Health Center: 2,500-7,500 clients; Large Health Center: 7,500-20,000 client
