# Supplementary material for: Identifying care gaps along the HIV treatment failure cascade: A multistate analysis of viral load monitoring, re-suppression, and regimen switches in Zambia
Source: PLoS Med. 2025 Sep 3;22(9):e1004720. doi: 10.1371/journal.pmed.1004720 (PMC12422583; doi:10.1371/journal.pmed.1004720)
Supplement: S5 Table — (DOCX) [file pmed.1004720.s005.docx]

**S5 Table. Proportion in Individual and Composite State after Two Elevated VLs and Due for Switch**

| **Time**  **(days)** | **Treatment failure, due for switch** | **1 visit with no switch** | **2 visits with no switch** | **3+ visits with no switch** | **Regimen switch** | **Repeat VL Suppressed** | **Repeat VL Unsuppressed** | **Any VL repeated** | **Any Return Visit** | **Suppressed among those with Repeat VL** | **Regimen Switch or VL Suppressed** | **Current Treatment Interruption** | **Ever Treatment Interruption** | **Treatment Interruption among those not switched or suppressed** | **Transfer** | **Death** |
| --- | --- | --- | --- | --- | --- | --- | --- | --- | --- | --- | --- | --- | --- | --- | --- | --- |
| **Overall** | | | | | | | | | | | | | | | | |
| **90** | 34.3  (32.2-36.1) | 20.2  (18.7-21.8) | 6.5  (5.6-7.5) | 2.4  (1.8-3) | 27.9  (26.2-29.7) | 3  (2.3-3.6) | 3.2  (2.6-3.8) | 7.2  (6.1-8.2) | 63.3  (61.5-65.2) | 40.5  (33.9-48.2) | 30.9  (29.2-32.6) | 2  (1.5-2.6) | 2.2  (1.6-2.8) | 2.9 (2.2-3.7) | 0  (0-0) | 0.5  (0.2-0.7) |
| **180** | 2.9  (2.2-3.5) | 8.8  (7.7-10) | 6  (4.9-6.9) | 3.8  (3-4.6) | 45.3  (43.4-47.4) | 12.7  (11.4-14) | 8.4  (7.3-9.4) | 25.1  (23.4-26.9) | 89.2  (87.9-90.6) | 48.9  (44.9-52.9) | 58  (56.2-59.9) | 11.6  (10.3-12.8) | 17  (15.4-18.4) | 28 (25.3-30.7) | 0  (0-0) | 0.5  (0.2-0.7) |
| **365** | 0  (0-0) | 0.4  (0.1-0.6) | 0.7  (0.3-1) | 2.4  (1.7-3.1) | 58.6  (56.6-60.7) | 20.4  (18.7-22) | 4.2  (3.3-5.1) | 36.6  (34.5-38.4) | 95.8  (94.9-96.7) | 51  (47.8-54.5) | 79  (77.4-80.7) | 13.2  (11.7-14.5) | 23.4  (21.6-24.8) | 47.5 (43.6-51.3) | 0  (0-0) | 0.8  (0.4-1.1) |
| **540** | 0  (0-0) | 0  (0-0.2) | 0  (0-0.5) | 0.9  (0.3-1.4) | 62.3  (60.3-64.5) | 21.6  (19.8-23.4) | 1.0  (0.5-1.6) | 38.7  (36.6-40.6) | 95.9  (95.1-96.8) | 50.1  (46.5-53.6) | 83.9  (82.2-85.7) | 12.6  (11.1-13.9) | 23.6  (22-25.2) | 62.2 (57.3-67.1) | 0  (0-0) | 0.8  (0.4-1.1) |
| **TLD** | | | | | | | | | | | | | | | | |
| **90** | 38  (34.2-41.8) | 24.8  (21.5-28.3) | 6.2  (4.3-8) | 3.5  (2.1-4.9) | 17  (14.3-20) | 5.4  (3.5-7.2) | 2.1  (0.9-3.2) | 8.6  (6.3-10.6) | 59.1  (55.1-63.1) | 63  (50.4-77.8) | 22.4  (19.3-25.6) | 2.6  (1.3-3.8) | 2.6  (1.3-3.8) | 3.3 (1.7-4.9) | 0  (0-0) | 0.3  (0-0.5) |
| **180** | 3.1  (1.5-4.5) | 13.2  (10.2-15.7) | 7.2  (4.9-9.5) | 5.3  (3.4-7.2) | 24  (20.5-27.5) | 23.2  (19.9-26.8) | 8.1  (5.8-10.4) | 33.2  (29.4-37.3) | 88.4  (85-91.4) | 68  (61.6-75.2) | 47.2  (43.3-51.3) | 15.6  (12.1-18.6) | 22.7  (19.1-25.8) | 29.6 (23.7-35.1) | 0  (0-0) | 0.3  (0-0.5) |
| **365** | 0  (0-0) | 0  (0-0) | 2.2  (0.6-3.6) | 6.4  (3.1-9.2) | 27.9  (24.1-31.5) | 39.3  (34.6-44.4) | 6.6  (3.4-9.6) | 52.4  (47.2-57.4) | 97.1  (95.5-98.4) | 70  (64.1-76.6) | 67.2  (62.5-71.9) | 17.1  (13.6-20.4) | 29.5  (25.7-33) | 41 (33.6-48.2) | 0  (0-0) | 0.3  (0-0.5) |
| **540** | 0  (0-0) | 1.1  (0-2.3) | 1.5  (0-2.8) | 5.6  (1.8-9.7) | 29.9  (25.5-34.4) | 45.9  (38.2-51.8) | 0  (0-5.2) | 55.9  (50.3-60.7) | 97.4  (96.1-98.7) | 68.4  (62.1-75.6) | 75.9  (68.8-80.7) | 17.3  (13.5-20.9) | 30.6  (26.5-34.5) | 53.3 (43-62.8) | 0  (0-0) | 0.3  (0-0.5) |
| **TLE** | | | | | | | | | | | | | | | | |
| **90** | 33  (30.9-35.1) | 18.7  (17-20.4) | 6.7  (5.6-7.7) | 2  (1.4-2.6) | 31.5  (29.4-33.7) | 2.2  (1.5-2.8) | 3.6  (2.8-4.3) | 6.7  (5.6-7.8) | 70.1  (62.7-67) | 31.2  (22.8-39.7) | 33.7  (31.5-35.8) | 1.8  (1.2-2.4) | 2.1  (1.5-2.7) | 2.8 (1.9-3.7) | 0  (0-0) | 0.5  (0.1-0.8) |
| **180** | 2.8  (2-3.5) | 7.6  (6.4-8.7) | 5.6  (4.4-6.6) | 3.4  (2.6-4.3) | 51.7  (49.5-54.2) | 9.6  (8.3-10.9) | 8.4  (7.2-9.6) | 22.6  (20.9-24.6) | 103.3  (88.2-91.1) | 40.7  (36.1-45.4) | 61.3  (59.2-63.6) | 10.4  (8.9-11.7) | 15.1  (13.5-16.7) | 27.2 (24.3-30.3) | 0  (0-0) | 0.6  (0.3-1) |
| **365** | 0  (0-0) | 0.5  (0.2-0.7) | 0.3  (0-0.6) | 1.5  (1-2.1) | 66.6  (64.5-68.9) | 15.2  (13.6-16.9) | 3.6  (2.7-4.5) | 32.1  (29.9-34.1) | 117.6  (94.6-96.6) | 42.9  (39.1-47) | 81.9  (80.1-83.7) | 12  (10.5-13.5) | 21.2  (19.4-22.8) | 50.2 (45.6-54.9) | 0  (0-0) | 1  (0.6-1.4) |
| **540** | 0  (0-0) | 0  (0-0) | 0  (0-0.2) | 0.2  (0-0.5) | 70.2  (68.2-72.5) | 15.7  (14-17.4) | 1  (0.4-1.5) | 33.6  (31.4-35.8) | 114.3  (94.7-96.7) | 41.9  (38-46) | 85.9  (84.3-87.8) | 11.2  (9.7-12.6) | 21.3  (19.6-22.9) | 65.3 (59.8-71) | 0  (0-0) | 1  (0.6-1.4) |
| **Difference between TLD vs TLE** | | | | | | | | | | | | | | | | |
| **90** | 5  (0.9-9.3) | 6.2  (2.4-10.2) | -0.5  (-2.7-1.8) | 1.5  (0-3.1) | -14.5  (-18.3--11) | 3.2  (1.2-5.1) | -1.4  (-2.8-0) | 1.8  (-0.6-4.3) | -11  (-10.1--1.2) | 31.7  (17.1-48.1) | -11.3  (-15.5--7.4) | 0.7  (-0.6-2.2) | 0.5  (-0.9-1.7) | 0.5 (-1.4-2.3) | 0  (0-0) | -0.2  (-0.7-0.2) |
| **180** | 0.3  (-1.5-2) | 5.6  (2.3-8.3) | 1.7  (-0.9-4.2) | 1.9  (-0.3-4) | -27.7  (-32.1--23.5) | 13.6  (10-17.5) | -0.3  (-3-2.5) | 10.6  (6.4-14.8) | -14.9  (-4.9-2.1) | 27.3  (19.6-35.1) | -14.1  (-18.9--9.4) | 5.2  (1.6-8.7) | 7.5  (3.5-11.1) | 2.5 (-4.1-8.8) | 0  (0-0) | -0.3  (-0.8-0.2) |
| **365** | 0  (0-0) | -0.5  (-0.7--0.2) | 1.8  (0.2-3.3) | 4.9  (1.6-7.7) | -38.7  (-43.6--34.5) | 24.1  (19.1-29.4) | 2.9  (-0.4-6) | 20.3  (14.9-25.6) | -20.6  (-0.3-3.1) | 27.1  (19.6-34.3) | -14.6  (-20--9.6) | 5  (1.1-8.7) | 8.3  (3.9-12.2) | -9.2 (-18.1--0.9) | 0  (0-0) | -0.7  (-1.3--0.1) |
| **540** | 0  (0-0) | 1.1  (0-2.3) | 1.5  (0-2.8) | 5.4  (1.6-9.5) | -40.3  (-45.6--35.5) | 30.3  (22.1-36.3) | -1  (-1.4-4.2) | 22.3  (16.5-27.4) | -16.9  (0.1-3.3) | 26.4  (19-34.4) | -10.1  (-17.2--5.5) | 6.1  (2.1-10.1) | 9.3  (4.6-13.5) | -12.1 (-23.2--1.7) | 0  (0-0) | -0.7  (-1.3--0.1) |
