# Supplementary material for: Identifying care gaps along the HIV treatment failure cascade: A multistate analysis of viral load monitoring, re-suppression, and regimen switches in Zambia
Source: PLoS Med. 2025 Sep 3;22(9):e1004720. doi: 10.1371/journal.pmed.1004720 (PMC12422583; doi:10.1371/journal.pmed.1004720)
Supplement: S6 Table — (DOCX) [file pmed.1004720.s006.docx]

**S6 Table. Cox proportional hazards model for predictors of return, VL check and suppress after first VL elevated**

| **Factor** | **Level** | **Time to return**  **(N=7916)** | | **Time to VL check**  **(N=7916)** | | **Time to resuppression after initial treatment failure**  **(N=7916)** | | **Time to switch after due for switch**  **(N=1,540)** | | **Time to resuppression after due for switch**  **(N=1,540)** | |
| --- | --- | --- | --- | --- | --- | --- | --- | --- | --- | --- | --- |
|  |  | Adjusted Hazards ratio (aHR), 95% CI | P-value | Adjusted Hazards ratio  (aHR), 95% CI | P-value | Adjusted Hazards ratio  (aHR), 95% CI | P-value | Adjusted Hazards ratio  (aHR), 95% CI | P-value | Adjusted Hazards ratio  (aHR), 95% CI | P-value |
| **Drug regimen** | TLE | 1.0  (base) | 0.335 | 1.0  (base) | 0.452 | 1.0  (base) | 0.000 | 1.0  (base) | 0.000 | 1.0  (base) | 0.007 |
|  | TLD | 1.03  (0.97-1.09) |  | 1.03  (0.96-1.10) |  | 1.42  (1.31-1.54) |  | 0.37  (0.29-0.47) |  | 1.49  (1.12-2.01) |  |
| **Gender** | Female | 1.0  (base) | 0.000 | 1.0  (base) | 0.000 | 1.0  (base) | 0.000 | 1.0  (base) | 0.714 | 1.0  (base) | 0.212 |
|  | Male | 0.87  (0.81-.92) |  | 0.78  (0.73-0.83) |  | 0.75  (0.69-0.80) |  | 0.97  (0.82-1.15) |  | 0.83  (0.62-1.11) |  |
| **Age category** | 18-24 | 1.0  (base) | 0.0000 | 1.0  (base) | 0.0059 | 1.0  (base) | 0.0000 | 1.0  (base) | 0.3313 | 1.0  (base) | 0.1817 |
|  | 25-34 | 1.05  (0.94-1.18) |  | 1.05  (0.93-1.19) |  | 1.03  (0.89-1.19) |  | 1.15  (0.88-1.50) |  | 0.78  (0.49-1.21) |  |
|  | 35-44 | 1.21  (1.06-1.37) |  | 1.13  (0.99-1.28) |  | 1.15  (0.99-1.34) |  | 1.29  (0.99-1.71) |  | 0.95  (0.59-1.53) |  |
|  | 45-54 | 1.24  (1.09-1.43) |  | 1.24  (1.07-1.42) |  | 1.36  (1.15-1.60) |  | 1.24  (0.88-1.76) |  | 1.29  (0.76-2.22) |  |
|  | 55+ | 1.43  (1.21-1.71) |  | 1.29  (1.07-1.54) |  | 1.46  (1.19-1.79) |  | 1.45  (0.89-2.37) |  | 0.78  (0.36-1.72) |  |
| **Appointment interval** | 1 month | 1.0  (base) | 0.0000 | 1.0  (base) | 0.0001 | 1.0  (base) | 0.0004 | 1.0  (base) | 0.0002 | 1.0  (base) | 0.7609 |
|  | 60 days | 0.80  (0.66-0.98) |  | 1.24  (0.99-1.53) |  | 1.34  (1.03-1.74) |  | 0.82  (0.49-1.35) |  | 1.73  (0.78-3.84) |  |
|  | 90 days | 0.45  (0.42-0.49) |  | 0.99  (0.91-1.09) |  | 0.99  (0.89-1.11) |  | 0.71  (0.59-0.85) |  | 1.17  (0.83-1.64) |  |
|  | 120 days | 0.42  (0.38-0.46) |  | 0.99  (0.89-1.11) |  | 1.11  (0.98-1.27) |  | 0.86  (0.70-1.06) |  | 1.15  (0.77-1.71) |  |
|  | 150 days | 0.34  (0.29-.39) |  | 0.91  (0.77-1.07) |  | 0.92  (0.76-1.11) |  | 0.79  (0.43-1.43) |  | 1.52  (0.66-3.52) |  |
|  | 180 days | 0.25  (0.23-0.28) |  | 0.85  (0.76-0.94) |  | 0.89  (0.79-1.01) |  | 0.36  (0.21-0.61) |  | 1.12  (0.57-2.21) |  |
| **Time on ART** | < 1 year | 1.0  (base) | 0.0000 | 1.0  (base) | 0.0000 | 1.0  (base) | 0.0000 | 1.0  (base) | 0.0012 | 1.0  (base) | 0.1683 |
|  | 1-2 years | 1.03  (0.95-1.13) |  | 1.11  (0.99-1.23) |  | 1.12  (0.99-1.27) |  | 0.88  (0.68-1.16) |  | 0.96  (0.61-1.52) |  |
|  | 2-5 years | 1.04  (0.95-1.13) |  | 1.22  (1.11-1.34) |  | 1.36  (1.21-1.53) |  | 0.97  (0.76-1.26) |  | 1.38  (0.90-2.11) |  |
|  | 5-10 years | 1.19  (1.08-1.30) |  | 1.42  (1.28-1.57) |  | 1.56  (1.38-1.76) |  | 0.98  (0.75-1.29) |  | 1.37  (0.86-2.17) |  |
|  | 10+ years | 1.19  (1.06-1.34) |  | 1.46  (1.28-1.65) |  | 1.54  (1.32-1.79) |  | 1.52  (1.11-2.10) |  | 1.63  (0.91-2.92) |  |
| **HIV enrolment stage** | WHO Stage 1 | 1.0  (base) | 0.6022 | 1.0  (base) | 0.1327 | 1.0  (base) | 0.0437 | 1.0  (base) | 0.0551 | 1.0  (base) | 0.5420 |
|  | WHO Stage 2 | 0.94  (0.87-1.03) |  | 0.96  (0.88-1.05) |  | 0.95  (0.86-1.06) |  | 0.78  (0.61-0.99) |  | 0.91  (0.59-1.41) |  |
|  | WHO Stage 3 | 0.97  (0.89-1.06) |  | 0.94  (0.87-1.02) |  | 0.88  (0.79-0.98) |  | 0.78  (0.63-0.96) |  | 1.25  (0.84-1.85) |  |
|  | WHO Stage 4 | 1.02  (0.79-1.30) |  | 0.75  (0.57-0.99) |  | 0.74  (0.53-1.03) |  | 0.62  (0.29-1.29) |  | 1.24  (0.38-4.03) |  |
| **Marital status** | Single | 1.0  (base) | 0.0226 | 1.0  (base) | 0.0042 | 1.0  (base) | 0.0106 | 1.0  (base) | 0.8738 | 1.0  (base) | 0.0660 |
|  | Married | 0.93  (0.83-1.04) |  | 0.97  (0.87-1.07) |  | 1.04  (0.93-1.17) |  | 1.11  (0.86-1.44) |  | 1.65  (1.07-2.52) |  |
|  | Divorced | 0.83  (0.72-0.95) |  | 0.82  (0.72-0.93) |  | 0.87  (0.75-0.99) |  | 1.07  (0.79-1.45) |  | 1.51  (0.93-2.46) |  |
|  | Widowed | 0.91  (0.78-1.06) |  | 0.97  (0.83-1.13) |  | 1.03  (0.87-1.23) |  | 1.08  (0.74-1.58) |  | 1.04  (0.49-2.19) |  |
| **Education category** | No education | 1.0  (base) | 0.4172 | 1.0  (base) | 0.3594 | 1.0  (base) | 0.8296 | 1.0  (base) | 0.9640 | 1.0  (base) | 0.3654 |
|  | Primary | 1.07  (0.95-1.21) |  | 1.03  (0.91-1.18) |  | 1.06  (0.89-1.24) |  | 1.00  (0.74 - 1.35) |  | 0.77  (0.46-1.29) |  |
|  | Secondary | 1.02  (0.91-1.16) |  | 1.01  (0.89-1.15) |  | 1.02  (0.88-1.19) |  | 0.98  (0.72- 1.31) |  | 0.65  (0.39-1.08) |  |
|  | University | 1.09  (0.92-1.29) |  | 1.13  (0.94-1.34) |  | 1.03  (0.84-1.28) |  | 1.05  (0.69-1.62) |  | 0.63  (0.29-1.35) |  |
| **Time period** | Prior to COVID Lockdown  (Prior to April 1, 2020) | 1.0  (base) | 0.1151 | 1.0  (base) | 0.0124 | 1.0  (base) | 0.0002 | 1.0  (base) | 0.0994 | 1.0  (base) | 0.0000 |
|  | COVID Lockdown  (April 1, 2020 to Oct 1, 2020) | 0.93  (0.86-0.99) |  | 1.12  (1.04-1.20) |  | 1.19  (1.09-1.30) |  | 1.22  (1.01-1.47) |  | 1.24  (0.83-1.86) |  |
|  | Post COVID Lockdown  (after Oct 1, 2020) | 0.96  (0.90 -1.03) |  | 1.02  (0.95-1.11) |  | 1.18  (1.07-1.29) |  | 1.07  (0.89-1.290 |  | 2.43  (1.67-3.53) |  |
| **Facility size** | Small Health Centre | 1.0  (base) | 0.0640 | 1.0  (base) | 0.0000 | 1.0  (base) | 0.0000 | 1.0  (base) | 0.0037 | 1.0  (base) | 0.0166 |
|  | Medium Health Centre | 1.24  (0.91-1.68) |  | 1.63  (1.17-2.28) |  | 1.72  (1.18-2.52) |  | 0.89  (0.35-2.30) |  | 0.57  (0.18-1.79) |  |
|  | Large Health Centre | 1.38  (1.01-1.88) |  | 1.64  (1.17-2.31) |  | 1.61  (1.09-2.39) |  | 0.90  (0.35-2.31) |  | 0.40  (0.12-1.36) |  |
|  | Hospital | 1.15  (0.85-1.56) |  | 1.09  (0.78-1.54) |  | 1.15  (0.78-1.68) |  | 0.36  (0.13-0.98) |  | 0.19  (0.06-0.69) |  |
