# Supplementary material for: Identifying care gaps along the HIV treatment failure cascade: A multistate analysis of viral load monitoring, re-suppression, and regimen switches in Zambia
Source: PLoS Med. 2025 Sep 3;22(9):e1004720. doi: 10.1371/journal.pmed.1004720 (PMC12422583; doi:10.1371/journal.pmed.1004720)
Supplement: S1 Fig — (DOCX) [file pmed.1004720.s007.docx]

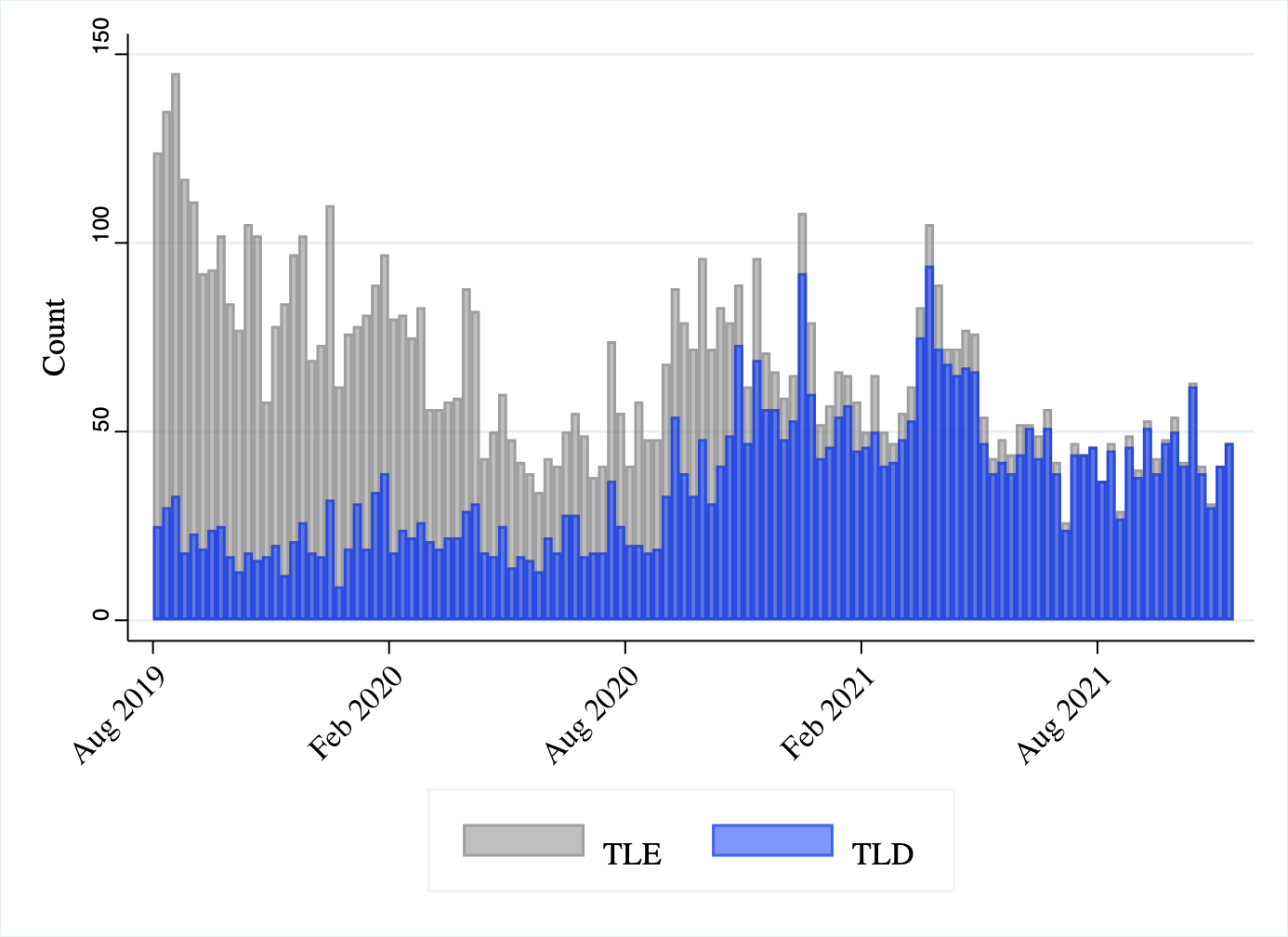
**S1 Fig. Distribution of Individuals with Elevated Viral on TLE versus TLD over time.** This figure illustrates the distribution of elevated VL counts (Y axis) across the study period from August 2019 to August 2021(X axis). The data show a decrease in the number of elevated VLs during the COVID-19 pandemic. However, some of this decline may also be attributed to the national transition to TLD in Zambia.

Abbreviations: TLD, tenofovir disoproxil fumarate/lamivudine or emtricitabine/dolutegravir [TDF/XTC/DTG]; TLE, tenofovir disoproxil fumarate/lamivudine or emtricitabine/efavirenz [TDF/XTC/EFV]; VL, Viral Load
