# Supplementary material for: Identifying care gaps along the HIV treatment failure cascade: A multistate analysis of viral load monitoring, re-suppression, and regimen switches in Zambia
Source: PLoS Med. 2025 Sep 3;22(9):e1004720. doi: 10.1371/journal.pmed.1004720 (PMC12422583; doi:10.1371/journal.pmed.1004720)
Supplement: S2 Fig — (DOCX) [file pmed.1004720.s008.docx]

**S2 Fig. Transition Hazards between States over Time after Initial Elevated VL.** This figure presents five panels of instantaneous hazard rates over time for individuals on TLD (blue line) and TLE (red line) following an initial elevated viral load. The x-axis in each panel represents time in days (0–540) since the specified event, and the y-axis denotes the instantaneous hazard rate. a) Time to Return: Hazard of returning to care after an initial elevated VL; b) Time to Treatment Interruption: Hazard of experiencing a treatment interruption post-elevated VL; c) Time from Return to Repeat VL: Hazard of a repeat VL after returning to care; d) Time to Repeat VL: Hazard of a repeat VL following the initial elevated VL; e) Time to Suppressed VL: Hazard of achieving viral suppression after an initial elevated VL.

Abbreviations: VL, Viral Load; TLD, tenofovir disoproxil fumarate/lamivudine or emtricitabine/dolutegravir [TDF/XTC/DTG]; TLE, tenofovir disoproxil fumarate/lamivudine or emtricitabine/efavirenz [TDF/XTC/EFV]
