# Supplementary material for: Identifying care gaps along the HIV treatment failure cascade: A multistate analysis of viral load monitoring, re-suppression, and regimen switches in Zambia
Source: PLoS Med. 2025 Sep 3;22(9):e1004720. doi: 10.1371/journal.pmed.1004720 (PMC12422583; doi:10.1371/journal.pmed.1004720)
Supplement: S3 Fig — (DOCX) [file pmed.1004720.s009.docx]

**S3 Fig. Transition Hazards between States over Time after Due for Switch.** This figure presents five panels showing the instantaneous hazard rates over time for individuals on TLD (blue line) and TLE (red line) following a second elevated VL and being due for treatment switch. The x-axis in each panel represents time in days since the individual was due for a switch, and the y-axis represents the instantaneous hazard rate. a) Time to Return after Due for Switch: Hazard of returning to care after being due for a regimen switch. b) Time to Treatment Interruption after Due for Switch: Hazard of experiencing a treatment interruption post-switch eligibility. c) Time to Switch: Hazard of switching treatment regimens after being due for a switch. d) Time to Additional VL after Due for Switch: Hazard of receiving an additional elevated VL test after being due for a switch. e) Time to Suppressed VL after Due for Switch: Hazard of achieving viral suppression following switch eligibility.

Abbreviations VL, Viral Load; TLD, tenofovir disoproxil fumarate/lamivudine or emtricitabine/dolutegravir [TDF/XTC/DTG]; TLE, tenofovir disoproxil fumarate/lamivudine or emtricitabine/efavirenz [TDF/XTC/EFV]
