# Supplementary material for: Identifying care gaps along the HIV treatment failure cascade: A multistate analysis of viral load monitoring, re-suppression, and regimen switches in Zambia
Source: PLoS Med. 2025 Sep 3;22(9):e1004720. doi: 10.1371/journal.pmed.1004720 (PMC12422583; doi:10.1371/journal.pmed.1004720)
Supplement: Attachment — Submitted filename: Response to reviewers_Gaps in VL monitoring_02_07_2025.docx [file pmed.1004720.s013.docx]

July 2, 2025

Dear Editors,

We are pleased to submit our revised manuscript titled *“Defining care gaps along the HIV treatment failure cascade: a multi-state analysis examining gaps in viral load monitoring, resuppression, and regimen switches in Zambia”* for your consideration in *PLOS Medicine*. The reviewer queries have helped us to make the manuscript stronger, and we hope these responses address the concerns raised.

Our responses are captured below in red.

Thank you for your time and consideration.

Sincerely,

Kombatende Sikombe

**Academic Editor:**

Please expand the discussion a bit to stress the implications for other settings with a generalised epidemic. Please also add a discussion on possible causes of implementation gaps as may be suggested by your data.

Thank you for these comments. We have added to the discussion to better emphasize that these findings are broadly relevant beyond Zambia and provide insights for many public health HIV programs that use similar care algorithms and procedures and face similar care delivery challenges. This is captured in line 786 on page 20 and line 942-943 on page 24.

Additionally, we have expanded on the discussion some of the implementation gaps that occur once an individual returns to the clinic. Unfortunately, from our data, we are not able to provide definitive reasons as to why these occur, but we do offer potential plausible reasons based on our familiarity with the health system and provider behaviours (e.g., providers waiting to complete all EAC sessions, not checking VL if they know client not taking medications, or just being unaware that they should be checking VL).

**Response to Reviewers:**

Reviewer #1:

This manuscript examines implementation gaps in HIV service delivery in Zambia using a national electronic HIV record from 24 clinics. The strengths of this study include leveraging a national electronic database that goes down to local levels and detailed longitudinal data on individuals. This is an exciting manuscript that will be useful for those designing HIV services in LMICs.

MAJOR COMMENTS
1)      COVID-19 impact. The study duration includes periods both prior to and during the COVID-19 pandemic. While the extent of Zambian lockdown was less than many other countries, this could also plausibly impact what happens among PLHIV who are not virally suppressed. Based on the data from Figure S1, it does seem like there were some major changes in the distribution of individuals with an elevated viral load around that time. Would it be possible to do a sensitivity analysis that disaggregates the data based on before and after Dec 2020 (the second wave when 501.V2 variant substantially spread through Zambia)? Are the main findings robust when data are disaggregated this way? Data from Table S5 suggest that this is an important factor that could be incorporated more into the analysis.

We appreciate the reviewer’s comment regarding the potential impact of the COVID-19 pandemic on the study findings. In Zambia, the primary period of COVID-19 lockdown was between April 2020 and Sept 2020, during which stable individuals were given longer ART refill durations to minimize contact at the facility and maintain capacity for those who needed to be seen at the facility. After this period, COVID-19 restrictions were lifted and remained minimal even during the second wave in December 2020 (although outcomes were worse during that second wave). We do note the decrease in number of elevated VLs at this time the reviewer mentions in Figure S1, though do also want to highlight that some of this decrease is likely also due to the shifts in TLD, which would also likely decrease treatment failure.

In response, we did also conduct sensitivity analyses stratified by period (prior to April 2020, April 2020 – Oct 2020, and post Oct 2020). Given the temporal trends and scaling up of DTG, we also stratified each period by TLE vs. TLD. Results are shown in the table below.

| **After first Elevated VL** | | | | | | | |
| --- | --- | --- | --- | --- | --- | --- | --- |
| **Period of First Elevated VL** | **Pre-COVID**  (01 Aug 2019− 31 Mar 2020) | **COVID Lockdown**  (01 Apr 2020− 30 Sept 2020) | **Post-COVID Lockdown**  (01 Oct 2020− 30 Nov 2021) | **Period of First Elevated VL** | **Pre-COVID**  (01 Aug 2019− 31 Mar 2020) | **COVID Lockdown**  (01 Apr 2020− 30 Sept 2020) | **Post-COVID Lockdown**  (01 Oct 2020− 30 Nov 2021) |
| **TLD** | | | | **TLE** | | | |
| **Time (days)** | **Any visit** | | | **Time (days)** | **Any visit** | | |
| 90 | 41.1  (38.4-43.8) | 27.3  (24.7-29.8) | 26.5  (25.2-27.7) | 90 | 55.1  (53.4-56.7) | 25.6  (23.5-27.8) | 40.4  (37-43.7) |
| 180 | 74.9  (72.3-77.4) | 64.9  (62.1-68.1) | 71.1  (69.4-72.9) | 180 | 79.4  (78.1-80.7) | 66  (63.5-68.7) | 74.3  (71.1-77.5) |
| 365 | 90.5  (88.6-92.4) | 90.6  (88.3-92.7) | 87.9  (86.3-89.3) | 365 | 88.3  (87.3-89.4) | 87.7  (85.8-89.8) | 87.9  (85.2-90.5) |
| **Time (days)** | **Lost-to-follow-up** | | | **Time (days)** | **Lost-to-follow-up** | | |
| 90 | 2.3  (1.5-3) | 0.5  (0-0.8) | 0.7  (0.5-0.9) | 90 | 3  (2.5-3.5) | 0.9  (0.4-1.4) | 1.6  (0.7-2.5) |
| 180 | 14.4  (12.5-16.4) | 11.4  (9.6-13.2) | 9.1  (8.2-10) | 180 | 18.6  (17.4-19.8) | 12.6  (10.9-14.2) | 13.2  (10.8-15.3) |
| 365 | 14.7  (12.9-16.6) | 12.7  (10.7-14.7) | 14.7  (13.3-16.1) | 365 | 19.9  (18.7-21.1) | 13.7  (11.9-15.5) | 16.8  (14.1-19.7) |
| **Time (days)** | **VL checked** | | | **Time (days)** | **VL checked** | | |
| 90 | 6.9  (5.5-8.1) | 6.1  (4.7-7.5) | 6.2  (5.5-6.8) | 90 | 8.7  (7.8-9.6) | 7.2  (5.8-8.4) | 10.7  (8.6-12.7) |
| 180 | 34.9  (32.3-37.3) | 31  (28.4-33.5) | 35.6  (34.2-37.1) | 180 | 36.8  (35.2-38.2) | 37.7  (35.2-40.3) | 48.2  (45.2-51.6) |
| 365 | 68.7  (66.3-71.2) | 74.7  (72.1-77.2) | 67.4  (65.6-69.2) | 365 | 67.9  (66.5-69.2) | 76.5  (74.2-78.8) | 72.9  (69.7-76.2) |
| **Time (days)** | **Suppressed** | | | **Time (days)** | **Suppressed** | | |
| 90 | 4.4  (3.3-5.4) | 4.6  (3.3-5.8) | 4.7  (4-5.2) | 90 | 4.1  (3.4-4.6) | 3.2  (2.3-4) | 7.5  (5.6-9.2) |
| 180 | 29.2  (26.6-31.4) | 23.8  (21.3-26.2) | 30.8  (29.4-32.2) | 180 | 17.7  (16.5-18.9) | 21.7  (19.6-23.9) | 35.4  (32.5-38.6) |
| 365 | 57.1  (54.3-59.7) | 60.4  (57.3-63.4) | 59  (57.3-60.8) | 365 | 35.3  (33.8-36.7) | 49.5  (46.9-52) | 54.7  (51-58.1) |
| **Time (days)** | **Percent Suppressed** | | | **Time (days)** | **Percent Suppressed** | | |
| 90 | 64.2  (54.5-74.2) | 75  (64-85.7) | 75.5  (70.6-80.7) | 90 | 46.7  (41.6-51.7) | 44.8  (35-54.8) | 69.8  (60-78.4) |
| 180 | 83.7  (80.2-87.2) | 76.7  (72.4-81.3) | 86.5  (84.8-88) | 180 | 48.1  (45.5-50.8) | 57.7  (53.5-62) | 73.4  (69.2-77.6) |
| 365 | 83.1  (80.5-85.7) | 80.9  (77.9-83.5) | 87.6  (86.2-89) | 365 | 52  (50.1-53.8) | 64.7  (62-67.4) | 75  (71.6-78.4) |
| **After second Elevated VL** | | | | | | | |
| **Period of First Elevated VL** | **Pre-COVID**  (01 Aug 2019− 31 Mar 2020) | **COVID Lockdown**  (01 Apr 2020− 30 Sept 2020) | **Post-COVID Lockdown**  (01 Oct 2020− 30 Nov 2021) | **Period of First Elevated VL** | **Pre-COVID**  (01 Aug 2019− 31 Mar 2020) | **COVID Lockdown**  (01 Apr 2020− 30 Sept 2020) | **Post-COVID Lockdown**  (01 Oct 2020− 30 Nov 2021) |
| **TLD** | | | | **TLE** | | | |
| **Time (days)** | **Any visit** | | | **Time (days)** | **Any visit** | | |
| 90 | 80.6  (70-92.9) | 42.6  (32.4-51.9) | 60  (55.6-64.5) | 90 | 82.4  (79.4-85.4) | 50.6  (46.7-54.5) | 63.5  (60.1-67.1) |
| 180 | 92.5  (81.2-100) | 85  (72.4-94.3) | 88.4  (84.1-92.3) | 180 | 91.7  (89.4-93.6) | 88.9  (85.1-91.8) | 89.7  (87.2-92) |
| 365 | 97.7  (92.6-100) | 97.4  (93.4-100) | 97.2  (94.9-98.9) | 365 | 95.6  (93.9-96.9) | 95.4  (92.3-97.6) | 95.9  (93.9-97.6) |
| **Time (days)** | **Lost-to-follow-up** | | | **Time (days)** | **Lost-to-follow-up** | | |
| 90 | 0  (0-0) | 1.9  (0-3.6) | 3  (1.3-4.5) | 90 | 2.9  (1.4-4.2) | 0.8  (0-1.3) | 1.9  (0.8-2.9) |
| 180 | 6.5  (0-12.5) | 18.5  (10-26.7) | 16.1  (12.3-19.5) | 180 | 13  (10.1-15.6) | 9.5  (7.3-11.7) | 8.8  (6.6-11) |
| 365 | 6.5  (0-12.5) | 14.8  (6.7-22.2) | 20.1  (15.1-24.9) | 365 | 13.3  (10.5-16.1) | 11.1  (8.7-13.2) | 9.2  (6.8-11.9) |
| **Time (days)** | **Switched regimens** | | | **Time (days)** | **Switched regimens** | | |
| 90 | 25.8  (13.3-37.5) | 13  (5.9-19.4) | 16.8  (13.2-20) | 90 | 31  (27.1-34.9) | 26.7  (23.6-30.1) | 36.4  (32.9-39.9) |
| 180 | 35.5  (20-50) | 22.2  (13.3-31.2) | 23  (19.3-27.1) | 180 | 49  (44.7-52.9) | 54.8  (51.2-58.7) | 51  (47.3-54.8) |
| 365 | 49.5  (35-63.9) | 27.8  (18.4-37.5) | 23  (19.3-27.1) | 365 | 65.8  (61.9-69.5) | 73.8  (70.7-77) | 59.1  (55-63) |
| **Time (days)** | **VL checked** | | | **Time (days)** | **VL checked** | | |
| 90 | 12.9  (4.5-21.4) | 11.1  (5-17.1) | 7.7  (5.1-10) | 90 | 7.7  (5.4-9.8) | 5.9  (4.1-7.7) | 6.8  (5-8.5) |
| 180 | 38.7  (25-52.9) | 29.6  (21.2-38.9) | 33.2  (28.5-38) | 180 | 20.6  (17.2-23.8) | 22.4  (19.4-25.5) | 24.7  (21.5-27.9) |
| 365 | 50.8  (36.1-65.1) | 50  (39.3-60.6) | 52.2  (45-58.6) | 365 | 31.2  (27.2-34.9) | 31.4  (27.9-34.8) | 33.3  (29.7-37.4) |
| **Time (days)** | **Suppressed** | | | **Time (days)** | **Suppressed** | | |
| 90 | 6.5  (0-12.5) | 9.3  (3-15.6) | 4.6  (2.7-6.3) | 90 | 1.5  (0.5-2.4) | 1.3  (0.4-2.1) | 3.6  (2.2-5) |
| 180 | 25.8  (13.3-38.1) | 22.2  (12.9-30.8) | 22.9  (18.6-26.6) | 180 | 6.5  (4.3-8.3) | 5.9  (4.1-7.6) | 15.7  (12.8-18.4) |
| 365 | 32.3  (18.7-46.2) | 40.7  (30-50) | 40.5  (34.2-47.2) | 365 | 10.6  (8-13) | 11.1  (8.7-13.2) | 24.1  (20.2-27.5) |
| **Time (days)** | **Percent Suppressed** | | | **Time (days)** | **Percent Suppressed** | | |
| 90 | 50  (0-100) | 83.3  (50-100) | 59.9  (43.4-73.8) | 90 | 19.2  (6.2-30.8) | 17.4  (6.2-28.6) | 53.9  (38.8-68.6) |
| 180 | 66.7  (42.9-87.5) | 75  (57.1-90.9) | 66.6  (58.7-74) | 180 | 30  (21.9-38.1) | 25.3  (17.6-32.5) | 61.6  (54.2-68.9) |
| 365 | 63.5  (45-87.5) | 77.8  (66.6-91.7) | 70.9  (63-78.6) | 365 | 31.2  (24.7-37.7) | 31.2  (24.5-37.3) | 62.6  (55.9-69.6) |

Based on these results, there do not appear to be any clear or consistent trends across time periods, except for potentially decreased early return (i.e., within 90 days) and increased suppression among those who initially failed on TLE. It is unclear whether these trends are due to responses to COVID or more general secular trends that were occurring in the HIV treatment program over time (e.g., increasing duration of ART, increased routine switches from TLE to TLD due to programmatic scale-up). A caveat in interpreting these data is that individuals were categorized to a time period at the time of their first elevated VL, but they do have potential to cross-over into other time periods (e.g., an individual could start in pre-COVID period but then have care affected by COVID 90 or 180 days into in their observation time). We did not censor at cross over for this sensitivity analysis as this would greatly minimize observation time available (i.e., <6 months).

Given the added complexity of these data (in an already complex manuscript) and limitations in interpretability, our inclination was to not include this in the supplementary material. We are not sure that it adds much value to regression analyses presented in Table S5, which points to similar insights and promotes a fairly exploratory interpretation. However, we defer to the reviewers and editors and would be happy to consider including it if they do feel it still adds value to the manuscript without adding too much additional complexity.

2)      Health professional-level data. Within health systems, it would also seem that health professional level issues would be critical here. Does the electronic health system record include some indication of the health professional who assessed the patient? I assume that this granular information is not included in the data. If this is the case, it might be worth adding to the limitations.

We thank the reviewer for raising this important point. The electronic health record system used in this study does not capture detailed, consistent information about the specific health professional (e.g., cadre, experience level, or individual identifier) who assessed each patient, although we very much agree this would be highly valuable information. As such, we are unable to evaluate provider-level influences on outcomes such as viral load suppression. We agree that this is a limitation, as provider-level variation may play a role in the quality of care received.

We have now added this point to the limitations section of the manuscript (page 23, line 902-904), noting the absence of health professional-level data and its potential implications for interpreting the findings.

*“Our analysis is also limited by the absence of the ability to link client visits to a particular provider in the EHR, which precludes us from examining the role of particularly providers and their behaviours on client outcomes.”*

MINOR COMMENTS
1)      Abstract results section. Some of the estimates are have a range (eg, 11.4% (10.3 - 12.4)) without stating if the range represents a 95% CI or something else. Would be good to clarify these ranges.

We thank the reviewer for pointing this out. We have revised the results section of the abstract to clearly state that all ranges in parentheses represent 95% confidence intervals. For clarity, we have also added the notation “95% CI” at first use when presenting an estimate with a range.

2)      Introduction, first sentence. Consider condensing this sentence or separating it into two sentences.

Thank you for the suggestion to amend the opening sentence. We have edited this to read the following in line 214 on page 5

*“As HIV programmes have scaled up and access to antiretroviral therapy (ART) improved, sustaining viral suppression and improving outcomes among individuals with viremia has become a critical priority”*

3)      Missing VL group. The 20% of people who are missing a viral load is substantial and likely overlaps with treatment failure. Could these individuals have received a viral load at a clinic outside of the 24 included in this study? Is there another national database that could be used to track these people? My guess is "no", but interested to hear if there are other ways to capture this.

Thank you for this comment. As the reviewer notes, it is possible that these individuals who did not have a VL documented in the EHR may have transferred to another clinic and received a VL there or even had a VL performed at the same clinic that did not end up documented in the EHR. Given limitations in the interoperability of routine health information systems across facilities in Zambia, we are unable to link records from other clinics or databases reliably, and we do discuss the limitations of the EHR system in the limitation section. At present, there is no centralized national database that consolidates patient-level viral load data across all facilities in a way that would allow for complete longitudinal tracking, as there is in South Africa for example. Although some of the individuals with missing VLs may also have treatment failure, based on our experience using the SmartCare database, we do anticipate that our sample is still likely representative of those with treatment failure. Additionally, our team currently does have a large ongoing study to trace a random sample of individuals who are considered lost to follow-up or having missing VL data to better understand outcomes amongst these populations.

Reviewer #2: To authors:
I want to congratulate the authors on some fine work. This was a fascinating, relevant, and very well written manuscript. I really enjoyed reading it. I have just a few minor questions and suggestions - mainly things to clarify some sections that were a bit hard to follow in Methods and thoughts on relevant literature for the discussion. I hope they are helpful to the authors.  

Specific Comments:

Abstract

1.      A minor note that it seems a bit odd to mix up tenses (present tense in Background, but past tense in Methods). I'd suggest being consistent and using past tense throughout.

We thank the reviewer for pointing this out. We have changed the appropriate sentences in line 42 of the abstract Background to match the Methods section.

2.      In Results section of the abstract, can the authors add what proportion of total ART patients were represented by the individuals with an elevated VL and at how long since ART initiation they were (median months, for instance)? This would be useful info for context.

We have added the following sentence to the Results section in line 55-60 to include the total proportion represented and also median time on ART.

*“Among 179,855 individuals on ART (143,857 [80.0%] with documented VL), 7,916 (4.4%) had a documented elevated VL and drug regimen at time of treatment failure (52.3% female; median age 36.7 years [IQR 29.9-43.6], median time on ART 3.3 years [IQR 1.7-6.6], 54.6% on TLD and 45.4% on TLE) and included in the analysis (2,842 [2.0%] with elevated VL excluded for no documented drug regimen).”*

3.      In Results, the 72.2% who returned to clinic within 6 months of initial treatment failure. Is this when the treatment failure was documented, or when the individuals learned of their treatment failure?

We thank the reviewers for opportunity to clarify. This represents from the time the VL was collected. Unfortunately, the day the VL resulted and when or if a client was successfully contacted is not available in the EHR. We have rewritten the sentence on page 2, line 59-60 to hopefully be clearer.

*“Among those with treatment failure, only 72.2% (95% Confidence Interval [CI] 71.3-73.0%) had returned to the clinic 6-months after initial elevated VL was drawn.”*

Methods 

4.      Page 5: in the first paragraph of the Study population and setting section, could the authors add a brief description of the HIV care environment in Lusaka - how many individuals estimated to be HIV+, how many on ART, and the care cascade (proportion that know their HIV status; proportion on ART; proportion with suppressed VL)? The Zambia HIV epidemic is presumably a generalized epidemic but are there other trends underway that characterize the epidemic in Lusaka or Zambia more broadly (e.g. any particularly vulnerable populations)? This is important background context for the study.

We have added the following details about the HIV treatment programme in Lusaka province.

*“Lusaka province in Zambia has an HIV prevalence of 14.4% with estimates suggesting 89.7% diagnosed, 97.9% on treatment, and 85.3% suppressed within the province.”*

5.      Page 5, last line - what determines when the repeat VL should be between 3 and 6 months? That is, on what basis is the timing of the repeat VL determined?

We thank the reviewers for raising this important point. The guideline recommends that after an elevated VL, an individual undergoes 3 sessions of enhanced adherence counseling, typically at 2- to 4-week intervals. After completing EAC, which should happen within 3 to 6 months, an individual is supposed to get their VL rechecked. There is no clear determinant of whether an individual should get a VL rechecked at 3 vs 6 months, rather a recommended range is provided to account for uncertainties and inconsistencies in VL results returning and scheduled EAC appointments. We have made a change to this sentence to make it clearer that completion of EAC sessions determines the repeat VL after 3-6 months. This has now been captured on page 7, line 441-443.

*“Individuals with an elevated VL undergo 3 sessions of EAC, typically at 2- to 4-week intervals, and, after completion, should have their VL repeated within 3 to 6 months after the initial elevated VL.”*

6.      On page 6-7, lines 134-137, this statement is hard to follow: "We accounted for transitions between the various care states that occur prior to having a subsequent VL measure repeated…. (e.g. ….. repeat VL suppressed versus unsuppressed." It's unclear how one can account for transitions prior to the next VL, where transitions include the repeated VL. Can you explain this?

Thank you for noting this inconsistency and the opportunity to correct. This sentence should read “up until a subsequent VL was repeated” as the analysis accounted for all transition prior to and including when a VL was repeated. We have made the following edits:

*“We tracked transitions between the various care states that occur over time up until a subsequent VL measure was repeated per guideline recommendations (e.g., return visits without repeat VL, treatment interruption [>60 days late for scheduled visit], repeat VL suppressed versus unsuppressed) [18].”*

7.      Page 7, lines 139-144: this reads as if the 9 states are mutually exclusive throughout the period of observation (e.g. that if a client was in category 2 or 3, they would have no treatment interruption). But figure 1 - both text and figure itself (which is quite a work of art) and the later text on page 8 clarifies that categorization could happen twice, with a total of 19 possible categories for each client. To avoid confusion when reading the first part of this (on page 7, lines 139-144—which I re-read at least 3 times to try to understand how a total of 9 categories could make sense), I think it would help the reader if the text stated this more clearly, e.g. "In the first stage of categorization, clients were categorized into one of 9 …." Then readers know that more is coming in the next stage of categorization (with many more than 9 total categories used).

Thank you for this helpful suggestion. We have revised the manuscript on page 8 to refer to a first stage analysis of individuals starting with an elevated VL and a second stage analysis among those with two consecutive elevated VLs who are due for switch.

*“In the first stage analysis, individuals were categorized into one of 9 mutually exclusive and exhaustive states based on their clinical status at each time point after cohort entry”*

8.      Page 7, line 147 - what is the Markov assumption being referred to here? That clients could move between nonabsorbable states an unlimited number of times? That sentence is a bit confusing.

Thank you for opportunity to clarify. The Markov assumption states that, once entering a care state, an individuals’ prior history does not impact their probability of transitioning to subsequent states. That is, they have the same probability of transition as everyone else entering that care state, regardless of prior history. This assumption is necessary as it is not possible to adjust for every individual’s unique care history except through how each state is defined from the beginning. We have included the following clarification on page 8 in line 492.

*“Individuals could transition between nonabsorbable states an unlimited number of times based on the Markov assumption (i.e., after entering a state, prior states do not impact subsequent outcomes) until transitioning into an absorbable state (i.e., repeat viral load measure, transfer, death) or being censored [25].”*

Results

9.      Page 13, line 243: it is not clear what this means "… and median time from returning to having VL repeated was 63 days." Does it mean median time between returning to clinic after the initial elevated VL and the actual repeated VL test was 63 days? E.g., this is the lag between first return to clinic and having VL test repeated?

Thank you for the opportunity to clarify. The reviewer is correct that this represents the median time from the time an individual first returns to the clinic to the time they had their VL repeated (among those that had a VL repeated). We have made a slight change to line 647 on page 14.

Discussion

10.     Page 19, paragraph beginning in line 350: the association found between longer appointment duration after initial treatment failure and longer time to return to clinic is notable. The authors stress that care delivery needs to be responsive and flexible in contacting patients with elevated VL and providing them with support to improve adherence and increase likelihood of viral suppression. The authors may be interested in a clinical trial underway in South Africa that is addressing just this issue by monitoring individuals who have just initiated ART (using 3 different methods, including contact immediately following an elevated VL) and giving them support (see Jennings et al, "Protocol for an evaluation of adherence monitoring and support interventions among people initiating antiretroviral therapy in Cape Town, South Africa - A multiphase optimization strategy (MOST) approach using a fractional factorial design." Trials. 2023.)

We thank the reviewers for sharing this article and have since cited it in our discussion of the types of strategies that are needed.

11.     The authors do a nice job with their second discussion point highlighting the common experience of treatment gaps and the many barriers to retention in care, as well as the role providers may play in discouraging individuals from returning to care to recommence treatment. For related evidence gathered from community clinics in nearby Cape Town, see Killian C, et al, "Negative clinic experiences as a barrier to care for people with HIV and their impact on patient preferences for intervention support: A qualitative study in Cape Town, South Africa." AIDS Care. 2024.

We thank the reviewers for highlighting this important work from community clinics that echoes similar findings from our group in Zambia. We have included this refence in our discussion

Reviewer #3: Overall

Thank you for the opportunity to review this well written manuscript of outcomes after viraemia in routine care in Zambia. This is a comprehensive and generally well conducted set of analyses. However, the findings are not particularly novel, and so I question whether this would meet the PLoS Medicine threshold of having "potential to directly and substantially inform clinical practice or health policy" - there are no new recommendations arising that are not already known/in place as far as I could see.

We appreciate the reviewer’s thoughtful comment on our manuscript and the perceived novelty of its findings. While it is true that global guidelines exist for managing virologic failure, our study offers novel empirical evidence on how these guidelines function or fall short in real-world, routine care settings. Specifically, we believe our use of novel multistate methods to comprehensively characterize the treatment failure cascade under routine conditions, including return visits, treatment interruptions, repeat VLs (or lack thereof), and switching (including provider switching behaviours), helps draw attention to and quantify the multiple and compounding gaps that impede timely viral resuppression. As most studies to date have focused on rates of resuppression among those who repeat viral load testing, we believe the use of sophisticated methods to describe key service delivery outcomes and implementation gaps is an important addition to the existing literature and highlights challenges that are often less prioritized and yet to be addressed even in the modern era of TLD. These gaps reflect not only client-side challenges, such as retention and engagement in care, but also systemic and provider-level factors, such as inconsistent application of guideline-based decision-making, missed opportunities for timely viral load rechecks, and delays in switching to second-line therapy.

MINOR

ABSTRACT

INTRODUCTION

Line 65-66: Resuppression rates on dolutegravir likely differ from EFV which was likely the dominant anchor drug in the studies summarised in the two cited papers

We thank the reviewer for this valuable observation. As correctly noted, the studies cited (e.g., Ford et al.) largely reflect populations on TLE, with efavirenz as the anchor drug. We agree that resuppression dynamics likely differ for patients on dolutegravir-based regimens (TLD), as this and other studies have shown, given its higher genetic barrier to resistance and different pharmacological profile. We have clarified this distinction in the revised text on page 5, line 216-218 and updated our references to better reflect the evolving evidence base on TLD.

*“Existing evidence suggests 50-70% of individuals with treatment failure (i.e., VL >=1000 copies/ml) have documented resuppression after receiving enhanced adherence counselling (EAC) for 3 to 6 months across Africa, though resuppression rates are likely higher among those on integrase-based regimens.”*

Line 92: Wouldn't the transition to DTG also effect resuppression due to lack of drug resistance i.e. virological response to improved adherence?

We thank the reviewer for highlighting this important point. Some of the main benefits of our approach over existing literature is that it provides a more granular depiction of care patterns and client/provider behaviour, which is why we had initially focused on highlighting those. However, the reviewer is indeed correct that the clinical differences between regimens is also quite important (i.e., increased barrier to resistance, improved biologic efficacy). We have edited the sentence on page 6, line 382, to be more comprehensive.

*“This approach also helps assess whether the transition from TLE to TLD has influenced care patterns, provider behaviours, and outcomes.”*

Line 98-99: But no assessment of whether EAC was done or not?

We thank the reviewer for this comment. As we discuss in the manuscript and limitations, we were unable to specifically identify when/whether EAC was initiated and only had data on visits made to the clinic. In response, we make more explicit that we use return clinic visits as a proxy for EAC initiation given limitation of the underlying data. We made the following edits to this sentence on page 6.

*“Using data from 24 public HIV health facilities in Lusaka, Zambia, we examine the longitudinal dynamics of clients’ care engagement after treatment failure with respect to return clinic visits (as a proxy for EAC initiation) and retention in care, repeat VL monitoring, switch to second-line therapy, rates of re-suppression, and how these differ on TLE versus TLD [18,21].”*

METHODS

Line 100: What is the threshold for elevated VL? 50 cps/ml, 200, 1000?

The VL threshold for treatment failure and to initiate EAC according the Zambian guidelines is ≥1000 copies/ml. We clarify that the viral load threshold in the Methods section (page 6, line 397), where we state:

*“We assessed guideline concordance of VL monitoring and switch to second-line ART regimens among adults aged 18 years or older who had their first documented elevated VL (≥1000 copies/ml) between 1 August 2019 and 31 November 2021 after being on ART for at least 180 days* and had a documented first-line drug regimen at the time of treatment failure*.”*

Line 128: Are there any published studies or other evidence which have evaluated data capture completeness in this dataset, to help understand how much of treatment interruption or lack of repeat VLS could be due to data capture issues?

We appreciate the reviewer’s question regarding data capture completeness. There are indeed published studies that provide insight into the quality and limitations of the electronic health record system used in Zambia. Moomba et al 2020 <https://doi.org/10.5588/pha.19.0068>, evaluated the impact of transitioning from paper-based (electronic-last) to point-of-care electronic data entry (electronic-first). This assessment found pharmacy dispensing data to be over 90% complete following the transition, which supports confidence in the treatment continuation data used in our study. However, it also revealed that some variables, including enhanced adherence counselling documentation and repeat VL results remain inconsistently captured.

Most notably, Holmes et al., (2018) and Sikazwe et al., (2019) conducted a sampling-based tracing study that directly addressed data misclassification among patients categorized as lost to follow-up (LTFU). By tracing a representative sample of patients recorded as LTFU in the EHR, the study found that a significant proportion had actually died or silently transferred to other facilities, underscoring the limitations of routine data in accurately capturing outcomes. These studies provided robust empirical evidence on true mortality and disengagement rates using a multistage, sampling-based tracing approach. They served as a critical piece of validation for electronic health record data and helps quantify the extent to which routine data may under-report mortality or LTFU outcomes.

We have expanded on this limitation in the discussion section and have now included the Moomba et al. reference in the discussion.

Line 137: This sentence focusses on states before repeat VL is done, but then includes repeat VL suppressed/unsuppressed

We thank the reviewers for raising this important point, which was also noted by another reviewer.
We have revised the text on page 8, lines 498–501 for clarity.

The updated sentence now reads:

*“We tracked transitions between the various care states that occur over time up until a subsequent VL measure was repeated per guideline recommendations (e.g., return visits without repeat VL, treatment interruption [>60 days late for scheduled visit], repeat VL suppressed versus unsuppressed) [18].”*

Line 141: I would question the assumption that people remain viraemic just because they haven't had a visit. In our experience in South Africa, many people with viraemia have disengaged (though maybe this isn't apparent to the HCW) and the VL is taken on the day they re-engage with care, and so they likely have restarted ART and so may be suppressing

We appreciate this important point and agree that the labelling care states may have implied that an individual remained viraemic until they entered another care state, which is not appropriate. Indeed, initial care state is defined by an individual having an elevated VL, but it is not possible to say how their viral load changes after that measurement. To be clear, an individual falling into a particular care state only indicates that they have met the criteria to enter it and have not yet met any criteria to another care state (in this case, they have had an initial elevated VL but they have yet to return for a visit, meet the definition for treatment interruption, or have a repeat VL). To minimize confusion and inappropriate interpretation, we have relabelled the care states as “Treatment Failure, Not Returned” and “Treatment Failure, due for switch” throughout the manuscript.

Line 199: "Independently associated" - its stated that DAGs were used to determine what risk factors to include in multivariable Cox models, but then it appears in table S5 that there is just one large multivariable model with all the variables thrown in, and also 3 different outcomes with the same covariates in the model. Ideally (to avoid "Table 2 Fallacy") a model should be built to assess the relationship between a specific exposure and specific outcome, with potential confounders selected on whether they might confound that specific relationship. So maybe rephrasing the aim of this analysis is needed.

Thank you for bringing up this important point. The reviewer is indeed correct that these are exploratory analyses, and not strictly designed to assess the relationship between a single exposure and outcome. Our use of DAGs was primarily to ensure that we discussed any findings with the correct lens or interpretation, rather than to identify specific confounders and mediators to include/exclude in an adjustment set. We have now rephrased the aim of the analysis in the methods section to reflect this exploratory intent and have removed the term “independently associated” to avoid implying causal interpretation of coefficients. The change is captured on page 10, line 600.

*“We also used Cox proportional-hazards models to examine associations between baseline sociodemographic and clinical characteristics and outcomes of interest: time to repeat VL and resuppression after initial treatment failure as well as time to second-line switch or time to resuppression prior to switch after two consecutive elevated VLs (i.e., due for regimen switch). We accounted for within facility correlation by including facility as a fixed effect. We used multiple imputation with chained equations (n=20 imputations) to account for missingness in sex, age, World Health Organisation (WHO) stage, marital, and education status. We using directed acyclic graphs based on a priori hypotheses of causal relationships to interpret results based on whether covariates should be considered confounders or mediators.”*

Line 199: How was correlation within facilities accounted for in the Cox models?

We accounted for within-facility correlation by including facility as a fixed effect. This has been captured in line 603 on page 10.

RESULTS
Lines 239-250: Would be nice to see n/N or at least the denominator for the percentages presented. E.g. are the 50% with resuppression at 12 months 50% of those who had VL done, or 50% of cohort?

AND

Line 278: Again, would be nice to know the n/N here to know how many people in the TLD group and how many on TLD were switched to second-line - seems from the next paragraph that it was very few.

We appreciate this comment and agree that clarity around denominators is important. However, as these estimates are based on a time-to-event analysis, it is not actually possible to provide estimated proportions as an n/N. We do note, however, that Table S1 does provide the actual frequency of transitions, although it is not possible to directly translate these numbers to estimated proportions. In response, we do also now include the total Ns for each cohort in the figures, themselves (previously only listed in Figure legend). We also have gone through the results to ensure that it is clear among which population each estimated proportion is from. We noted that our use of “Overall” could be interpreted as referring to two different groups: the Overall cohort (as opposed to restricting among those who return or with repeat VL, etc.) and overall, as in individuals on TLE and TLD combined. We have tried to make edits to increase clarity.

For example, we can clarify that the 50.1% resuppression estimate at 12 months reflects the cumulative incidence among the full cohort that entered the analysis at time zero (i.e., those with a first elevated VL). We now state

*“In the overall cohort, 50.1% (48.9-51.3%) of individuals had documented resuppression at 12 months.”*

For the total number of individuals on TLD who were ultimately switched, we have now stratified Table S1 by TLE and TLD as well so these numbers are more apparent. As the reviewer notes, this information is also presented in the subsequent paragraph.

Line 278: "Among those on TLD at 6 months, …" its not clear to me why this is at 6 months. Should it not be among those on TLD at second repeat VL (ie at entry into this 'eligible for switch' cohort". - On re-reading I think this should read "Among those on TLD, at 6 months…."

We thank the reviewer for pointing out this punctuation error. The reviewer is indeed correct in their interpretation. The correction has now been made in the revised manuscript on page 16 line 718.

Line 304:  I wouldn't classify switching from TLD to AZT/XTC/DTG as a change to second-line ART, there is no change in anchor drug. Could this have been a switch due to renal toxicity?

Thank you for this comment. Although we agree that this clinically would not be an appropriate second-line regimen, we do believe this switch was likely intended to be in response to individuals’ treatment failure for several reasons and have thus interpreted it as an inappropriate switch. First, although only PI-based regimens were formally listed as second-line regimens at the time (for both TLE and TLD failure), AZT/3TC/DTG was informally used as well (as is clearly demonstrated in our findings). Formal guidance for using AZT/3TC/DTG as second-line for TLE failure only was only released in the 2022 guidelines after the study period. We now make this fact in clear in the methods and discussion. Second, this switch was the second most common among individuals on TLD with two consecutive viral loads (even though clinically inappropriate) and most common or individuals on TLE. Lastly, in response to this comment, we did examine the available creatinine data. Among the 35 on TLD that switched to AZT/3TC/DTG, only 20 had an available serum creatinine. Only 6 of these were done in the past year, and only 2/20 were above 1.2mg/dL (both done >2 years prior to the switch). Thus, it is unlikely this switch was due to renal toxicity.

Line 307: Are the results post switch presented for the whole cohort? It would be nice to see them by TLD versus TLE too. 

Thank you for this comment. We now include stratified estimates of suppression post-switch for those on TLE vs. TLD first line as well. We do not provide additional granularity given the relatively small numbers.

*“At 6-months after switch, 54.3% (CI 51.5-57.2%) had a VL rechecked overall and this increased to 80.7% (CI 78.3-83.3%) by 1-year; 83.0% (CI 80.7-85.3%) of those who had VL rechecked by 1 year were suppressed (75.4% [CI 66.6-84.8%] on TLD first-line vs. 83.7% [CI 81.4-86.0%] on TLE first-line).”*

Line 317: "having a longer appointment duration after initial treatment failure was highly associated with longer time to return" - This seems obvious - longer intervals means people will come back later? Or am I misunderstanding? 

We appreciate this observation. While it may seem intuitive that longer scheduled intervals would lead to later return visits, this finding is notable in the context of clinical guidelines which recommend prompt follow-up after an elevated VL. In theory, facilities should proactively shorten appointment intervals and intensify follow-up for patients with suspected treatment failure. The association we observed suggests limited adaptation of scheduling practices in response to elevated VL, indicating a missed opportunity to re-engage clients promptly. We have made a change in line 770-771, 815 which now reads

*“being scheduled for a longer appointment interval at the time initial elevated VL was drawn”*

DISCUSSION 

In general, the discussion is quite long and in places makes quite broad recommendations that go slightly beyond the findings of the presented analyses. 

Line 350: "lack of prompt initiation of EAC" - but this study did not look at when EAC was initiated…

Thank you for this helpful clarification. We agree that our analysis did not directly measure the timing of EAC initiation. In the absence of consistently documented EAC dates in the dataset, we used the timing of return visits after an initial elevated viral load as a proxy for EAC initiation. Thus, we do believe it is reasonable to interpret our finding of lack of prompt return as a proxy for lack of prompt EAC initiation. We have now made this connection between return visits and EAC initiation more explicitly in the Methods section on page 8 line 492 to 493 and acknowledge this limitation in our study discussion. We also make the following revision:

*“First, a lack of prompt return (and thus likely initiation of EAC) and timely assessment of response represents a major gap.”*

Line 353: The emphasis has been placed on strong relationship between appointment duration and time to repeat VL, whereas in the results emphasis is on relationship with outcome of time to repeat clinic visit.

Thank you for pointing this out. We have revised the text in the discussion to better align with the interpretation of findings, and make the following edits on page 20, line 815.

“*The length of the scheduled appointment interval at the time of elevated VL was highly associated with longer time to return, indicating a limited adaptation to care delivery once treatment failure is identified.”*

Line 369: "In our study, approximately 30% of individuals had been 30 days late for an appointment at least once after treatment failure, and 59.2% of those who did not have a VL rechecked by 1 year (and had not died or transferred) were in a treatment interruption" Sorry if I missed it but don't recall these being presented in the results.

Thank you for raising this issue. We now include these results on page 14, line 668-670.

*“At 12-months, 16.6% (CI 15.9-17.2%) of individuals were currently experiencing a treatment interruption and 32.5% (CI 31.8-33.3%) had ever experienced treatment interruption since treatment failure. Additionally, 59.3% (CI 57.5-61.0%) of those who did not have a VL rechecked by 1 year were in a treatment interruption (Figure 3A, Table S2, Figure S2).”*

And wasn't the treatment interruption definition >60 days?

We thank the reviewers for pointing out this typo as it should read 60 days.

Line 394: "Our study results also provide important new insights into outcomes after treatment failure during routine care as programs shift from TLE to TLD.." There are several published papers looking at this

<https://academic.oup.com/ofid/article/10/12/ofad583/7424682>
<https://academic.oup.com/ofid/article/11/2/ofae013/7512841>
<https://journals.lww.com/jaids/fulltext/2024/04150/viremia_and_hiv_drug_resistance_among_people.14.aspx>
<https://journals.lww.com/aidsonline/fulltext/2024/09010/longitudinal_viral_load_outcomes_of_adults_with.12.aspx>

We thank the reviewers for the above references, which we have incorporated into the introduction and discussion. We also temper the language in the discussion as follows as we agree that some of the specific findings about TLD vs TLE have been previously reported:

*“Our study results also provide additional insights into outcomes after treatment…”*

Line 399: "Assuming similar levels of adherence" - could also be due to less side effects with DTG? Especially low level side effects that could tip people over the edge of not taking treatment regularly

Thank you for this comment. Although it does seem plausible that adherence could differ due to different side effect profiles of efavirenz vs. dolutegravir, we are not familiar with any evidence or published manuscripts that have clearly demonstrated a difference in adherence between these medications. Indeed, we found several the suggested the differences, if any, might be minimal despite concerns of the neuropsychiatric side effects of efavirenz. Thus, we have made the following edits suggesting that the assumption is plausible although not guaranteed. We are open to further changes if the editors or reviewer are aware of evidence that we have missed that really question the plausibility of this assumption.

*“Assuming similar levels of adherence, which is plausible although not guaranteed, this difference likely represents the gap in acquired resistance that frequently occurs on TLE but rarely on TLD.”*

Line 409: What were the Zambian guidelines regarding components of the second line regimen after failure on TLD?

Thank you for this comment. The guidelines at the time recommended switching to a PI (for both NNRTI and INSTI failure) and switching TDF to AZT. There was no formal recommendation for use of INSTI-based second line, though informally this was commonly done (as is evidenced in our study). Formal guidance to include INSTI-based second line regimens for TLE failure were only released in 2022, after the study period. We have included these details more clearly in the Methods and Discussion as follows:

*“In our study, 45% of those who had switched regimens on TLD were likely switched to an inappropriate regimen (i.e., 37 % only had TDF changed to AZT, 8% switched to NNRTI-based regimen), although it is also worth noting that a small proportion of individuals on TLD required regimen switches and that formal guidance on use of integrase-based second line regimens only after TLE failure were not issued until after the study period in 2022.”*

Line 439: "driven by delays in returning for adherence counselling" this seems to place the blame on the client as they didn't return, rather than the healthcare system which may not have recalled them promptly

We agree with the reviewer that the original phrasing may unintentionally imply client blame, rather than reflecting the shared responsibility between clients and the health system. We have made the following edits:

*“There are substantial gaps along the treatment failure cascade precluding viral re-suppression, driven by delays in outreach, return for adherence counselling, and repeat viral load monitoring to assess response; treatment interruptions; and missed opportunities in rechecking viral load status or switching to second-line therapy in routine care in Zambia.”*

TABLES AND FIGURES

Table 1: 

The rows for ART regimen are not necessary as represented in columns?

Thank you for the suggestion. We opted to keep the row for ART regimen so the percentages are clear in the Overall column, although we do agree that there is some redundancy to the information

Would be nice to see time on current regimen at first elevated VL

Thank you for the suggestion. Unfortunately, particularly during early time periods (e.g., prior to 2020), regimens were not always captured with completeness. Thus, it is not always possible to reliably capture the time on a particular regimen. Although some assumptions can be made about individuals initiating TLE if ART was started >10 years ago and regimen is missing, it is not always apparent in more recent times when they also could have been initiated on TLD or switched to it. As such, we believe it is better to maintain time on ART rather than time on particular regimen.

Fig 3:A
- Same comment as above re problems with assuming those that haven't returned are still viraemic

Thank you for the comment. We have changed the label names as per our previous response for the care states to “Treatment Failure…” which we think is less likely to imply that it is known whether an individual remains viremic (which is not possible).

Fig 4: It would be nice to see the N numbers for each figure (esp TLD and TLE)

Thank you for the suggestion. We have now done this.
