# Supplementary material for: Identifying care gaps along the HIV treatment failure cascade: A multistate analysis of viral load monitoring, re-suppression, and regimen switches in Zambia
Source: PLoS Med. 2025 Sep 3;22(9):e1004720. doi: 10.1371/journal.pmed.1004720 (PMC12422583; doi:10.1371/journal.pmed.1004720)
Supplement: Attachment — Submitted filename: Response to reviewers_Gaps in VL monitoring_07_08_2025.docx [file pmed.1004720.s014.docx]

August 7, 2025

Dear Editors,

We are pleased to submit our revised manuscript titled *“Identifying care gaps along the HIV treatment failure cascade: a multi-state analysis of viral load monitoring, re-suppression, and regimen switches in Zambia”* for your consideration in *PLOS Medicine*. The reviewer queries have helped us to make the manuscript stronger, and we hope these responses address the concerns raised.

Our responses are captured below in red.

Thank you for your time and consideration.

Sincerely,

Kombatende Sikombe

PLOS Medicine
plosmedicine.org

------------------------------------------------------------
Requests from Editors:

Please revise the title to comply with PLOS Medicine style: e.g. Identifying gaps in HIV viral load monitoring, resusppression and regimen switches in Zambia: a multi-state analysis OR Identifying drivers of HIV treatment failure in Zambia: a multi-state analysis.
Your title must be nondeclarative and not a question. Please place the study design ("A randomized controlled trial," "A retrospective study," "A modelling study," etc.) in the subtitle (ie, after a colon).

We thank the editor for the opportunity to make this change. We have since titled the manuscript; “Identifying care gaps along the HIV treatment failure cascade: a multi-state analysis of viral load monitoring, re-suppression, and regimen switches in Zambia”

* Please confirm that your abstract complies with our requirements, including format (three sections: Background, Methods and Findings, and Conclusions) and providing all the information relevant to this study type <https://journals.plos.org/plosmedicine/s/submission-guidelines#loc-abstract>

We confirm that the abstract complies with the journal requirements.

* Please ensure that the Introduction ends with a clear description of the study question or hypothesis.

We thank the editors for this guidance. We have included this in line 146 on page 6 of the tracked manuscript.

* Please ensure that all abbreviations are defined at first use throughout the text.

We have now made sure that all abbreviations are defined at first use except for HIV.

* Please confirm that all numbers presented in the abstract are present and identical to numbers presented in the main manuscript text.

We have made the necessary changes in line 311 to 312 to ensure that all numbers in the abstract are present and identical to the main manuscript.

* In the last sentence of the Abstract Methods and Findings section, please describe the main limitation(s) of the study's methodology.

We thank the editors for this recommendation. We have included this in line 55-57

“The study was limited by the inability to capture all relevant aspects of care delivery related to treatment failure such as outreach, confirmation of the delivery of EAC and provider rationale for delayed rechecking of VL tests.”

* In the author summary, please revise formatting and ensure you use bullet points. The author summary should be briefer with ~3 bullet points per subheading.

We thank the editors for the opportunity to revise the author summary from line 66 to make it brief with the use of bullet points.

* In the author summary, in the final bullet point of 'What Do These Findings Mean?', please include the main limitations of the study in non-technical language.

We have added this to line 103-105. It now reads as follows:

“This study was limited by the inability to capture all relevant aspects of care delivery related to treatment failure such as outreach, confirmation of the delivery of EAC, and provider rationale for delayed rechecking of VL test.”

* Please clarify whether consent was obtained or waived.

We obtained a waiver of consent to use Zambia’s electronic health records for measuring clinical care and outcomes.

* Please include descriptive legends for all figures (e.g. Figs. 2 and 5), and for supplementary figures.

We thank the editors for pointing this out. We have now included the legend for Fig 2, Fig 5 and the supplementary figures.

* Please ensure that the study is reported according to the STROBE guideline, and include the completed STROBE checklist as Supporting Information.  Please add the following statement, or similar, to the Methods: "This study is reported as per the Strengthening the Reporting of Observational Studies in Epidemiology (STROBE) guideline (S1 Checklist).”

This had been done

* Did your study have a prospective protocol or analysis plan? Please state this (either way) early in the Methods section.
a) If a prospective analysis plan (from your funding proposal, IRB or other ethics committee submission, study protocol, or other planning document written before analyzing the data) was used in designing the study, please include the relevant prospectively written document with your revised manuscript as a Supporting Information file to be published alongside your study, and cite it in the Methods section. A legend for this file should be included at the end of your manuscript.

We have moved the ethics statement to the top of the methods and included the following. the following:

“The research in this paper was not prespecified and consists of a secondary analysis of preexisting data.”

b) If no such document exists, please make sure that the Methods section transparently describes when analyses were planned, and when/why any data-driven changes to analyses took place.

c) In either case, changes in the analysis-- including those made in response to peer review comments-- should be identified as such in the Methods section of the paper, with rationale.
The STROBE guideline can be found here: <http://www.equator-network.org/reporting-guidelines/strobe/>
When completing the checklist, please use section and paragraph numbers, rather than page numbers.
* For all observational studies, in the manuscript text, please indicate: (1) the specific hypotheses you intended to test, (2) the analytical methods by which you planned to test them, (3) the analyses you actually performed, and (4) when reported analyses differ from those that were planned, transparent explanations for differences that affect the reliability of the study's results. If a reported analysis was performed based on an interesting but unanticipated pattern in the data, please be clear that the analysis was data-driven.
* Please remove the 'conclusions' subheading from the discussion. Please also remove any other subheadings from the discussion.

Done

* Please provide titles and legends for all figures and tables (including those in Supporting Information files). Please define all acronyms used in each figure or table in its corresponding legend.

Done

* Please consider avoiding the use of red and green in order to make your figure more accessible

Specific edits:

Abstract:
Line 33, delete sides. Revise sentence to say “…delays by both clients and health systems”.

We thank the editor for this suggestion. This change has been made.

The background of the Abstract should not identify the conclusions (if these are delays in HIV viral load….regimen switches”. Suggest to delete that clause (line 34).

This change has been made and now reads as follows:

“We used multistate analysis to identify drivers of implementation gaps in the treatment failure cascade in Zambia, including time to HIV viral load monitoring, resuppression, and regimen switches, in Zambia.”

Please avoid use of double brackets ([]) in the Abstract.

We have removed the double bracket in abstract.

The number of individuals excluded does not need to be in the manuscripts. Please simplify the information in lines 45-50. Please do not spell out confidence interval.

We have removed the following from the abstract (2,842 [2.0%] with elevated VL excluded for no documented drug regimen). It now reads as follows:

Among 179,855 individuals on ART (143,857 with a documented VL test), 7,916 (4.4%) had a documented elevated VL and drug regimen at the time of treatment failure and were included in the analysis. This cohort was 52.3% female, median age was 36.7 years (IQR 29.9-43.6), median time on ART was 3.3 years (IQR 1.7-6.6), and 54.6% were on TLD versus 45.4% on TLE.

Confidence intervals should be listed with a comma rather than a dash. Here and throughout.

We thank the editor for this guidance. We have since made this change throughout the whole manuscript.

Methods and Findings section of Abstract is very dense and would benefit from simplifying.

Thank you for the editor’s guidance. We have made changes to the Methods and Findings section of the abstract.

Lines 61-62: please avoid repeat use of “retention and adherence” and “fidelity to ….care”, which are also in the Background section.

We thank the editors for highlighting this redundancy and have revised the text accordingly.

Author summary: first section (Why was this study done?) is internally repetitive.

We thank the editors for highlighting this redundancy and have revised the text accordingly.

“Prompt, guideline-concordant responses to treatment failure are essential for improving viral suppression and treatment outcomes among people living with HIV (PLWH) with viremia.

However, in routine care settings in low- and middle-income countries, including Zambia, significant gaps persist along the treatment failure cascade such as delays in initiating enhanced adherence counselling (EAC), repeat VL testing, and timely regimen switching. These gaps may stem from both client-related challenges like treatment interruptions and health system factors, including provider adherence to guidelines.

This study aimed to characterize these implementation gaps, particularly in the context of Zambia’s transition to dolutegravir-based first-line therapy”

2nd section “What did the researchers do and find” : please simplify for the general reader and avoid including all details. Please also avoid repeating all of the results that are presented in the Abstract.

We thank the editors for this guidance. We have simplified this for the general reader as follows:

“We leveraged electronic health records between 2019 to 2021 from routine care from 24 public HIV clinics that serve 179,855 clients, including 7,916 individuals with documented treatment failure who were followed in this study. We applied multi-state analytic approach to comprehensively assess individuals’ care journey with respect to VL monitoring, treatment retention, regimen switching, and, ultimately, viral suppression.

We found that only 36.4% of patients had a VL rechecked within 6 months of failure, and only 50.1% had documented viral re-suppression at one year, driven in large part by delays in returning to clinic, treatment interruptions, and missed opportunities to recheck VLs after return.

Among those that did have a VL rechecked, rates of re-suppression were significantly higher for those on dolutegravir-based regimens (TLD) compared to those on efavirenz-based regimens (TLE), despite otherwise having similar care patterns.”

Line 92: delete robust (temper language)

We have now made a change to this section of the author summary. It reads as follows: “These findings reveal major implementation gaps in the treatment failure cascade…”

Include study limitations here and in the Methods and Findings section of the Abstract.

We have included the study limitations in the abstract:

“The study was limited by the inability to capture all relevant aspects of care delivery related to treatment failure such as outreach, confirmation of the delivery of EAC and provider rationale for delayed rechecking of VL tests.”

Please shorten lines 96-98. Please also note that the general, non-specialist reader would not understand the reference to dolutegravir-based regimens. Please clarify.

We thank the editors for this comment and have now revised this whole section. It reads as follows:

“Given the global shift toward more potent dolutegravir-based regimens with high barriers to resistance, these findings suggest modern approaches to treatment failure should prioritize strengthening health system processes for timely outreach and integrating person-centred strategies to improve engagement, rather than focus on clinical decision making (e.g., concerns for resistance and switches to second line) alone.”

Line 113: need a noun after biomedical.

We have added factors at the end of biomedical

Line 116: please remove formatting error

Thank you for pointing this out. The formatting error caused by the referencing software has been deleted.

Line 127—should this be failure rather than success?

We thank the editors for pointing this out. While we meant treatment success, we realise the ambiguity that this sentence poses. We have therefore revised to the following:

“On the client side, inadequate adherence and drug resistance remain concerns, while delays in returning to care for EAC and poor retention, which may preclude timely assessment of virologic status, are important drivers of treatment failure”

There is extensive repetition of concepts in the Introduction. Please reduce repetition in paragraphs 1 and 2 in particular.

We thank the editors for pointing out the repetition. We have now made changes to the introduction.

The Discussion exceeds 2000 words. Please reduce to 1200.

We have been able to reduce the discussion to 1500 words.

Comments from Reviewers:

Reviewer #1: The manuscript has been strengthened. There are still a few points that could potentially further improve this study as follows.

1)      COVID-19 disaggregated data. Thanks for looking carefully at this. I would suggest adding this to the supplemental data section and noting in the main text that your main findings were robust to this sensitivity analysis.

We thank the reviewers for this suggestion, and we have now included this in our methods section and added to the supplement.

2)      New recommendations (Reviewer 3). I agree with this reviewer that the contributions to the literature could be more explicit. What are things not recommended in Zambian or WHO guidelines that should be? (or put another way, what should health systems be doing differently)
 
We thank the reviewers for this recommendation. We have amended the following sentence in the last paragraph of our discussion that states more explicitly that formal guidance is needed around infrastructure for appropriate outreach and retention programs, rather than simply focusing on the clinical decision making.

“In the modern era of ART with TLD used as first line regimens, it is critical to reimagine approaches to treatment failure, and create formal and explicit guidance to prioritize person-centred strategies for timely engagement and support for client retention and adherence and likely less emphasis of resistance concerns and switches to second line.”

Reviewer #2: Thank you for addressing reviewers' comments throughout the manuscript. This version is much clearer than the prior submission. I have no major comments with this version, but the text is still hard to read in places due to the density of the writing and various grammar issues, missing words, inconsistencies, and some confusing sentences or phrases. My comments here relate to these and are relatively minor. Once addressed, I believe the manuscript can make an important contribution to the literature on patient experience with ART.

Specific Comments:

Abstract

1.      Page 2, line 48: "…and included in the analysis…" - it seems a word is missing. Consider adding "were" before "included" here.

We thank the reviewers for taking their time to go through our manuscript. We noted a similar request from the editor to remove those that were excluded from the manuscript and have since addressed this comment.

2.      Page 3, lines 61-62 "…strategies …. should be modernized…." It's not clear what 'modernized strategies" means and whether it refers to client or health systems or something else altogether. Can the authors please clarify this in the manuscript?

We thank the reviewers for raising this important issue. We have made changes to the abstract as follows:

“To optimize response and outcomes after treatment failure, strategies must prioritize and target both client and health systems behaviours to meet the care needs in the modern era of TLD.”

Introduction

3.      Page 5, line 113. The sentence ending with "… and biomedical" seems to have a word missing (e.g. "factors," "characteristics").

We have made a change to this sentence as it was highlighted by the editor too.

4.      The Introduction is generally very dense and a bit hard to understand in places. Simplifying in a few places would improve readability. Examples are 1) lines 125-26: "… delays in returning to care for EAC and poor retention that precludes assessment of virologic status…" (consider: "….delays in returning to care for EAC and poor retention that also delay viral load testing…") and 2) line 130: "By examining transitions through key steps in treatment failure cascade…." (consider "By examining transitions over the course of treatment….").

We thank the reviewers for raising similar points to the editor. We have rewritten the whole introduction of the manuscript.

5.      Page 5, line 164: "… were also informally used at the time as well…" - no need for both "also" and "as well."
We thank the reviewers for helping us make the manuscript clearer. We have made this change and it reads as follows:

“Throughout this period, guideline-recommended second-line regimens included protease inhibitor (PI)-based (i.e., ritonavir boosted lopinavir [LPVr] or atazanavir [ATVr]) and switching TDF to zidovudine (AZT) (e.g., AZT/XTC/LPVr as a second-line regimen), but integrase-based regimens (i.e., DTG) were informally used at the time”

Methods

6.      Page 9, line 227, please add "the" before "second" in the sentence "…. time zero was the date of second consecutive elevated VL…"

We have made this change in the manuscript, and it now reads as follows:

“For the second stage analysis, time zero was the date of second consecutive elevated VL”

7.      Page 11, line 256, can the authors please add a reference for the Stata software?

We have now included the STATA citation

Results

8.      Page 14, line 286: assuming the authors mean a viral load test, would recommend adding "test" after "viral load" in the sentence "… (including visits with and without a viral load)…."? There are many references to "VL" that clearly mean "VL test" or a re-test - consider clarifying by adding "test" or "check" (and re-test or re-check).

We thank the reviewers for their guidance. We have made this change throughout the results section and added test and where we use VL recheck, we imply a VL recheck after two elevated VLs and being due for switch.

We capture this in line 239:

“Among those on TLD, at 6 months, 24.0% (CI 20.5, 27.5%) were switched and 33.2% (CI 29.4, 37.3%) had another VL test rechecked for a third time prior to switch”

9.      Page 18, line 355: the sentence beginning with "14 (1.8%)…" Suggest revising slightly to "Only 14 (1.8%)…" to avoid starting the sentence with a number.

We agree with the input from the reviewers and have included “Only”.

Discussion

10.     Page 22, line 443: the sentence ending with " … are appropriately taken advantage of." Please consider revising to improve grammar: "…. serve to benefit patients optimally."

We thank the reviewers for their feedback. We have amended this sentence, and it now reads as follows:

“Addressing these issues requires system-level interventions to ensure that each health interaction is fully taken advantage of to support optimal care”

11.     Generally: two writing points: 1) the authors define the acronym VL for viral load but then use the two interchangeably. I suggest using VL consistently rather than using both; and 2) the authors use "VL" frequently to mean "VL test." It's clear what they mean but I still recommend using "test," "check," "re-test," or "re-check" as appropriate for clarity and consistency.

We have made this change across the whole manuscript and now us VL test.

Reviewer #3: My previous comments have been addressed, thank you.

We thank the reviewer for their previous comments that have helped shaped this paper.

Any attachments provided with reviews can be seen via the following link:
