# Supplementary material for: Identifying care gaps along the HIV treatment failure cascade: A multistate analysis of viral load monitoring, re-suppression, and regimen switches in Zambia
Source: PLoS Med. 2025 Sep 3;22(9):e1004720. doi: 10.1371/journal.pmed.1004720 (PMC12422583; doi:10.1371/journal.pmed.1004720)
Supplement: Peer review history

# Editor report: Decision Letter 0

**DOI:** 10.1371/journal.pmed.1004720.r001

**Authors:** Alison Farrell

25 Apr 2025

Dear Dr Sikombe,

Thank you for submitting your manuscript entitled "What drives implementation gaps along the treatment failure cascade? A multi-state analysis examining gaps in HIV viral load monitoring, resuppression, and regimen switches in Zambia" for consideration by PLOS Medicine.

Your manuscript has now been evaluated by the PLOS Medicine editorial staff as well as by an academic editor with relevant expertise and I am writing to let you know that we would like to send your submission out for external peer review.

However, before we can send your manuscript to reviewers, we need you to complete your submission by providing the metadata that is required for full assessment. To this end, please login to Editorial Manager where you will find the paper in the 'Submissions Needing Revisions' folder on your homepage. Please click 'Revise Submission' from the Action Links and complete all additional questions in the submission questionnaire.

Please re-submit your manuscript within two working days, i.e. by Apr 29 2025 11:59PM.

Login to Editorial Manager here: https://www.editorialmanager.com/pmedicine

Once your full submission is complete, your paper will undergo a series of checks in preparation for peer review. Once your manuscript has passed all checks it will be sent out for review.

Feel free to email us at plosmedicine@plos.org if you have any queries relating to your submission.

Kind regards,

Alison Farrell, Ph.D.

Senior Editor

PLOS Medicine

# Peer review: Decision Letter 1

**DOI:** 10.1371/journal.pmed.1004720.r002

**Authors:** Alison Farrell

12 Jun 2025

Dear Dr Sikombe,

Many thanks for submitting your manuscript "What drives implementation gaps along the treatment failure cascade? A multi-state analysis examining gaps in HIV viral load monitoring, resuppression, and regimen switches in Zambia" (PMEDICINE-D-25-01499R1) to PLOS Medicine. The paper has been reviewed by subject experts and a statistician; their comments are included below and can also be accessed here: [LINK]

As you will see, the reviewers find the work interesting and of potential importance and have made suggestions to clarify the background, analyses and results, and to extend the discussion. After discussing the paper with the editorial team and an academic editor with relevant expertise, I'm pleased to invite you to revise the paper in response to the reviewers' comments. We plan to send the revised paper to some or all of the original reviewers, and we cannot provide any guarantees at this stage regarding publication.

Please also revise the title to better reflect the analysis. Guidance on title format can be found here:https://journals.plos.org/plosmedicine/s/submission-guidelines. We ask that titles be specific, descriptive, concise, and comprehensible to readers outside the field, and we don't permit questions in the title. As your study did not evaluate what 'drives' the implementation gaps, we ask that the title more accurately reflect the analysis.

In addition to these revisions, you will need to complete some formatting changes, which you will receive in a follow up email. A member of our team will be in touch with a set of requests shortly.

When you upload your revision, please include a point-by-point response that addresses all of the reviewer and editorial points, indicating the changes made in the manuscript and either an excerpt of the revised text or the location (eg: page and line number) where each change can be found. Please also be sure to check the general editorial comments at the end of this letter and include these in your point-by-point response. When you resubmit your paper, please include a clean version of the paper as the main article file and a version with changes tracked as a marked-up manuscript. It may also be helpful to check the guidelines for revised papers at http://journals.plos.org/plosmedicine/s/revising-your-manuscript for any that apply to your paper.

We ask that you submit your revision by Jul 03 2025 11:59PM. However, if this deadline is not feasible, please contact me by email, and we can discuss a suitable alternative.

Don't hesitate to contact me directly with any questions (afarrell@plos.org).

Best regards,

Alison

Alison Farrell, Ph.D.

Senior Editor

PLOS Medicine

afarrell@plos.org

-----------------------------------------------------------

Comments from the academic editor:

Please expand the discussion a bit to stress the implications for other settings with a generalised epidemic. Please also add a discussion on possible causes of implementation gaps as may be suggested by your data.

-----------------------------------------------------------

Comments from the reviewers:

Reviewer #1: PLoS Medicine review

This manuscript examines implementation gaps in HIV service delivery in Zambia using a national electronic HIV record from 24 clinics. The strengths of this study include leveraging a national electronic database that goes down to local levels and detailed longitudinal data on individuals. This is an exciting manuscript that will be useful for those designing HIV services in LMICs.

MAJOR COMMENTS

1) COVID-19 impact. The study duration includes periods both prior to and during the COVID-19 pandemic. While the extent of Zambian lockdown was less than many other countries, this could also plausibly impact what happens among PLHIV who are not virally suppressed. Based on the data from Figure S1, it does seem like there were some major changes in the distribution of individuals with an elevated viral load around that time. Would it be possible to do a sensitivity analysis that disaggregates the data based on before and after Dec 2020 (the second wave when 501.V2 variant substantially spread through Zambia)? Are the main findings robust when data are disaggregated this way? Data from Table S5 suggest that this is an important factor that could be incorporated more into the analysis.

2) Health professional-level data. Within health systems, it would also seem that health professional level issues would be critical here. Does the electronic health system record include some indication of the health professional who assessed the patient? I assume that this granular information is not included in the data. If this is the case, it might be worth adding to the limitations.

MINOR COMMENTS

1) Abstract results section. Some of the estimates are have a range (eg, 11.4% (10.3 - 12.4)) without stating if the range represents a 95% CI or something else. Would be good to clarify these ranges.

2) Introduction, first sentence. Consider condensing this sentence or separating it into two sentences.

3) Missing VL group. The 20% of people who are missing a viral load is substantial and likely overlaps with treatment failure. Could these individuals have received a viral load at a clinic outside of the 24 included in this study? Is there another national database that could be used to track these people? My guess is "no", but interested to hear if there are other ways to capture this.

Reviewer #2: To authors:

I want to congratulate the authors on some fine work. This was a fascinating, relevant, and very well written manuscript. I really enjoyed reading it. I have just a few minor questions and suggestions - mainly things to clarify some sections that were a bit hard to follow in Methods and thoughts on relevant literature for the discussion. I hope they are helpful to the authors.

Specific Comments:

Abstract

1. A minor note that it seems a bit odd to mix up tenses (present tense in Background, but past tense in Methods). I'd suggest being consistent and using past tense throughout.

2. In Results section of the abstract, can the authors add what proportion of total ART patients were represented by the individuals with an elevated VL and at how long since ART initiation they were (median months, for instance)? This would be useful info for context.

3. In Results, the 72.2% who returned to clinic within 6 months of initial treatment failure. Is this when the treatment failure was documented, or when the individuals learned of their treatment failure?

Methods

4. Page 5: in the first paragraph of the Study population and setting section, could the authors add a brief description of the HIV care environment in Lusaka - how many individuals estimated to be HIV+, how many on ART, and the care cascade (proportion that know their HIV status; proportion on ART; proportion with suppressed VL)? The Zambia HIV epidemic is presumably a generalized epidemic but are there other trends underway that characterize the epidemic in Lusaka or Zambia more broadly (e.g. any particularly vulnerable populations)? This is important background context for the study.

5. Page 5, last line - what determines when the repeat VL should be between 3 and 6 months? That is, on what basis is the timing of the repeat VL determined?

6. On page 6-7, lines 134-137, this statement is hard to follow: "We accounted for transitions between the various care states that occur prior to having a subsequent VL measure repeated…. (e.g. ….. repeat VL suppressed versus unsuppressed." It's unclear how one can account for transitions prior to the next VL, where transitions include the repeated VL. Can you explain this?

7. Page 7, lines 139-144: this reads as if the 9 states are mutually exclusive throughout the period of observation (e.g. that if a client was in category 2 or 3, they would have no treatment interruption). But figure 1 - both text and figure itself (which is quite a work of art) and the later text on page 8 clarifies that categorization could happen twice, with a total of 19 possible categories for each client. To avoid confusion when reading the first part of this (on page 7, lines 139-144—which I re-read at least 3 times to try to understand how a total of 9 categories could make sense), I think it would help the reader if the text stated this more clearly, e.g. "In the first stage of categorization, clients were categorized into one of 9 …." Then readers know that more is coming in the next stage of categorization (with many more than 9 total categories used).

8. Page 7, line 147 - what is the Markov assumption being referred to here? That clients could move between nonabsorbable states an unlimited number of times? That sentence is a bit confusing.

Results

9. Page 13, line 243: it is not clear what this means "… and median time from returning to having VL repeated was 63 days." Does it mean median time between returning to clinic after the initial elevated VL and the actual repeated VL test was 63 days? E.g., this is the lag between first return to clinic and having VL test repeated?

Discussion

10. Page 19, paragraph beginning in line 350: the association found between longer appointment duration after initial treatment failure and longer time to return to clinic is notable. The authors stress that care delivery needs to be responsive and flexible in contacting patients with elevated VL and providing them with support to improve adherence and increase likelihood of viral suppression. The authors may be interested in a clinical trial underway in South Africa that is addressing just this issue by monitoring individuals who have just initiated ART (using 3 different methods, including contact immediately following an elevated VL) and giving them support (see Jennings et al, "Protocol for an evaluation of adherence monitoring and support interventions among people initiating antiretroviral therapy in Cape Town, South Africa - A multiphase optimization strategy (MOST) approach using a fractional factorial design." Trials. 2023.)

11. The authors do a nice job with their second discussion point highlighting the common experience of treatment gaps and the many barriers to retention in care, as well as the role providers may play in discouraging individuals from returning to care to recommence treatment. For related evidence gathered from community clinics in nearby Cape Town, see Killian C, et al, "Negative clinic experiences as a barrier to care for people with HIV and their impact on patient preferences for intervention support: A qualitative study in Cape Town, South Africa." AIDS Care. 2024.

Reviewer #3: Overall

Thank you for the opportunity to review this well written manuscript of outcomes after viraemia in routine care in Zambia. This is a comprehensive and generally well conducted set of analyses. However, the findings are not particularly novel, and so I question whether this would meet the PLoS Medicine threshold of having "potential to directly and substantially inform clinical practice or health policy" - there are no new recommendations arising that are not already known/in place as far as I could see.

MINOR

ABSTRACT

INTRODUCTION

Line 65-66: Resuppression rates on dolutegravir likely differ from EFV which was likely the dominant anchor drug in the studies summarised in the two cited papers

Line 92: Wouldn't the transition to DTG also effect resuppression due to lack of drug resistance i.e. virological response to improved adherence?

Line 98-99: But no assessment of whether EAC was done or not?

METHODS

Line 100: What is the threshold for elevated VL? 50 cps/ml, 200, 1000?

Line 128: Are there any published studies or other evidence which have evaluated data capture completeness in this dataset, to help understand how much of treatment interruption or lack of repeat VLS could be due to data capture issues?

Line 137: This sentence focusses on states before repeat VL is done, but then includes repeat VL suppressed/unsuppressed

Line 141: I would question the assumption that people remain viraemic just because they haven't had a visit. In our experience in South Africa, many people with viraemia have disengaged (though maybe this isn't apparent to the HCW) and the VL is taken on the day they re-engage with care, and so they likely have restarted ART and so may be suppressing

Line 199: "Independently associated" - its stated that DAGs were used to determine what risk factors to include in multivariable Cox models, but then it appears in table S5 that there is just one large multivariable model with all the variables thrown in, and also 3 different outcomes with the same covariates in the model. Ideally (to avoid "Table 2 Fallacy") a model should be built to assess the relationship between a specific exposure and specific outcome, with potential confounders selected on whether they might confound that specific relationship. So maybe rephrasing the aim of this analysis is needed.

Line 199: How was correlation within facilities accounted for in the Cox models?

RESULTS

Lines 239-250: Would be nice to see n/N or at least the denominator for the percentages presented. E.g. are the 50% with resuppression at 12 months 50% of those who had VL done, or 50% of cohort?

Line 278: "Among those on TLD at 6 months, …" its not clear to me why this is at 6 months. Should it not be among those on TLD at second repeat VL (ie at entry into this 'eligible for switch' cohort". - On re-reading I think this should read "Among those on TLD, at 6 months…."

Line 278: Again, would be nice to know the n/N here to know how many people in the TLD group and how many on TLD were switched to second-line - seems from the next paragraph that it was very few.

Line 304: I wouldn't classify switching from TLD to AZT/XTC/DTG as a change to second-line ART, there is no change in anchor drug. Could this have been a switch due to renal toxicity?

Line 307: Are the results post switch presented for the whole cohort? It would be nice to see them by TLD versus TLE too.

Line 317: "having a longer appointment duration after initial treatment failure was highly associated with longer time to return" - This seems obvious - longer intervals means people will come back later? Or am I misunderstanding?

DISCUSSION

In general, the discussion is quite long and in places makes quite broad recommendations that go slightly beyond the findings of the presented analyses.

Line 350: "lack of prompt initiation of EAC" - but this study did not look at when EAC was initiated…

Line 353: The emphasis has been placed on strong relationship between appointment duration and time to repeat VL, whereas in the results emphasis is on relationship with outcome of time to repeat clinic visit.

Line 369: "In our study, approximately 30% of individuals had been 30 days late for an appointment at least once after treatment failure, and 59.2% of those who did not have a VL rechecked by 1 year (and had not died or transferred) were in a treatment interruption" Sorry if I missed it but don't recall these being presented in the results. And wasn't the treatment interruption definition >60 days?

Line 394: "Our study results also provide important new insights into outcomes after treatment failure during routine care as programs shift from TLE to TLD.." There are several published papers looking at this

https://academic.oup.com/ofid/article/10/12/ofad583/7424682

https://academic.oup.com/ofid/article/11/2/ofae013/7512841

https://journals.lww.com/jaids/fulltext/2024/04150/viremia_and_hiv_drug_resistance_among_people.14.aspx

https://journals.lww.com/aidsonline/fulltext/2024/09010/longitudinal_viral_load_outcomes_of_adults_with.12.aspx

Line 399: "Assuming similar levels of adherence" - could also be due to less side effects with DTG? Especially low level side effects that could tip people over the edge of not taking treatment regularly

Line 409: What were the Zambian guidelines regarding components of the second line regimen after failure on TLD?

Line 439: "driven by delays in returning for adherence counselling" this seems to place the blame on the client as they didn't return, rather than the healthcare system which may not have recalled them promptly

TABLES AND FIGURES

Table 1:

The rows for ART regimen are not necessary as represented in columns?

Would be nice to see time on current regimen at first elevated VL

Fig 3:

- Same comment as above re problems with assuming those that haven't returned are still viraemic

Fig 4: It would be nice to see the N numbers for each figure (esp TLD and TLE)

Jienchi Dorward

Any attachments provided with reviews can be seen via the following link: [LINK]

--------------------------------------------------------- ---

General editorial requests:

(Note: not all will apply to your paper, but please check each item carefully)

* We ask every co-author listed on the manuscript to fill in a contributing author statement, making sure to declare all competing interests. If any of the co-authors have not filled in the statement, we will remind them to do so when the paper is revised. If all statements are not completed in a timely fashion this could hold up the re-review process. If new competing interests are declared later in the revision process, this may also hold up the submission. Should there be a problem getting one of your co-authors to fill in a statement we will be in contact. Please do not add or remove authors without first discussing this with the handling editor. You can see our competing interests policy here: http://journals.plos.org/plosmedicine/s/competing-interests.

* Please upload any figures associated with your paper as individual TIF or EPS files with 300dpi resolution at resubmission; please read our figure guidelines for more information on our requirements: http://journals.plos.org/plosmedicine/s/figures. While revising your submission, please upload your figure files to the PACE digital diagnostic tool, https://pacev2.apexcovantage.com/. PACE helps ensure that figures meet PLOS requirements. To use PACE, you must first register as a user. Then, login and navigate to the UPLOAD tab, where you will find detailed instructions on how to use the tool. If you encounter any issues or have any questions when using PACE, please email us at PLOSMedicine@plos.org.

* Please ensure that the paper adheres to the PLOS Data Availability Policy (see http://journals.plos.org/plosmedicine/s/data-availability), which requires that all data underlying the study's findings be provided in a repository or as Supporting Information. Please ensure that the Data Availability Statement details any restriction to data and the process for data access. For data residing with a third party, authors are required to provide instructions with contact information (web or email address) for obtaining the data. Please note that a study author cannot be the contact person for the data. PLOS journals do not allow statements supported by "data not shown" or "unpublished results." For such statements, authors must provide supporting data or cite public sources that include it.

* We expect all researchers with submissions to PLOS in which author-generated code underpins the findings in the manuscript to make all author-generated code available without restrictions upon publication of the work. In cases where code is central to the manuscript, we may require the code to be made available as a condition of publication. Authors are responsible for ensuring that the code is reusable and well documented. Please make any custom code available, either as part of your data deposition or as a supplementary file. Please add a sentence to your data availability statement regarding any code used in the study, e.g. "The code used in the analysis is available from Github [URL] and archived in Zenodo [DOI link]" Please review our guidelines at https://journals.plos.org/plosmedicine/s/materials-software-and-code-sharing and ensure that your code is shared in a way that follows best practice and facilitates reproducibility and reuse. Because Github depositions can be readily changed or deleted, we encourage you to make a permanent DOI'd copy (e.g. in Zenodo) and provide the URL.

* Please ensure that the study is reported according to the [XXXX] guideline and include the completed [XXXX] checklist as Supporting Information. When completing the checklist, please use section and paragraph numbers, rather than page numbers. Please add the following statement, or similar, to the Methods: "This study is reported as per [XXXX] guideline (S1 Checklist)."

FORMATTING - GENERAL

* Abstract: Please structure your abstract using the PLOS Medicine headings (Background, Methods and Findings, Conclusions). Please combine the Methods and Findings sections into one section.

* At this stage, we ask that you include a short, non-technical Author Summary of your research to make findings accessible to a wide audience that includes both scientists and non-scientists. The Author Summary should immediately follow the Abstract in your revised manuscript. This text is subject to editorial change and should be distinct from the scientific abstract. Ideally each sub-heading should contain 2-3 single sentence, concise bullet points containing the most salient points from your study. In the final bullet point of 'What Do These Findings Mean?', please include the main limitations of the study in non-technical language. Please see our author guidelines for more information: https://journals.plos.org/plosmedicine/s/revising-your-manuscript#loc-author-summary.

* Please express the main results with 95% CIs as well as p values. When reporting p values please report as p<0.001 and where higher as the exact p value p=0.002, for example. Throughout, suggest reporting statistical information as follows to improve clarity for the reader "22% (95% CI [13%,28%]; p</=)". Please be sure to define all numerical values at first use.

* Please include page numbers and line numbers in the manuscript file. Use continuous line numbers (do not restart the numbering on each page).

* Please cite the reference numbers in square brackets. Citations should precede punctuation.

FIGURES AND TABLES

* Please provide titles and legends for all figures and tables (including those in Supporting Information files).

* Please define all abbreviations used in each figure/table (including those in Supporting Information files).

* Please consider avoiding the use of red and green in order to make your figure more accessible to those with color blindness.

SUPPLEMENTARY MATERIAL

* Please note that supplementary material will be posted as supplied by the authors. Therefore, please amend it according to the relevant comments outlined here.

* Please cite your Supporting Information as outlined here: https://journals.plos.org/plosmedicine/s/supporting-information

REFERENCES

* PLOS uses the numbered citation (citation-sequence) method and first six authors, et al.

* Please ensure that journal name abbreviations match those found in the National Center for Biotechnology Information (NCBI) databases (http://www.ncbi.nlm.nih.gov/nlmcatalog/journals), and are appropriately formatted and capitalised.

* Where website addresses are cited, please include the complete URL and specify the date of access (e.g. [accessed: 12/06/2023]).

* Please also see https://journals.plos.org/plosmedicine/s/submission-guidelines#loc-references for further details on reference formatting.

[STUDY TYPE-SPECIFIC REQUESTS - DELETE SECTIONS AS NECESSARY]

RCTs [REFER TO RCT CHECKLIST AND MEETING NOTES FOR DETAILS TO ADD]

* PLOS Medicine requires that all trials be prospectively registered in one of registries recognized by WHO. Please ensure that study registration details are included in the Methods section.

* Please structure the Methods section using the following sub-headings: Study design and participants, Randomization and masking, Procedures, Outcomes, Statistical analysis.

* The following outcomes measures [ADD DETAILS AS NEEDED OR DELETE BULLET POINT] appear to differ between the submitted manuscript and the protocol [and/or trial registry]. Please clarify and explain all discrepancies between the paper and protocol. If the outcomes were not prespecified in the protocol, please define them in the Methods (Outcomes section) as post hoc and explain why they were added. Post-hoc comparisons should be presented as hypothesis generating rather than conclusive.

* Please ensure that all prespecified outcomes (primary, secondary, and exploratory) are listed in the Methods/Outcomes section and indicate whether there are outcomes that are not presented in the current report.

* Please specify the dates (Month Day, Year) during which study enrollment and follow up occurred.

* Please include absolute numbers wherever you report percentages; eg, n/N (%)

* Please present the safety data for the study including numbers of specific events and whether or not adverse events are thought to be related to treatment. AEs should be reported in the abstract, per CONSORT and CONSORT-Harms.

* Please complete the CONSORT checklist (https://www.equator-network.org/reporting-guidelines/consort/) and ensure that all components of CONSORT are present in the manuscript, including how randomization was performed, allocation concealment, blinding of intervention, definition of lost to follow-up, power statement. When completing the checklist, please use section and paragraph numbers, rather than page numbers.

* Please report your abstract according to CONSORT for abstracts, following the PLOS Medicine abstract structure (Background, Methods and Findings, Conclusions) https://www.equator-network.org/reporting-guidelines/consort-abstracts/

* If your trial had to undergo important modifications in response to extenuating circumstances, please complete the CONSERVE-CONSORT checklist and provide in your Supporting Information; (https://www.equator-network.org/reporting-guidelines/guidelines-for-reporting-trial-protocols-and-completed-trials-modified-due-to-the-covid-19-pandemic-and-other-extenuating-circumstances-the-conserve-2021-statement/). When completing the checklist, please use section and paragraph numbers, rather than page numbers.

* In keeping with our commitment to Open Science, please include the study protocol document and analysis plan (including any amendments) as Supporting Information to be published with the manuscript if accepted.

* Please note that PLOS Medicine requires prospective, public registration of a data sharing plan (as part of mandatory clinical trials registration) for all clinical trials that began enrollment on or after January 1, 2019, in accordance with ICMJE requirements.

OBSERVATIONAL STUDIES

* Abstract: Please include the study design, population and setting, number of participants, years during which the study took place (enrollment and follow up), length of follow up, and main outcome measures.

* Please ensure that the study is reported according to the STROBE (or appropriate STOBE extension) guideline (available from: https://www.equator-network.org/reporting-guidelines/strobe) and include the completed STROBE (or STROBE extension) checklist as Supporting Information. Please add the following statement, or similar, to the Methods: "This study is reported as per the Strengthening the Reporting of Observational Studies in Epidemiology (STROBE) guideline (S1 Checklist)." When completing the checklist, please use section and paragraph numbers, rather than page numbers.

* [FOR POPULATION HEALTH/REGISTRY STUDIES] Please ensure that the study is reported according to the RECORD guideline (available from https://www.record-statement.org) and include the completed checklist as Supporting Information. Please add the following statement, or similar, to the Methods: "This study is reported as per the Reporting of Studies Conducted using Observational Routinely-Collected Data (RECORD) guideline (S1 Checklist)." When completing the checklist, please use section and paragraph numbers, rather than page numbers.

* [FOR POPULATION HEALTH ESTIMATES] Please ensure that the study is reported according to the GATHER statement (available from https://www.equator-network.org/reporting-guidelines/gather-statement) and include the completed checklist as Supporting Information. Please add the following statement, or similar, to the Methods: "This study is reported as per the Guidelines for Accurate and Transparent Health Estimates Reporting (GATHER) statement (S1 Checklist)." When completing the checklist, please use section and paragraph numbers, rather than page numbers.

* [FOR MEDIATION ANALYSES] We recommend that the study is reported according to the AGReMA statement (https://agrema-statement.org/#:~:text=AGReMA%20is%20an%20evidence%2D%20and,randomised%20trials%20and%20observational%20studies) and include the completed checklist as Supporting Information. Please add the following statement, or similar, to the Methods: "This study is reported as per the Guideline for Reporting Mediation Analyses (AGReMA) statement (S1 Checklist)." When completing the checklist, please use section and paragraph numbers, rather than page numbers.

* For all observational studies, in the manuscript text, please indicate: (1) the specific hypotheses you intended to test, (2) the analytical methods by which you planned to test them, (3) the analyses you actually performed, and (4) when reported analyses differ from those that were planned, transparent explanations for differences that affect the reliability of the study's results. If a reported analysis was performed based on an interesting but unanticipated pattern in the data, please be clear that the analysis was data driven.

* Please state in the Methods section whether the study had a prospective protocol or analysis plan. If a prospective analysis plan (from your funding proposal, IRB or other ethics committee submission, study protocol, or other planning document written before analyzing the data) was used in designing the study, please include the relevant document(s) with your revised manuscript as a Supporting Information file to be published alongside your study and cite it in the Methods section. A legend for this file should be included at the end of your manuscript. If no such document exists, please make sure that the Methods section transparently describes when analyses were planned, and when/why any data-driven changes to analyses took place. Changes in the analysis, including those made in response to peer review comments, should be identified as such in the Methods section of the paper, with rationale.

MODELLING STUDIES

The following list is derived from Geoffrey P Garnett, Simon Cousens, Timothy B Hallett, Richard Steketee, Neff Walker. Mathematical models in the evaluation of health programmes. (2011) Lancet DOI:10.1016/S0140-6736(10)61505-X:

* If pertinent, please provide a diagram that shows the model structure, including how the natural history of the disease is represented, the process and determinants of disease acquisition, and how the putative intervention could affect the system.

* Please provide a complete list of model parameters, including clear and precise descriptions of the meaning of each parameter, together with the values or ranges for each, with justification or the primary source cited and important caveats about the use of these values noted.

* Please provide a clear statement about how the model was fitted to the data, including goodness-of-fit measure, the numerical algorithm used, which parameter varied, constraints imposed on parameter values, and starting conditions.

* For uncertainty analyses, please state the sources of uncertainties quantified and not quantified [can include parameter, data, and model structure].

* Please provide sensitivity analyses to identify which parameter values are most important in the model. Uncertainty estimates seek to derive a range of credible results on the basis of an exploration of the range of reasonable parameter values. The choice of method should be presented and justified.

* Please discuss the scientific rationale for the choice of model structure and identify points where this choice could influence conclusions drawn. Please also describe the strength of the scientific basis underlying the key model assumptions.

* For studies that develop a prediction model or evaluate its performance, please ensure that the study is reported according to the TRIPOD statement (https://www.equator-network.org/reporting-guidelines/tripod-statement) and include the completed checklist as Supporting Information. Please add the following statement, or similar, to the Methods: "This study is reported as per the Transparent Reporting of a Multivariable Prediction Model for Individual Prognosis Or Diagnosis (TRIPOD) statement (S1 Checklist)." For studies using machine learning, please use the TRIPOD-AI checklist. When completing the checklist, please use section and paragraph numbers, rather than page numbers.

DIAGNOSTIC STUDIES

* Please ensure that the study is reported according to the STARD guideline (https://www.equator-network.org/reporting-guidelines/stard/) and include the completed STARD checklist as Supporting Information. Please add the following statement, or similar, to the Methods: "This study is reported as per the Standards for Reporting of Diagnostic Accuracy (STARD) guideline (S1 Checklist)." When completing the checklist, please use section and paragraph numbers, rather than page numbers.

* Please structure your Abstract according to STARD for Abstracts (https://www.equator-network.org/reporting-guidelines/stard-abstracts/).

* Please structure the Methods section using the following sub-headings: Study design, Participants, Test methods, Analysis.

* Please include a diagram to describe the flow of participants through the study (typically figure 1).

MENDELIAN RANDOMIZATION STUDIES

* Please ensure that the study is reported according to the STROBE-MR guideline (https://www.equator-network.org/reporting-guidelines/strobe/) and include the completed STROBE-MR checklist as Supporting Information. Please add the following statement, or similar, to the Methods: "This study is reported as per the Strengthening the Reporting of Observational Studies in Epidemiology (STROBE) guideline, specific for mendelian randomization (S1 Checklist)." When completing the checklist, please use section and paragraph numbers, rather than page numbers.

* In the Introduction, please describe the exposure and the evidence for a potential causal relationship between exposure and outcome.

* In the Methods, please explicitly state the 3 core instrumental variable assumptions for the main analysis (relevance, independence, and exclusion restriction), as well assumptions for any additional or sensitivity analysis.

* In the Methods, please describe the MR estimator (e.g., 2-stage least squares, Wald ratio) and related statistics. Detail the included covariates and, in case of 2-sample MR, whether the same covariate set was used for adjustment in the 2 samples.

* If you are presenting an instrumental variable estimate, please compare this to the conventional observational estimate.

* Report the associations between genetic variant and exposure and between genetic variant and outcome, preferably on an interpretable scale.

* Report MR estimates of the relationship between exposure and outcome and the measures of uncertainty from the MR analysis, on an interpretable scale, such as odds ratio or relative risk per SD difference.

* If relevant, please consider translating estimates of relative risk into absolute risk for a meaningful time period.

* Please consider including plots to visualize results (e.g., forest plot, scatterplot of associations between genetic variants and outcome vs between genetic variants and exposure).

SURVEY-BASED STUDIES

* Please ensure that the study is reported according to the CROSS guideline (https://www.equator-network.org/reporting-guidelines/a-consensus-based-checklist-for-reporting-of-survey-studies-cross/) and include the completed CROSS checklist as Supporting Information. Please add the following statement, or similar, to the Methods: "This study is reported as per A Consensus-Based Checklist for Reporting of Survey Studies (CROSS) guideline (S1 Checklist)." When completing the checklist, please use section and paragraph numbers, rather than page numbers.

* Please report your survey response rates according to AAPOR recommendations (https://aapor.org/standards-and-ethics/best-practices/)

* Please define how the population surveyed was sampled.

* Please compare characteristics of respondents and nonrespondents if possible.

* If sequential waves of the survey were sent, please specify whether the characteristics of respondents changed over time or remained constant.

* Please include the survey response rate in the Abstract.

* Please include a copy of the survey in the supplementary files.

SYSTEMATIC REVIEWS & META-ANALYSES

* Please report your SR/MA according to the PRISMA guidelines provided at the EQUATOR site. http://www.equator-network.org/reporting-guidelines/prisma/. Please provide the completed PRISMA checklist as Supporting Information. When completing the checklist, please use section and paragraph numbers, rather than page numbers. Please add the following statement, or similar, to the Methods: "This study is reported as per the Preferred Reporting Items for Systematic Reviews and Meta-Analyses (PRISMA) guideline (S1 Checklist)."

* Abstract: Please report your abstract according to PRISMA for abstracts (https://doi.org/10.1371/journal.pmed.1001419) following the PLOS Medicine abstract structure (Background, Methods and Findings, Conclusions). Please ensure you provide dates of search, data sources, number of studies included, types of study designs included, eligibility criteria, and synthesis/appraisal methods.

* Please note that we expect searches to be updated to within 6 months of the time of submission.

QUALITATIVE STUDIES

* Please report your qualitative study according to the appropriate study design provided at (http://www.equator-network.org/?post_type=eq_guidelines&eq_guidelines_study_design=qualitative-research&eq_guidelines_clinical_specialty=0&eq_guidelines_report_section=0&s=) and provide the relevant completed checklist as a supplemental file. In the checklist, please include sufficient text excerpted from the manuscript to explain how you accomplished all applicable items. When completing checklists, please use section and paragraph numbers, rather than page numbers.

* We recommend that authors use the COREQ checklist, or other relevant checklists listed by the Equator Network, such as the SRQR, to ensure complete reporting (see: http://www.equator-network.org/?post_type=eq_guidelines&eq_guidelines_study_design=qualitative-research&eq_guidelines_clinical_specialty=0&eq_guidelines_report_section=0&s=). Please add the following statement, or similar, to the Methods: "This study is reported as per the Consolidated criteria for reporting qualitative research (COREQ): a 32-item checklist for interviews and focus groups (S1 Checklist)."

* In general, we expect qualitative studies to include the following: 1) defined objectives or research questions; 2) description of the sampling strategy, including rationale for the recruitment method, participant inclusion/exclusion criteria and the number of participants recruited; 3) detailed reporting of the data collection procedures; 4) data analysis procedures described in sufficient detail to enable replication; 5) a discussion of potential sources of bias; and 6) a discussion of limitations.

HEALTH ECONOMICS / COST-EFFECTIVENESS STUDIES

* Please ensure that the study is reported according to the CHEERS guideline (available from: https://www.equator-network.org/reporting-guidelines/cheers) and include the completed checklist as Supporting Information. Please add the following statement, or similar, to the Methods: "This study is reported as per the Strengthening the Consolidated Health Economic Evaluation Reporting Standards 2022 (CHEERS 2022) Statement (S1 Checklist)." When completing the checklist, please use section and paragraph numbers, rather than page numbers.

# Author comment: Author response to Decision Letter 2

**DOI:** 10.1371/journal.pmed.1004720.r003

3 Jul 2025

Attachment Submitted filename: Response to reviewers_Gaps in VL monitoring_02_07_2025.docx

# Peer review: Decision Letter 2

**DOI:** 10.1371/journal.pmed.1004720.r004

**Authors:** Alison Farrell

31 Jul 2025

Dear Dr. Sikombe,

Thank you very much for re-submitting your manuscript "Defining care gaps along the HIV treatment failure cascade: a multi-state analysis examining gaps in viral load monitoring, resuppression, and regimen switches in Zambia" (PMEDICINE-D-25-01499R2) for review by PLOS Medicine.

I have discussed the paper with my colleagues and the academic editor and it was also seen again by three reviewers. I am pleased to say that provided the remaining editorial and production issues are dealt with we are planning to accept the paper for publication in the journal. Please note that reviewers 1 request some additions to the manuscript and reviewer 2 requests additional edits for clarity and readability. Please note we request that you revise the title

The remaining issues that need to be addressed are listed at the end of this email. Any accompanying reviewer attachments can be seen via the link below. Please take these into account before resubmitting your manuscript:

***Please note while forming your response, if your article is accepted, you may have the opportunity to make the peer review history publicly available. The record will include editor decision letters (with reviews) and your responses to reviewer comments. If eligible, we will contact you to opt in or out.***

In revising the manuscript for further consideration here, please ensure you address the specific points made by each reviewer and the editors. In your rebuttal letter you should indicate your response to the reviewers' and editors' comments and the changes you have made in the manuscript. Please submit a clean version of the paper as the main article file. A version with changes marked must also be uploaded as a marked up manuscript file.

Please also check the guidelines for revised papers at http://journals.plos.org/plosmedicine/s/revising-your-manuscript for any that apply to your paper. If you haven't already, we ask that you provide a short, non-technical Author Summary of your research to make findings accessible to a wide audience that includes both scientists and non-scientists. The Author Summary should immediately follow the Abstract in your revised manuscript. This text is subject to editorial change and should be distinct from the scientific abstract.

In addition to these revisions, you may need to complete some formatting changes, which you will receive in a follow up email. A member of our team will be in touch with a set of requests shortly. If you do not receive a separate email within a few days, please assume that checks have been completed, and no additional changes are required.

We expect to receive your revised manuscript within 1 week. Please email us (plosmedicine@plos.org) if you have any questions or concerns.

We ask every co-author listed on the manuscript to fill in a contributing author statement. If any of the co-authors have not filled in the statement, we will remind them to do so when the paper is revised. If all statements are not completed in a timely fashion this could hold up the re-review process. Should there be a problem getting one of your co-authors to fill in a statement we will be in contact. YOU MUST NOT ADD OR REMOVE AUTHORS UNLESS YOU HAVE ALERTED THE EDITOR HANDLING THE MANUSCRIPT TO THE CHANGE AND THEY SPECIFICALLY HAVE AGREED TO IT.

Please ensure that the paper adheres to the PLOS Data Availability Policy (see http://journals.plos.org/plosmedicine/s/data-availability), which requires that all data underlying the study's findings be provided in a repository or as Supporting Information. For data residing with a third party, authors are required to provide instructions with contact information for obtaining the data. PLOS journals do not allow statements supported by "data not shown" or "unpublished results." For such statements, authors must provide supporting data or cite public sources that include it.

To enhance the reproducibility of your results, we recommend that you deposit your laboratory protocols in protocols.io, where a protocol can be assigned its own identifier (DOI) such that it can be cited independently in the future. Additionally, PLOS ONE offers an option to publish peer-reviewed clinical study protocols. Read more information on sharing protocols at https://plos.org/protocols?utm_medium=editorial-email&utm_source=authorletters&utm_campaign=protocols

Please review your reference list to ensure that it is complete and correct. If you have cited papers that have been retracted, please include the rationale for doing so in the manuscript text, or remove these references and replace them with relevant current references. Any changes to the reference list should be mentioned in the rebuttal letter that accompanies your revised manuscript.

Please note, when your manuscript is accepted, an uncorrected proof of your manuscript will be published online ahead of the final version, unless you've already opted out via the online submission form. If, for any reason, you do not want an earlier version of your manuscript published online or are unsure if you have already indicated as such, please let the journal staff know immediately at plosmedicine@plos.org.

If you have any questions in the meantime, please contact me or the journal staff on plosmedicine@plos.org.  

We look forward to receiving the revised manuscript by Aug 07 2025 11:59PM.   

Sincerely,

Alison Farrell, Ph.D.

Senior Editor 

PLOS Medicine

plosmedicine.org

------------------------------------------------------------

Requests from Editors:

Please revise the title to comply with PLOS Medicine style: e.g. Identifying gaps in HIV viral load monitoring, resusppression and regimen switches in Zambia: a multi-state analysis OR Identifying drivers of HIV treatment failure in Zambia: a multi-state analysis.

Your title must be nondeclarative and not a question. Please place the study design ("A randomized controlled trial," "A retrospective study," "A modelling study," etc.) in the subtitle (ie, after a colon).

* Please confirm that your abstract complies with our requirements, including format (three sections: Background, Methods and Findings, and Conclusions) and providing all the information relevant to this study type https://journals.plos.org/plosmedicine/s/submission-guidelines#loc-abstract

* Please ensure that the Introduction ends with a clear description of the study question or hypothesis.

* Please ensure that all abbreviations are defined at first use throughout the text.

* Please confirm that all numbers presented in the abstract are present and identical to numbers presented in the main manuscript text.

* In the last sentence of the Abstract Methods and Findings section, please describe the main limitation(s) of the study's methodology.

* In the author summary, please revise formatting and ensure you use bullet points. The author summary should be briefer with ~3 bullet points per subheading.

* In the author summary, in the final bullet point of 'What Do These Findings Mean?', please include the main limitations of the study in non-technical language.

* Please clarify whether consent was obtained or waived.

* Please include descriptive legends for all figures (e.g. Figs. 2 and 5), and for supplementary figures.

* Please ensure that the study is reported according to the STROBE guideline, and include the completed STROBE checklist as Supporting Information. Please add the following statement, or similar, to the Methods: "This study is reported as per the Strengthening the Reporting of Observational Studies in Epidemiology (STROBE) guideline (S1 Checklist).”

* Did your study have a prospective protocol or analysis plan? Please state this (either way) early in the Methods section.

a) If a prospective analysis plan (from your funding proposal, IRB or other ethics committee submission, study protocol, or other planning document written before analyzing the data) was used in designing the study, please include the relevant prospectively written document with your revised manuscript as a Supporting Information file to be published alongside your study, and cite it in the Methods section. A legend for this file should be included at the end of your manuscript.

b) If no such document exists, please make sure that the Methods section transparently describes when analyses were planned, and when/why any data-driven changes to analyses took place.

c) In either case, changes in the analysis-- including those made in response to peer review comments-- should be identified as such in the Methods section of the paper, with rationale.

The STROBE guideline can be found here: http://www.equator-network.org/reporting-guidelines/strobe/

When completing the checklist, please use section and paragraph numbers, rather than page numbers.

* For all observational studies, in the manuscript text, please indicate: (1) the specific hypotheses you intended to test, (2) the analytical methods by which you planned to test them, (3) the analyses you actually performed, and (4) when reported analyses differ from those that were planned, transparent explanations for differences that affect the reliability of the study's results. If a reported analysis was performed based on an interesting but unanticipated pattern in the data, please be clear that the analysis was data-driven.

* Please remove the 'conclusions' subheading from the discussion. Please also remove any other subheadings from the discussion.

* Please provide titles and legends for all figures and tables (including those in Supporting Information files). Please define all acronyms used in each figure or table in its corresponding legend.

* Please consider avoiding the use of red and green in order to make your figure more accessible

Specific edits:

Abstract:

Line 33, delete sides. Revise sentence to say “…delays by both clients and health systems”.

The background of the Abstract should not identify the conclusions (if these are delays in HIV viral load….regimen switches”. Suggest to delete that clause (line 34).

Please avoid use of double brackets ([]) in the Abstract.

The number of individuals excluded does not need to be in the manuscripts. Please simplify the information in lines 45-50. Please do not spell out confidence interval.

Confidence intervals should be listed with a comma rather than a dash. Here and throughout.

Methods and Findings section of Abstract is very dense and would benefit from simplifying.

Lines 61-62: please avoid repeat use of “retention and adherence” and “fidelity to ….care”, which are also in the Background section.

Author summary: first section (Why was this study done?) is internally repetitive.

2nd section “What did the researchers do and find” : please simplify for the general reader and avoid including all details.Please also avoid repeating all of the results that are presented in the Abstract.

Line 92: delete robust (temper language)

Include study limitations here and in the Methods and Findings section of the Abstract.

Please shorten lines 96-98. Please also note that the general, non-specialist reader would not understand the reference to dolutegravir-based regimens. Please clarify.

Line 113: need a noun after biomedical.

Line 116: please remove formatting error

Line 127—should this be failure rather than success?

There is extensive repetition of concepts in the Introduction. Please reduce repetition in paragraphs 1 and 2 in particular.

The Discussion exceeds 2000 words. Please reduce to 1200.

Comments from Reviewers:

Reviewer #1: The manuscript has been strengthened. There are still a few points that could potentially further improve this study as follows.

1) COVID-19 disaggregated data. Thanks for looking carefully at this. I would suggest adding this to the supplemental data section and noting in the main text that your main findings were robust to this sensitivity analysis.

2) New recommendations (Reviewer 3). I agree with this reviewer that the contributions to the literature could be more explicit. What are things not recommended in Zambian or WHO guidelines that should be? (or put another way, what should health systems be doing differently)

Reviewer #2: Thank you for addressing reviewers' comments throughout the manuscript. This version is much clearer than the prior submission. I have no major comments with this version, but the text is still hard to read in places due to the density of the writing and various grammar issues, missing words, inconsistencies, and some confusing sentences or phrases. My comments here relate to these and are relatively minor. Once addressed, I believe the manuscript can make an important contribution to the literature on patient experience with ART.

Specific Comments:

Abstract

1. Page 2, line 48: "…and included in the analysis…" - it seems a word is missing. Consider adding "were" before "included" here.

2. Page 3, lines 61-62 "…strategies …. should be modernized…." It's not clear what 'modernized strategies" means and whether it refers to client or health systems or something else altogether. Can the authors please clarify this in the manuscript?

Introduction

3. Page 5, line 113. The sentence ending with "… and biomedical" seems to have a word missing (e.g. "factors," "characteristics").

4. The Introduction is generally very dense and a bit hard to understand in places. Simplifying in a few places would improve readability. Examples are 1) lines 125-26: "… delays in returning to care for EAC and poor retention that precludes assessment of virologic status…" (consider: "….delays in returning to care for EAC and poor retention that also delay viral load testing…") and 2) line 130: "By examining transitions through key steps in treatment failure cascade…." (consider "By examining transitions over the course of treatment….").

5. Page 5, line 164: "… were also informally used at the time as well…" - no need for both "also" and "as well."

Methods

6. Page 9, line 227, please add "the" before "second" in the sentence "…. time zero was the date of second consecutive elevated VL…"

7. Page 11, line 256, can the authors please add a reference for the Stata software?

Results

8. Page 14, line 286: assuming the authors mean a viral load test, would recommend adding "test" after "viral load" in the sentence "… (including visits with and without a viral load)…."? There are many references to "VL" that clearly mean "VL test" or a re-test - consider clarifying by adding "test" or "check" (and re-test or re-check).

9. Page 18, line 355: the sentence beginning with "14 (1.8%)…" Suggest revising slightly to "Only 14 (1.8%)…" to avoid starting the sentence with a number.

Discussion

10. Page 22, line 443: the sentence ending with " … are appropriately taken advantage of." Please consider revising to improve grammar: "…. serve to benefit patients optimally."

11. Generally: two writing points: 1) the authors define the acronym VL for viral load but then use the two interchangeably. I suggest using VL consistently rather than using both; and 2) the authors use "VL" frequently to mean "VL test." It's clear what they mean but I still recommend using "test," "check," "re-test," or "re-check" as appropriate for clarity and consistency.

Reviewer #3: My previous comments have been addressed, thank you.

Any attachments provided with reviews can be seen via the following link:

# Author comment: Author response to Decision Letter 3

**DOI:** 10.1371/journal.pmed.1004720.r005

7 Aug 2025

Attachment Submitted filename: Response to reviewers_Gaps in VL monitoring_07_08_2025.docx

# Editor report: Decision Letter 3

**DOI:** 10.1371/journal.pmed.1004720.r006

**Authors:** Alison Farrell

8 Aug 2025

Dear Dr Sikombe, 

On behalf of my colleagues and the Academic Editor, Marie-Louise Newell, I am pleased to inform you that we have agreed to publish your manuscript "Identifying care gaps along the HIV treatment failure cascade: a multi-state analysis of viral load monitoring, re-suppression, and regimen switches in Zambia" (PMEDICINE-D-25-01499R3) in PLOS Medicine.

Before your manuscript can be formally accepted you will need to complete some formatting changes, which you will receive in a follow up email. Please be aware that it may take several days for you to receive this email; during this time no action is required by you. Once you have received these formatting requests, please note that your manuscript will not be scheduled for publication until you have made the required changes.

In the meantime, please log into Editorial Manager at http://www.editorialmanager.com/pmedicine/, click the "Update My Information" link at the top of the page, and update your user information to ensure an efficient production process. 

PRESS

We frequently collaborate with press offices. If your institution or institutions have a press office, please notify them about your upcoming paper at this point, to enable them to help maximise its impact. If the press office is planning to promote your findings, we would be grateful if they could coordinate with medicinepress@plos.org. If you have not yet opted out of the early version process, we ask that you notify us immediately of any press plans so that we may do so on your behalf.

We also ask that you take this opportunity to read our Embargo Policy regarding the discussion, promotion and media coverage of work that is yet to be published by PLOS. As your manuscript is not yet published, it is bound by the conditions of our Embargo Policy. Please be aware that this policy is in place both to ensure that any press coverage of your article is fully substantiated and to provide a direct link between such coverage and the published work. For full details of our Embargo Policy, please visit http://www.plos.org/about/media-inquiries/embargo-policy/.

To enhance the reproducibility of your results, we recommend that you deposit your laboratory protocols in protocols.io, where a protocol can be assigned its own identifier (DOI) such that it can be cited independently in the future. Additionally, PLOS ONE offers an option to publish peer-reviewed clinical study protocols. Read more information on sharing protocols at https://plos.org/protocols?utm_medium=editorial-email&utm_source=authorletters&utm_campaign=protocols

Thank you again for submitting to PLOS Medicine. We look forward to publishing your paper. 

Sincerely, 

Alison Farrell, Ph.D. 

Senior Editor 

PLOS Medicine
